# DeepCC: a novel deep learning-based framework for cancer molecular subtype classification

**DOI:** 10.1038/s41389-019-0157-8

**Published:** 2019-08-16

**Authors:** Feng Gao, Wei Wang, Miaomiao Tan, Lina Zhu, Yuchen Zhang, Evelyn Fessler, Louis Vermeulen, Xin Wang

**Affiliations:** 1 Department of Biomedical Sciences, City University of Hong Kong, Hong Kong SAR, China; 2 Department of Colorectal Surgery, The Sixth Affiliated Hospital, Sun Yat-sen University, Guangzhou, China; 3 Guangdong Institute of Gastroenterology, Guangdong Provincial Key Laboratory of Colorectal and Pelvic Floor Diseases, Supported by National Key Clinical Discipline, Guangzhou, China; 4 Laboratory for Experimental Oncology and Radiobiology (LEXOR), Center for Experimental Molecular Medicine (CEMM), Academic Medical Center (AMC), University of Amsterdam, Amsterdam, the Netherlands; 5 Shenzhen Research Institute, City University of Hong Kong, Shenzhen, China

**Keywords:** Tumour heterogeneity, Cancer genomics, Tumour heterogeneity, Cancer genomics

## Abstract

Molecular subtyping of cancer is a critical step towards more individualized therapy and provides important biological insights into cancer heterogeneity. Although gene expression signature-based classification has been widely demonstrated to be an effective approach in the last decade, the widespread implementation has long been limited by platform differences, batch effects, and the difficulty to classify individual patient samples. Here, we describe a novel supervised cancer classification framework, deep cancer subtype classification (DeepCC), based on deep learning of functional spectra quantifying activities of biological pathways. In two case studies about colorectal and breast cancer classification, DeepCC classifiers and DeepCC single sample predictors both achieved overall higher sensitivity, specificity, and accuracy compared with other widely used classification methods such as random forests (RF), support vector machine (SVM), gradient boosting machine (GBM), and multinomial logistic regression algorithms. Simulation analysis based on random subsampling of genes demonstrated the robustness of DeepCC to missing data. Moreover, deep features learned by DeepCC captured biological characteristics associated with distinct molecular subtypes, enabling more compact within-subtype distribution and between-subtype separation of patient samples, and therefore greatly reduce the number of unclassifiable samples previously. In summary, DeepCC provides a novel cancer classification framework that is platform independent, robust to missing data, and can be used for single sample prediction facilitating clinical implementation of cancer molecular subtyping.

## Introduction

Cancer subtyping is important for selection of patients that benefit most from specified therapies and design of novel targeted agents. Traditionally, cancer classification is largely based on histopathological and clinical characteristics, which makes it difficult to implement uniformly, as individual expertize of the clinicians is often a major determinant^1,2^. Although the prognostic value of these classifications is undisputed, they fall behind in predicting drug efficacy due to the lack of a clear molecular basis. Instead, as an example in colorectal cancer (CRC), genetic features, such as *KRAS* mutation and microsatellite instability (MSI) status^3^, have proven predictive power regarding anti-EGFR and 5-FU efficacy, respectively. However, classifications based on these molecular characteristics still leave much of additional cancer heterogeneity unaccounted for^4^. Therefore, in recent years, whole transcriptome-based cancer subtyping has been widely demonstrated as an efficient approach for dissecting cancer heterogeneity^5^. The evident benefit of this approach is the integration of genetic, epigenetic, and microenvironmental features that impact on cancer biology and clinical presentation. A widely-implemented strategy involves consensus clustering for determination of an optimal number of cancer subgroups, and classification with feature selection, i.e., selection of a list of signature genes^6^.

While several consensus clustering methods^7,8^ have been well established and widely adopted, the classification step suffers several critical limitations. First, a signature gene-based approach places sole emphasis on the role of individual genes, without effective incorporation of biological knowledge such as pathway activity, which often leads to poor interpretability^9–11^. Second, signature genes for classification are not always available due to unpaired gene annotation caused by platform differences, which hampers its portability and translational potential^12^. Last but not least, gene expression profiling is easily affected by factors such as technical platform variation and experimental protocols, leading to nonbiological batch effects^13^. Mathematical and statistical methods might be able to correct for such bias so that data from various sources are comparable. However, such correction methods are not always suitable, especially in situations when the sources of bias are unclear. For instance, the correction power of existing methods such as ComBat^14^ have been demonstrated to be limited to a balanced experimental design^15^. Most critically for clinical implementation, the batch effects also prevent the development of gene expression signature-based classifiers for single sample prediction.

Recent advances in the machine learning community have shown a great promise to apply deep learning for cancer classification. For instance, deep convolutional neural networks have been demonstrated to improve accuracy and reproducibility of tumor classification based on histopathological or radiographic images^16–19^. Deep learning-based frameworks, such as D-GEX^20^, DeepChrome^21^, and DeepSEA^22^ have also been developed for predicting gene expression or effects of noncoding variants based on high-dimensional genomic or epigenomic profiles. Furthermore, several supervised and unsupervised deep learning-based classification methods have been proposed for cancer detection and diagnosis, and they have been demonstrated superior performance over classical methods such as support vector machine (SVM) and random forests (RF)^23–25^. Meanwhile, pathway activities transformed from gene expression profiles have been shown to be more informative and robust for disease classification^26,27^. Motivated by these pioneering works in machine learning, we developed a novel framework Deep Cancer subtype Classification (DeepCC), which leverages both pathway activity transformation and deep learning to address the abovementioned critical limitations in cancer subtype classification.

## Results

### Overall design of DeepCC

**Fig. 1:**
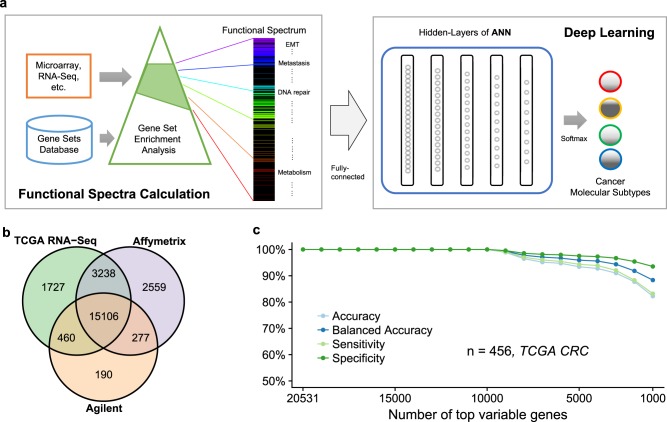
Overview of DeepCC. **a** A deep learning-based cancer classification framework. DeepCC takes as input high-throughput gene expression data and transforms it to functional spectra using gene set enrichment analysis (GSEA). A feedforward artificial neural network is employed subsequently to perform feature learning and build a classifier for cancer classification. **b** Intersection of gene annotations (Entrez IDs) between three technical platforms: TCGA RNA-Seq data set, Affymetrix Human Genome U133 Plus 2.0 array, and Agilent Homo sapiens 37 K DiscoverPrint19742 microarray. **c** DeepCC’s classification performance on subsets of top variable genes, ranging from 1000 to 20,531, selected for calculating functional spectra on the TCGA CRC data set (*n* = 456). The classification performance was evaluated by overall accuracy, mean balanced accuracy, mean sensitivity, and mean specificity

DeepCC is a supervised, biological knowledge-based framework for cancer classification, consisting of two major steps (Fig. 1a):

1. Transformation of high-throughput gene expression data to functional spectra. We first perform gene set enrichment analysis (GSEA)^28^ for each tumor sample’s gene expression profile on thousands of gene sets obtained from public databases such as MSigDB^28^. For each patient sample, the vector of enrichment scores of all gene sets represents a landscape of molecular patterns associating with biological functions, and therefore it is referred to as a functional spectrum.
2. Classification based on deep learning. Taking the obtained functional spectra as input, we next train a classifier using deep learning. By using a trainable multilayer artificial neural network (ANN) to replace hand-engineered features, deep learning takes the advantage of functional spectra, which are more robust and informative. In contrast, feature selection for high-dimensional data is a challenging task for conventional machine learning algorithms, which could lead to bias especially for high-throughput gene expression profiles^29^. To train a DeepCC classifier, we highly recommend employing a widely adopted molecular subtyping system, so that the deep features trained by the ANN can capture most relevant biological properties associated with each molecular subtype. In our case studies, we used the consensus molecular subtyping (CMS) system^30^ for CRC and intrinsic subtyping system for breast cancer (implemented by PAM50^31^), which are both widely adopted in respective fields. The trained DeepCC model can be subsequently used for classification of new samples.

In short, our DeepCC framework has four major advantages over previous cancer subtyping methods:

1. Better interpretability. DeepCC transforms gene expression profiles to functional spectra, which are transcriptomic patterns that have been previously demonstrated to directly associate with biological function. Deep learning is also well known for its capability to learn high-level representations of data through multiple hidden layers^32^. In both case studies, we demonstrated that deep features learned by DeepCC represent characteristic biological processes of different cancer subtypes, which better dissects molecular heterogeneity than gene expression signature-based methods.
2. Platform independency. Different types of high-throughput gene expression data are transformed to the same form of functional spectra, and therefore are directly comparable.
3. More robustness. Since GSEA is performed on the ranks of input data and corrected by its raw value, DeepCC is much less influenced by nonbiological factors such as batch effect and normalization methods.
4. Single sample prediction. DeepCC can deal with single sample gene expression data regardless of the platform by adaptive rescaling to a predefined or user-defined reference (details in “Methods” section). DeepCC single sample predictor (SSP) addresses one critical limitation of previous cancer classification methods, which hampers the translation of cancer molecular subtyping into the clinic.

### Case study in colorectal cancer

To demonstrate the performance of DeepCC, we initially applied it to classify CRC. Recently, we participated in an international CRC subtyping consortium (CRCSC) and identified four consensus molecular subtypes (CMSs)^30^ based on gene expression data of 4151 primary tumor samples from a total of 18 data sets. The four CMSs are characterized by distinct molecular features: CMS1 (MSI immune), CMS2 (canonical), CMS3 (metabolic), and CMS4 (mesenchymal). Also, the CMSs display vastly different clinical features including prognosis and response to therapies. Moreover, together with the CMS taxonomy we reported a 273-gene classifier based on the RF algorithm to facilitate classification of additional data sets^30^.

The translational potential of a signature gene-based classifier is always hampered by missing data due to discrepant gene annotations between different gene expression profiling platforms, leading to poor classification performance^33^. In this case study, we collected 14 publicly available CRC data sets (*n* = 3578) generated from six different microarray/RNA-Seq platforms (Table S1). Comparing Entrez gene annotations between three representative platforms, RNA-Seq for the TCGA CRC data set, Affymetrix Human Genome U133 Plus 2.0 array and Agilent Homo sapiens 37 K DiscoverPrint19742 microarray, we found more than one third of all annotated genes (8451 out of 23,557) are unique to certain platforms (Fig. 1b).

To evaluate the robustness of DeepCC to cross-platform missing genes, we trained a DeepCC classifier for CMS subtypes using TCGA CRC data set (*n* = 456) with CMS subtype information provided by CRCSC^8^, and subsequently studied its performance on expression data of only a subset of genes. More specifically, we iteratively selected a random subset of genes that are most variable across TCGA samples (measured by median absolute deviations), and calculated functional spectra based on the subset of genes selected for classification. Accuracy, balanced accuracy, sensitivity, and specificity were calculated for evaluation of DeepCC’s performance. As expected, we found that DeepCC accomplished a very high accuracy (balanced accuracy > 90%) even when only 2000 genes were used for training the classifier (Fig. 1c), demonstrating DeepCC’s strong robustness to missing data.

**Fig. 2:**
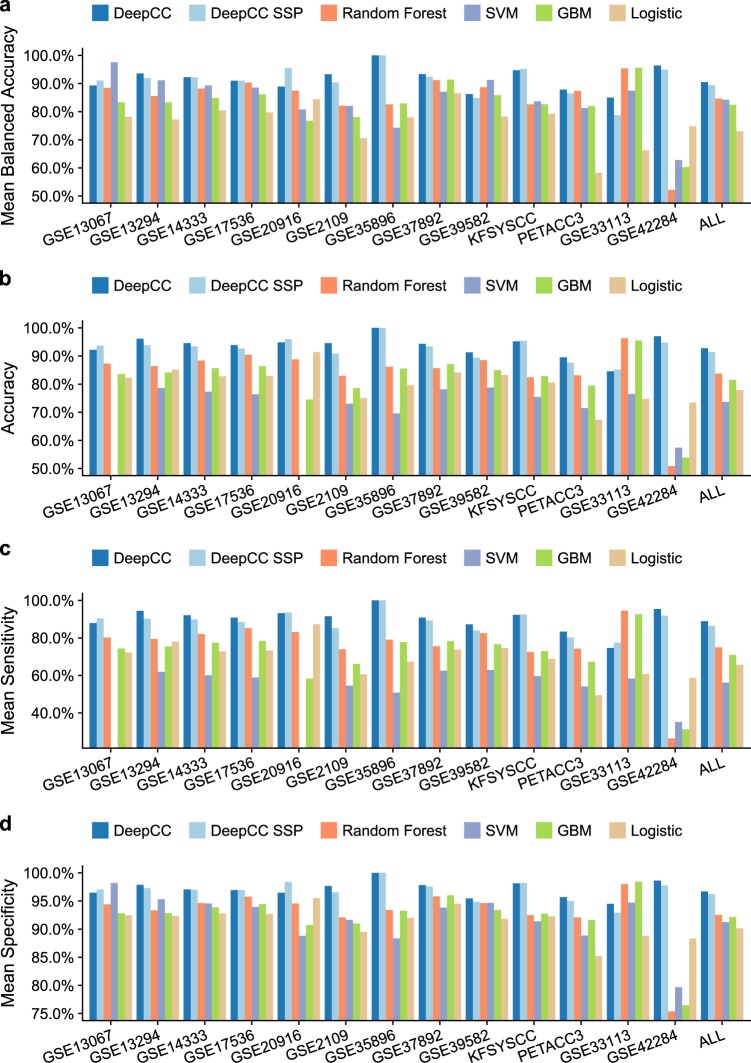
CRC classification performance. Bar plots of classification performance of DeepCC, compared to other signature gene-based approaches. The performance was evaluated on 13 independent validation data sets and the merged data set (ALL), by **a** balanced accuracy (calculated by the mean of balanced accuracy per class), **b** overall accuracy, **c** sensitivity (calculated by the mean of sensitivity per class), and **d** specificity (calculated by the mean of specificity per class)

To comprehensively benchmark the classification performance, we trained a DeepCC classifier using the TCGA RNA-Seq data set with CMS subtype labels, and then applied it to classify 13 other independent data sets based on Affymetrix or Agilent microarray platforms (Table S1), followed by the calculation of sensitivity, specificity, and accuracy based on their original CMS subtyping information. Four widely used signature gene-based classifiers were constructed based on RF, SVM, gradient boosting machine (GBM) and multinomial logistic regression algorithms, respectively. As an intrinsic limitation, we previously reported that the signature gene-based classifier trained on RNA-Seq and Affymetrix microarray platform derived data, showed poor performance on Agilent array derived data^30^. Compared with these signature gene-based classifiers, DeepCC demonstrated higher sensitivity, specificity, and accuracy on the validation data sets (Fig. 2 and Table S2, *P* = 8.47 × 10^−20^, 1.61 × 10^–10^, 3.07 × 10^–21^, 4.56 × 10^–68^, respectively, McNemar’s tests). Notably, DeepCC SSP also showed promising performance as DeepCC (Fig. S1), with remarkably even higher accuracy than the other classifiers (Fig. 2 and Table S2, *P* = 3.08 × 10^–16^, 5.19 × 10^–9^, 6.56 × 10^–17^, 8.2 × 10^–60^, respectively, McNemar’s tests).

**Fig. 3:**
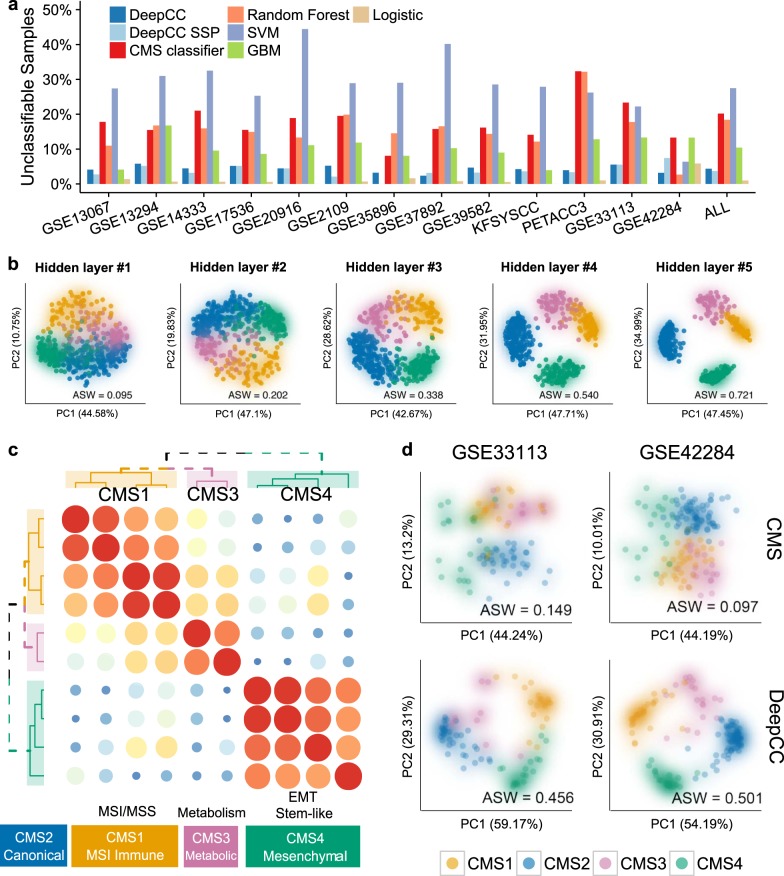
Applying DeepCC to CRC classification. **a** Bar plots of unclassified samples across multiple data sets demonstrating the superior classification performance of DeepCC. The TCGA data set was used to train DeepCC, DeepCC SSP, random forests, SVM, GBM, and multinomial logistic regression classifiers, which were applied to classify 13 independent data sets. In addition, the CMS classifier built by CRCSC was also included for a comparison. **b** Features learned by the hierarchical network of DeepCC showed increasing within-subtype compactness as the hidden layer goes deeper, as indicated by the distributions of CRC samples and average silhouette widths (ASWs) measured in the TCGA data set (*n* = 456). For visualization, the same set of samples were shown in the space of the first two principal components of features learned at each hidden layer (from 1 to 5). **c** Deep feature groups implicate the distinct biological functions associated with CRC subtypes. Deep features were obtained from the last hidden layer of the ANN trained with the TCGA data set (*n* = 456). Clustering of absolute Pearson correlation coefficients between the ten deep features identified three deep feature groups, which are highly correlated with microsatellite instability, metabolic dysregulation, and higher epithelial-to-mesenchymal transition, respectively. The order of deep features is in Fig. S4 and the detailed list of top correlated gene sets for each deep feature is in Table S5. **d** Visualization of patients from two independent validation cohorts in the space of the first two principal components (PCs) of expression data of the 273 CMS signature genes and the ten deep features, respectively. In both data sets, samples are much more tightly distributed within assigned subtypes in the deep feature space than the signature gene space, as quantified by average silhouette width (AWS)^52^

For clinical implementation, it is essential that the proportion of samples that cannot be accurately classified is low. Previously we found that ~20% of CRC samples could not be reliably classified in a single CMS^30^. The question remained if this represented biological and clinical reality, or was a limitation of the classification strategy. Because we detected large variation in the number of unclassifiable samples in the various data sets (range 11–48%)^30^, we suspected that it is predominantly the latter reason paired with data set dependent differences in data quality. Therefore, we hypothesized that DeepCC would be able to reduce the number of unclassifiable samples. To assess classification performance, we used the same criterion that was previously employed for the CMS classifier (posterior probability > 0.5). Across all data sets previously analyzed by CRCSC, DeepCC only failed to classify ~5% tumor samples, much lower than previously reported methods using the same data series (Fig. 3a). To further investigate whether this classification has practical meaning, we performed survival analyses and Fisher’s exact tests for associations with key molecular features (*MSI*, *CIMP*, *CIN*, *P53*, *BRAF*, and *KRAS*) on a public data set (*CIT/GSE39582*, *n* = 557). In the CIT data set, DeepCC can classify 531 out of 557 samples, whereas the CMS classifier based on RF failed to classify 117 out of them. Interestingly, these unclassifiable samples were mainly in the boundary regions of the CMS signature gene space (Fig. S2 and Table S3), suggesting an effect of a suboptimal classification strategy for CMS classifier. Furthermore, the classification results of DeepCC have in general higher associations with molecular markers and clinical outcomes than those of other classifiers (Fig. S3 and Tables S2 and S4). These results demonstrated that DeepCC-based classifications display generally stronger associations with molecular and clinical features, while greatly reduce the number of ‘unclassifiable’ samples.

An important advantage of deep learning is feature learning^34^. The ANN employed by DeepCC learned features through a hierarchy of hidden layers, showing gradual increasing within-subtype compactness, as indicated by the distributions of CRC samples and average silhouette widths (ASWs) measured in the TCGA data set (Fig. 3b). Deep features obtained at the last layer of ANN in DeepCC show distinct patterns across different CRC subgroups (Fig. S4). Especially, after clustering of absolute correlation coefficients between features, three distinct groups of deep features emerged (Fig. 3c). Interestingly, these feature groups are highly correlated with MSI, metabolic dysregulation, and higher epithelial-to-mesenchymal transition (EMT), respectively (Table S5), which summarize the major characteristics of CMS1, CMS3, and CMS4 respectively. CMS2 is a canonical subtype, which is here reinforced by a lack of distinctive feature sets recognized. Moreover, we found that the deep features extracted by DeepCC provide a better representation of patients than the signature genes. In each individual validation data set, we found patient samples are more compactly distributed within assigned subgroups in the space of deep features than in the space of signature genes (two representative examples in Fig. 3d, and the others in Fig. S5). To quantitatively compare the within-class coherence, we calculated ASW of patient samples. We found that the ASWs calculated using deep features are much higher than those based on expression levels of signature genes in all data sets (Figs. 3d, S5, *P* < 0.01, one-sided Wilcoxon signed-rank test). This implicates that DeepCC can find more fundamental functional distinctions between cancer subtypes, which also explains its superior classification performance.

### Case study in breast cancer

**Fig. 4:**
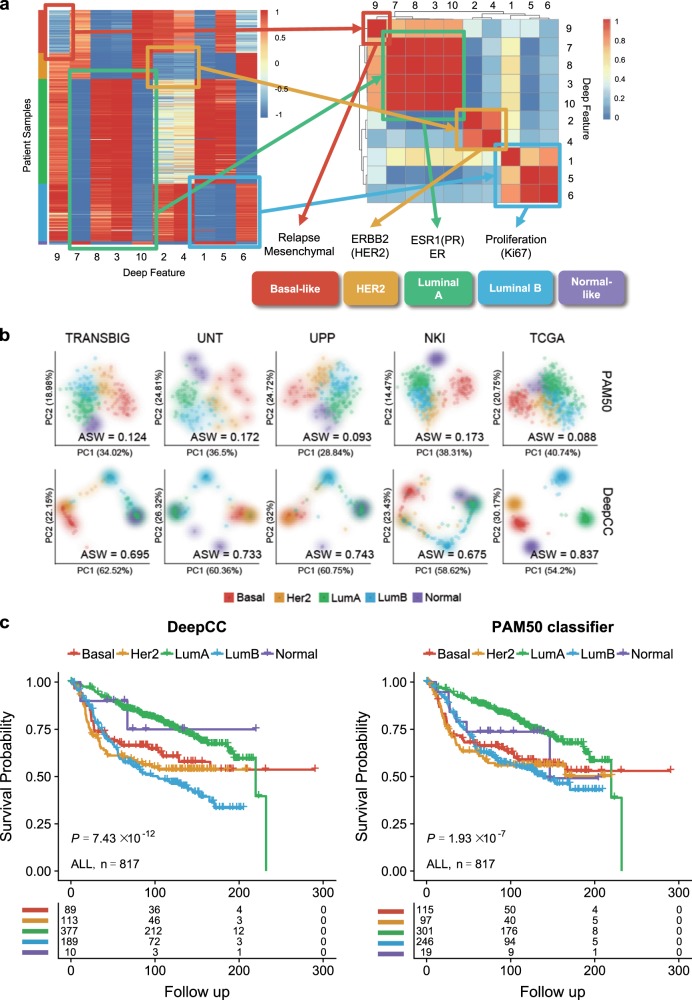
Applying DeepCC to breast cancer data sets. **a** Deep features of breast cancer learned from the TCGA data set (*n* = 517). In the left heatmap, rows represent patient samples, and are ordered by the four CMS subtypes. In the right heatmap, deep features were clustered by the absolute Pearson correlation coefficients between each other. **b** Visualization of patients in five independent breast cancer data sets. The top and bottom rows of figures visualize patients in the spaces of the first two principal components (PCs) of expression data of PAM50 signature genes and the ten deep features, respectively. In each independent data set (*TANSBIG*, *UNT*, *UPP*, *NKI*, and TCGA), samples are much more tightly distributed within assigned subtypes in the deep feature space than the signature gene space, as quantified by average silhouette width (AWS). **c** Kaplan–Meier survival curves of patients in all of four breast cancer data sets (*TANSBIG*, *UNT*, *UPP*, and *NK*). KM plots on the left and right were generated based on classification using DeepCC and the PAM50 classifier, respectively

To evaluate the general applicability of DeepCC to other cancers, we studied breast cancer, another major malignancy with well characterized molecular subtypes. As a reference, we employed PAM50^35^ for intrinsic subtype classification, which is a widely used transcriptome-based classification system. In PAM50, five distinct (intrinsic) molecular subtypes are defined: Basal-like, Her2, Luminal A, Luminal B, and Normal-like. Using TCGA RNA-Seq data set (*n* = 517) with subtyping result predicted by PAM50, we trained a supervised DeepCC classifier to evaluate the classification performance. Similar to the case study in CRC, we first evaluated the robustness of DeepCC classifier to cross-platform missing genes in breast cancer. As expected, DeepCC accomplished a high accuracy (balanced accuracy > 80%) even when only 1000 genes were used for training (Fig. S6). The classifier was subsequently applied to classify four other independent validation data sets (Table S6). DeepCC successfully extracted deep features highly correlated with the underlying biological characteristics of breast cancer subtypes (Fig. 4a and Table S7). Similarly, patient samples showed higher within-class coherence in the deep feature space than in the signature gene space, as suggested by the much higher ASWs (Fig. 4b, *P* < 0.01, one-sided Wilcoxon signed-rank test). Furthermore, survival analyses on the four validation sets (*TANSBIG*, *UNT*, *UPP*, and *NKI*) separately and jointly demonstrated that DeepCC classification has higher associations with disease-free survival than PAM50 (Figs. 4c, S7 and Table S8). The clinical relevance is also supported by the significant associations (all *P* < 10^–12^, Fisher’s exact tests) between the Luminal and HER2 subtypes predicted by DeepCC with ER/PR and HER2 receptor status, which are their corresponding characteristic markers, respectively (Table S9).

## Discussion

Major malignancies such as breast and CRCs have been demonstrated to be molecularly heterogeneous, which directly relates to diverse patient outcomes in the clinic. The last decade has seen rich literature for dissecting molecular heterogeneity of cancers, including our own work on colon cancer^30,36,37^. The common drawback of all these studies, however, lies in the limitations of the employed gene expression signature-based classification approach: platform dependency, poor robustness to batch effects, and lack of capability for single sample classification. In this study, to address these challenges we developed a novel supervised framework DeepCC, which leverages the power of deep learning based on an ANN for cancer classification. DeepCC transforms gene expression profiles to transcriptional patterns with functional relevance using GSEA, followed by deep learning using a trainable multilayer ANN.

We demonstrated the superior performance of DeepCC to other popular classification methods using multiple independent gene expression data sets involving over 4000 patient samples in a recent study by the CRCSC^30^. We found that cancer patients are much more compactly distributed in the deep feature space than in the signature gene space, demonstrating the superior classification performance of DeepCC compared with other conventional methods. Of the utmost translational importance is the ability of DeepCC to successfully classify single samples. Furthermore, to show the general applicability of this new classification framework, we effectively applied DeepCC to breast cancer, and demonstrated a better performance over PAM50, which is a widely used classification system in breast cancer.

It should be noted that DeepCC is a supervised, biological knowledge-based framework specifically developed for addressing current challenges in classification but not clustering of cancer patients. In the last decades, molecular subtyping for major malignancies has been extensively studied, and many subtyping systems have been well established. However, implementing these subtyping systems is challenged by the abovementioned limitations of classical classification methods, which motivated us to develop DeepCC. In practice, it is recommended to use a widely adopted molecular subtyping systems, such as the CMS^30^ for CRC and intrinsic subtyping for breast cancer (implemented by PAM50^31^), to train a DeepCC classifier for a particular cancer type. The trained DeepCC model can be subsequently used for classification of new samples, facilitating real clinical implementation of cancer subtyping considering its superior performance, cross-platform robustness and capability for single sample prediction, as demonstrated in our case studies.

There are various molecular testing assays such as Mammaprint^31^, BluePrint^38^, and Oncotype DX^39^, which have been well established and already applied in clinical practice. However, MammaPrint, and Oncotype DX are prognostic tools for prediction of high-, (intermediate-), and low-risk of recurrence and/or metastasis, which are not strictly ‘biological’ subtypes. BluePrint can only predict Luminal, HER2, and Basal subtypes, but cannot distinguish between Luminal A and Luminal B subtypes. Instead, PAM50 classification system encompasses all the five intrinsic molecular subtypes of breast cancer, and therefore is an ideal subtyping system for training DeepCC. Similarly, a number of risk scoring assays for CRC recurrence prediction have been developed, including Oncotype DX for Colon Cancer^39^, ColonPRS^40^, ColoPrint^41^, GeneFx Colon^42^, OncoDefender-CRC^43^, ColoGuideEx^44^, ColoGuidePro^45^, and miRNA recurrence classifier^46^. All these assays have demonstrated prognostic values in independent patient series, but are not defining or predicting molecular subtypes of CRC. Therefore, in our CRC case study we selected the widely adopted CMS system developed by CRCSC to train DeepCC.

Since DeepCC employs GSEA to transform gene expression data to functional spectra, the prior knowledge of gene sets needs to be comprehensive to avoid potential bias. Of special importance are the major characteristic signaling pathways or biological processes for each cancer subtype, which should be included in the gene set database. In practice, it is highly recommended to use a database with a large scale of curated gene sets that are of high quality such as MSigDB v6.0 database employed in our case studies, which includes 17,779 gene sets encompassing all cancer hallmark signaling pathways.

In conclusion, our deep learning-based framework DeepCC integrates biological knowledge, overcomes limitations of signature gene-based approach and leads to more robust performance. Through case studies on CRC and breast cancer, we also demonstrated its superior classification performance and clinical relevance. The capability of DeepCC to reliably classify single samples, using transcriptome data obtained by any platform will greatly facilitate the translation of molecular subtyping into clinical practice.

## Materials and methods

### Data processing

#### Colon cancer data sets

In this study, we analyzed 14 independent CRC data sets, involving in total 3578 primary tumor samples (Table S1). TCGA CRC set was employed as our training cohort, and the corresponding gene expression data (level 3 RNA-Seq data) were downloaded from Firehose Broad GDAC portal (http://gdac.broadinstitute.org/). For patient samples with both gene expression data based on Illumina GA and Hi-Seq platforms, we only kept the Hi-Seq version. Scaled estimates in the gene-level RSEM files were first converted to TPM (transcripts per million) by multiplying with 10^6^ and then log2-transformed.

For validation, 11 out of 13 data sets were curated by *CRCSC* (*n* *=* 2 674)^8^, including *GSE13067* (*n* *=* 73)*, GSE13294* (*n* *=* 155)*, GSE14333* (*n* *=* 157), *GSE17536* (*n* *=* 174), *GSE20916* (*n* *=* 90), *GSE2109* (*n* *=* 287), *GSE35896* (*n* *=* 62), *GSE37892* (*n* *=* 127), *GSE39582* (*n* *=* 557), *KFSYSCC* (*n* *=* 305), and *PETACC3* (*n* *=* 687). These curated gene expression data were downloaded from the official repository of CRCSC on Synapse (https://www.synapse.org/#!Synapse:syn2623706/wiki/). More details about the curation procedures can be found in Guinney et al.^30^, which resulted in expression levels of 5973 genes for each data set. The other two validation data sets, *GSE42284* (*n* *=* 188), *GSE33113* (*n* *=* 90), were downloaded from GEO directly in its processed form, using Bioconductor package ‘GEOquery’. The *GSE42284* data set is based on Agilent Homo sapiens 37 K DiscoverPrint_19742 microarray platform, including 188 CRC patient samples, processed by Agilent Feature Extraction software based on MedianSignal output and normalized by Lowess normalization^47^. Probeset IDs were converted to gene symbols based on the corresponding gene annotations (GPL16280), and then further converted to Entrez IDs using Bioconductor package ‘org.Hs.eg.db’. The *GES33113* data set is based on Affymetrix Human Genome U133 Plus 2.0 array. The *GSE33113* data set includes 90 CRC patient samples and 6 normal samples, and was processed by MAS5.0 normalization and GCOS software^36^. We only kept colorectal samples, and then converted all probeset IDs to Entrez IDs based on the corresponding gene annotations (GPL570).

In addition, CMS classification labels associated with all samples were also obtained from CRCSC Synapse repository (https://www.synapse.org/#!Synapse:syn2623706/wiki/).

More details about the CRC data sets can be found in Table S1.

#### Breast cancer data sets

Four breast cancer data sets (*TANSBIG*, *UNT*, *UPP*, and *NKI*) were downloaded from Bioconductor (http://www.bioconductor.org/) in their processed form.

For MACQ II BR cohort, the raw data were downloaded from GEO database with the accession number GSE20194 in R using ‘GEOquery’ package. The *GSE20194* data set is based on Affymetrix Human Genome U133A Array, including 230 breast cancer patient samples, normalized using MAS5.0 method. Probeset IDs were converted to gene symbols based on corresponding gene annotations (GPL96), and then converted to Entrez IDs using Bioconductor package ‘org.Hs.eg.db’.

In the breast cancer case study, PAM50 labels for the TCGA BRCA cohort obtained from TCGA^48^ were used for training and validation.

More details about the breast cancer data sets can be found in Table S6.

### Functional spectra

A functional spectrum is a list of *Enrichment Scores* calculated by GSEA^28^. The following steps are used to calculate the enrichment score ***ES***:

1. Filter duplicate probes of gene expression to prevent overestimation.
2. Calculate log2 fold changes D of N genes by subtracting the background signal.
3. Rank order D to form the gene list L = {g_1_,…,g_N_}, which is in a descending order.
4. Form a list S = {s1,…,sN} containing the contribution of each gene for the enrichment score. For a gene ghit in the gene set C, we score it by $\frac{{\left| {g_{hit}} \right|}}{{\mathop {\sum }\nolimits_{g_i \in C} \left| {g_i} \right|}}$; for a gene not in the gene set C, we score it by $- \frac{1}{{N - \mathop {\sum }\nolimits_{g_i \in C} 1}}$.
5. Calculate the accumulation sum from s1 in S to obtain the deviation, and the value deviated the most from zero is the enrichment score ES.

In this study, we used all 17,779 gene sets in MSigDB^28^ v6.0 (downloaded on 1 Jun 2017).

### Deep learning implementation

The deep learning framework in DeepCC was implemented based on MXNet (https://arxiv.org/abs/1512.01274) incorporating the latest optimization methods developed by deep learning community, which can run on both CPU and GPU. By default, DeepCC builds a fully connected multilayer perceptron (feedforward neural network)^49^ using the architecture of hidden layers: 2000, 500, 120, 30, 10 with Tanh activation function. The last layer for output is SoftMax. The whole network is initialized using Xavier^50^. The optimizer can be chosen from SDG^51^ (learning rate = 0.01, momentum = 0.9) or AdaDelta (https://arxiv.org/abs/1212.5701).

### DeepCC classifier and single sample predictor (SSP)

To predict an individual sample, DeepCC SSP calculates the functional spectrum using a user-customized or a predefined reference, which is averaged gene expression profile over all samples for a specific cancer type in TCGA. First, we keep overlapped genes and rescale the input gene expression profile to the reference by fitting a linear model using the ‘lm’ function of R package ‘stats’. A functional spectrum is subsequently calculated using the residuals obtained from the linear regression, which is used as the input into the trained DeepCC classifier for classification.

### Other classification approaches and evaluation metrics

To compare DeepCC with signature gene-based approaches, we employed center-normalized expression data for the 273 CMS signature genes to build classifiers using different classification algorithms, including RF, SVM, GBM and the multinomial logistic regression model.

Four statistical measures were used to evaluate the classification performance. For each CMS subtype, we first calculate true positive (TP), false negative (FN), false positive (FP), and true negative (TN), and then calculate the following measures:

1. Mean of sensitivity (per class). For each class, the sensitivity was calculated using TP/(TP + FN).
2. Mean of specificity (per class). For each class, the specificity was calculated using TN/(TN + FP).
3. Mean of balanced accuracy (per class). For each class, the balanced accuracy was calculated using (sensitivity + specificity)/2.
4. Accuracy. The overall accuracy is the total number of correct predictions divided by the total number of patient samples.

### Functional analysis and visualization

To identify highly correlated biological functions of each subtype, we first calculated Pearson correlation coefficients between deep features extracted by DeepCC and enrichment scores of gene sets in the training data set. We obtained a correlation matrix indicating the relationships between deep features and functional gene sets. Highly correlated gene sets, either positively or negatively, indicate biological functions highly associated with deep features (Tables S4 and S7).

To visualize classification results by DeepCC and signature gene-based approaches, we projected all patients into the deep feature space and the signature gene space, respectively. For DeepCC, the first two principal components of the ten deep features were visualize in a two-dimensional space, For the other classification approaches, the first two principal components of the expression levels of signature genes were visualized in a two-dimensional space.

### Code availability

#### R package

DeepCC was implemented as an R package, and the source code and instructions for running DeepCC locally are available at GitHub.

Project name: DeepCC

Project home page: https://cityuhk-compbio.github.io/DeepCC/

Operating system(s): Platform independent

Programming language: R and C

Other requirements: R version 3.3 or higher; MXNet version 0.10, or higher

License: MIT

#### Online platform

An online platform of DeepCC (https://cityuhk-compbio.github.io/deepcc_online/) is also provided for transformation of gene expression profiles of tumor samples to functional spectra and prediction of cancer subtypes based on pretrained models.

### Statistics

All statistical analyses were performed using R (version 3.3.3; http://www.r-project.org) and *P* < 0.05 was considered as significant in all cases. RF, SVM, GBM, and the multinomial logistic regression model were implemented by ‘randomForest’, ‘kernlab’, ‘gbm’, and ‘glmnet’ package respectively in R. Radial kernel and probability model was used for SVM. All other parameters were kept as default in each respective package. The linear model used in DeepCC SSP was implemented by the ‘lm’ function of R package ‘stats’. The comparison of classification results was based on McNemar’s chi-squared test in R, implemented by the ‘mcnemar.test’ function in ‘stats’ package. Analyses of disease-free survival data were performed using R package ‘survival’ and *p*-values were derived from log-rank tests. AWSs were used as the quantification of the relevance between features and CMS subtypes, calculated by the function ‘silhouette’ in R package ‘cluster’. Fisher’s exact tests, *t*-tests, Wilcoxon signed-rank tests, Pearson correlation coefficients were calculated using R package ‘stats’.

## Supplementary information

Supplementary figures.

Supplementary tables.
