# Supplementary material for: DeepCC: a novel deep learning-based framework for cancer molecular subtype classification
Source: Oncogenesis. 2019 Aug 16;8(9):44. doi: 10.1038/s41389-019-0157-8 (PMC6697729; doi:10.1038/s41389-019-0157-8)
Supplement: Supplementary file 1 — Supplementary figures. [file 41389_2019_157_MOESM1_ESM.pdf]

Supplementary figures

Figure S1: DeepCC robustly predicted CMS subtypes for individual samples Confusion matrices on the classification results based on (**a**) DeepCC and (**b**) DeepCC SSP on 2 323 samples merged from all the 13 validation data sets. Both DeepCC and DeepCC SSP classifications achieved high concordance with the reference (P < 0.001, Fisher's exact tests), which are CMS labels provided by CRCSC.

Figure S2: Visualization of patients in the CIT/GSE39582 data set Patient samples visualized in the space of the first two principal components (PCs) of (**a**) the expression levels of 273 CMS signature genes, and (**b**) deep features extracted by DeepCC. Unclassified samples by the CMS classifier were denoted by gray plus symbols in both (**a**)and (**b**).

Figure S3: Kaplan-Meier survival curves of patients (CIT/GSE39582) Curves were generated based on classification using (**a**) DeepCC, (**b**) DeepCC SSP, (**c**) Random Forest, (**d**) SVM, (**e**) GBM and (f) multinomial logistic regression classifier, respectively.

Figure S4: Deep features of CRC learned from the TCGA data set (n = 456) The rows represent patient samples, grouped by the training labels of patients, and the columns are the ten deep features extracted from the last hidden layer of the trained ANN.

Figure S5: Visualization of patients in various independent colon cancer data sets The top and bottom rows of figures visualize patients in the spaces of the first two principal components (PCs) of expression data of the 273 CMS signature genes and the 10 deep features, respectively. In each independent data set, samples are much more tightly distributed within assigned subtypes in the deep feature space than the signature gene space, as quantified by average Silhouette width (AWS).

Figure S6: DeepCC's classification performance to cross-platform missing genes in breast cancer DeepCC was trained by top variable genes,

selecting for calculating functional spectra on the TCGA BRCA data set (n = 517), and ranging from 1 000 to 20 531. The classification performance was evaluated by overall accuracy, mean balanced accuracy, mean sensitivity and mean specificity.

Figure S7: Kaplan-Meier survival curves of patients in four independent breast cancer data sets (TANSBIG, UNT, UPP, NK) KM plots on the left and right were generated based on classification using (**a, c, e, g**) DeepCC, and (**b, d, f, h**) the PAM50 classifier, respectively.

Figure S1: DeepCC robustly predicted CMS subtypes for individual samples

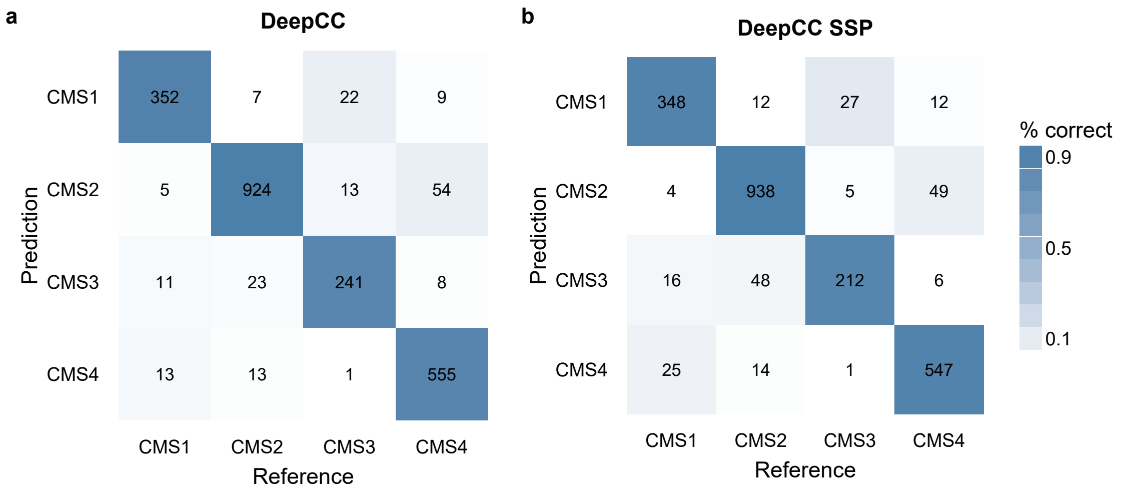

Figure S2: Visualization of patients in the CIT/GSE39582 data set

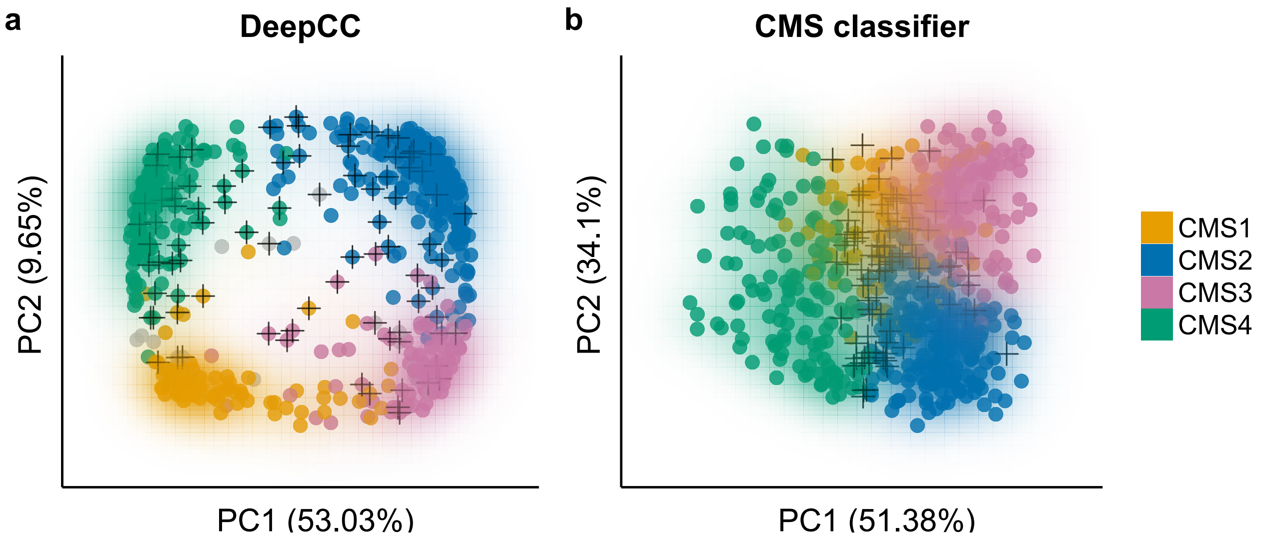

Figure S3: Kaplan-Meier survival curves of patients (CIT/GSE39582)

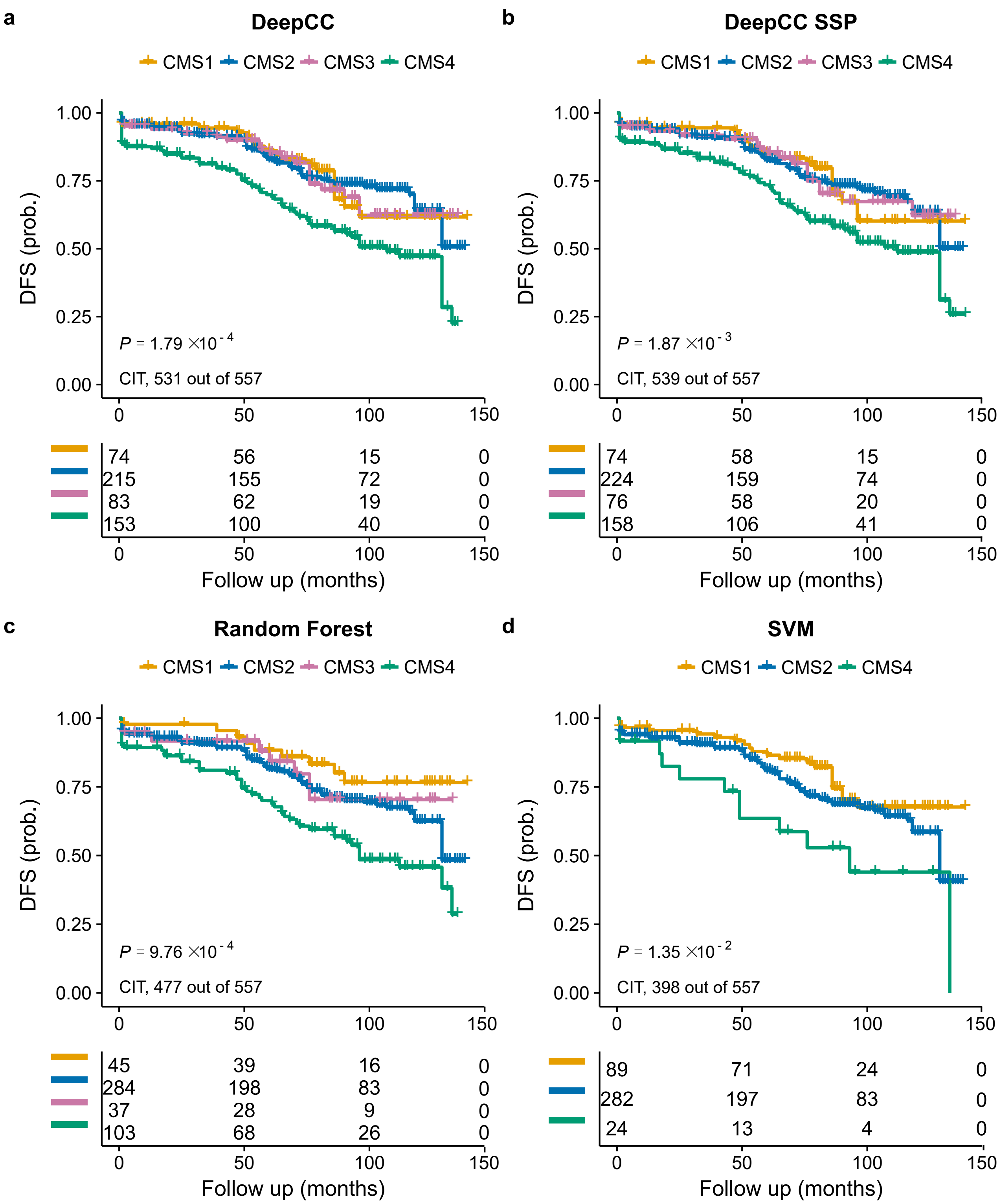

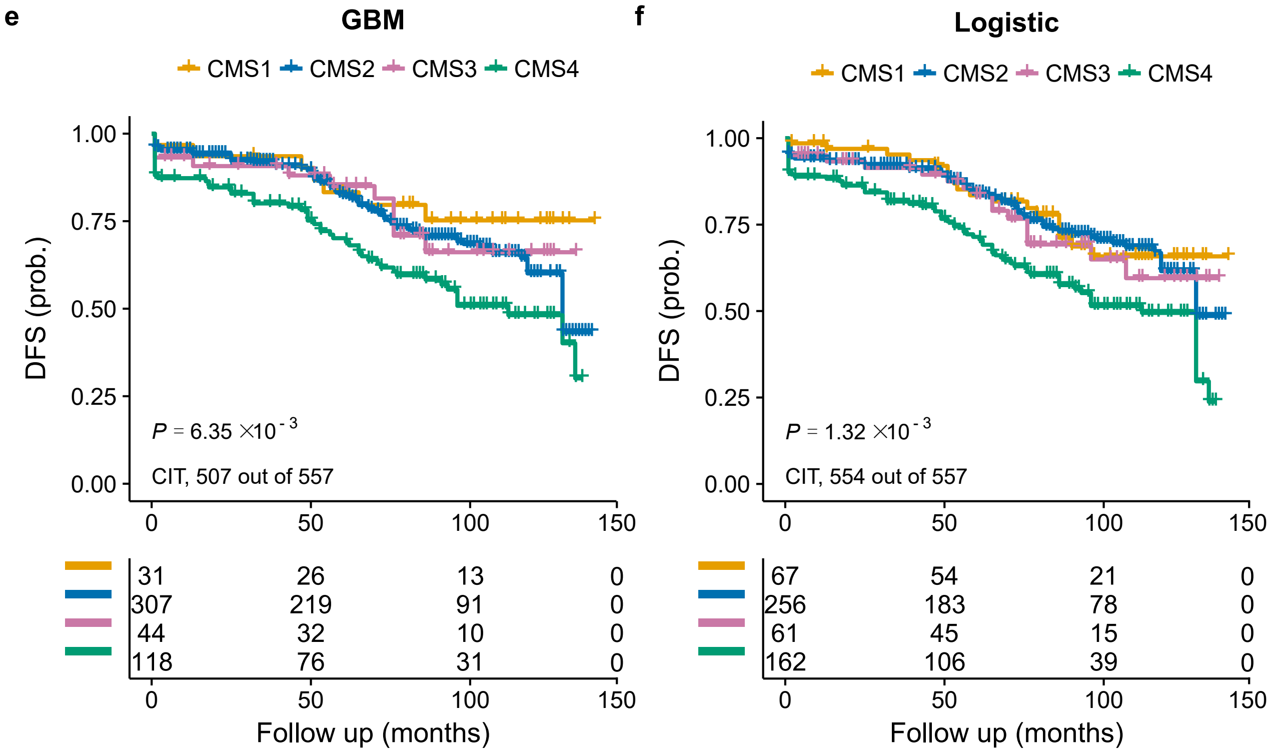
e
GBM
CMS1 CMS2 CMS3 CMS4
DFS (prob.)
1.00
0.75
0.50
0.25
0.00
P = 6.35 ×10⁻³
CIT, 507 out of 557
0 50 100 150
31 26 13 0
307 219 91 0
44 32 10 0
118 76 31 0
Follow up (months)
f
Logistic
CMS1 CMS2 CMS3 CMS4
P = 1.32 ×10⁻³
CIT, 554 out of 557
67 54 21 0
256 183 78 0
61 45 15 0
162 106 39 0
Follow up (months)

Figure S4: Deep features of CRC learned from the TCGA data set (n = 456)

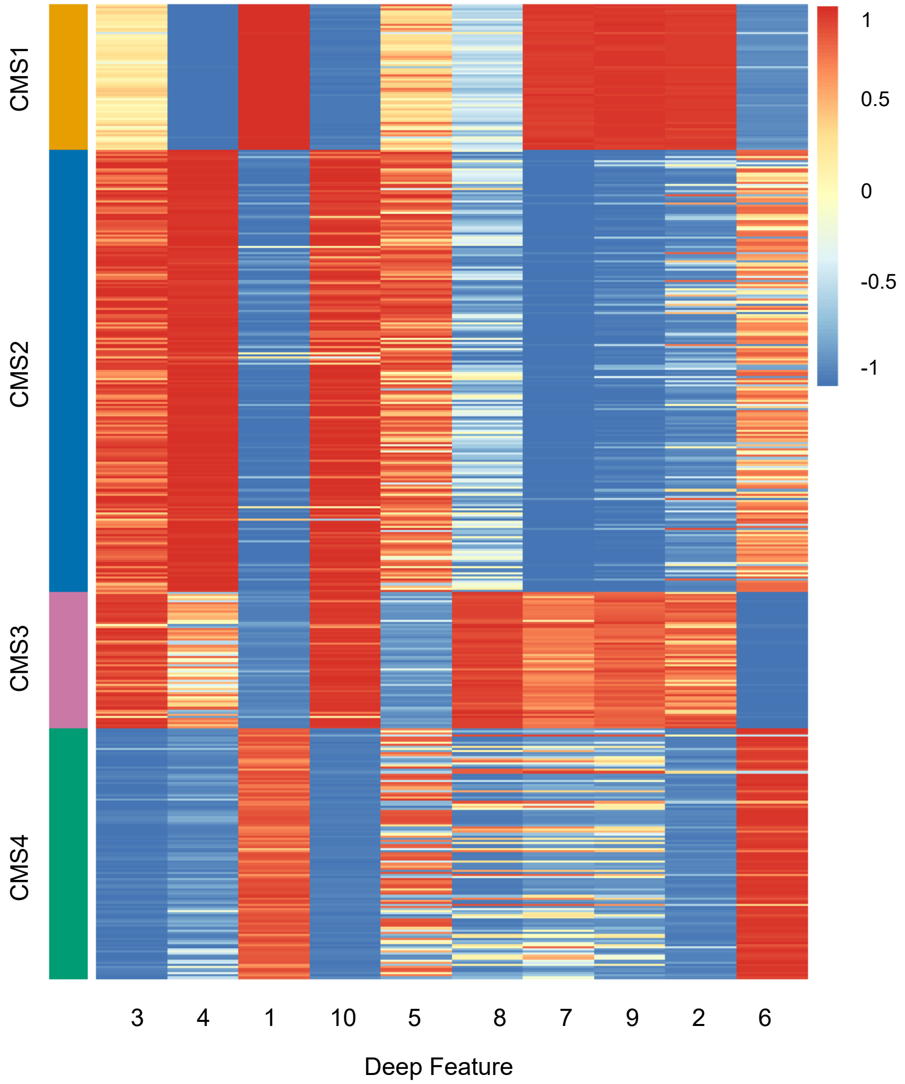

Figure S5: Visualization of patients in various independent colon cancer data sets

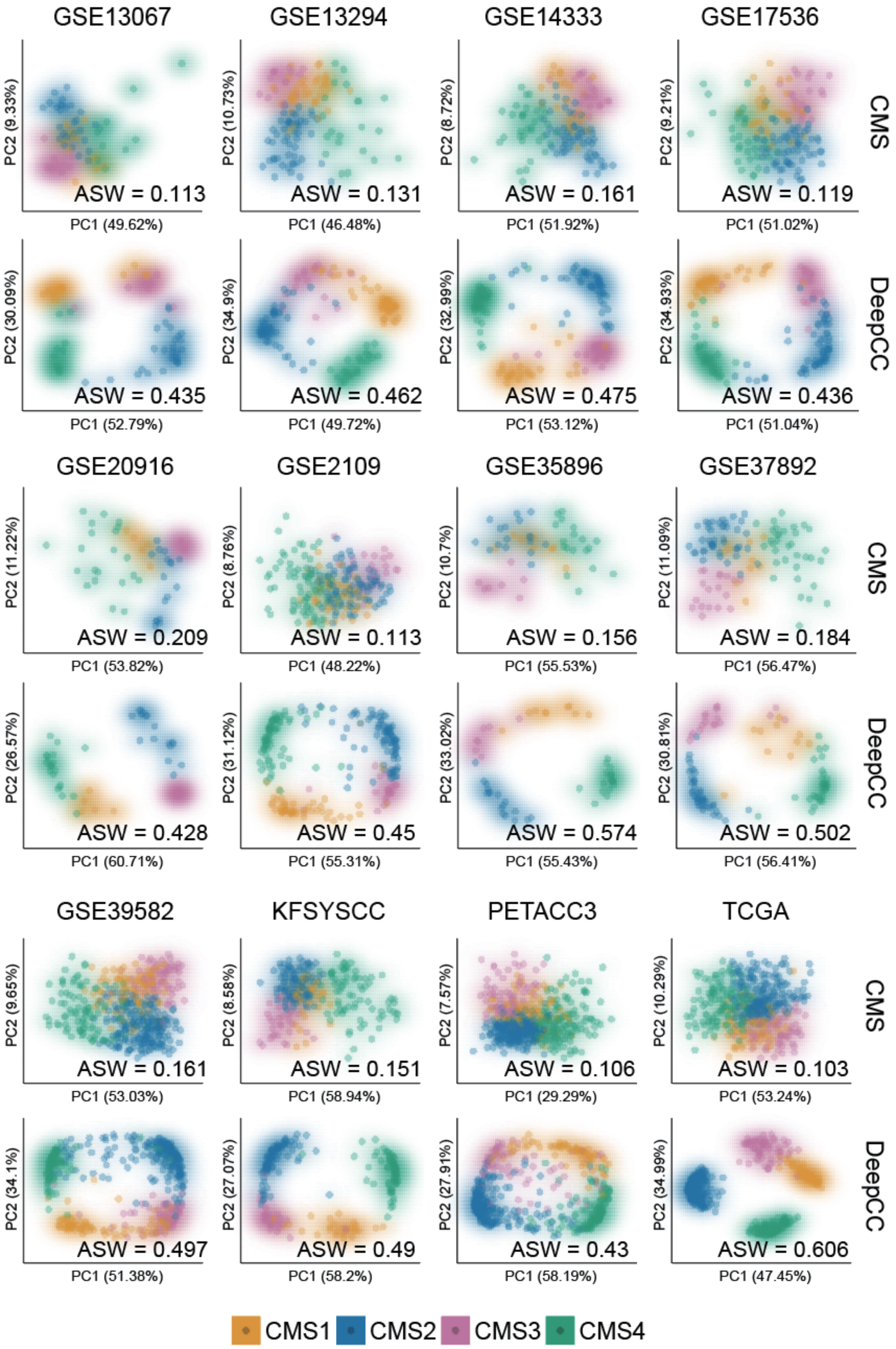

Figure S6: DeepCC's classification performance of breast cancer on subsets of top variable genes

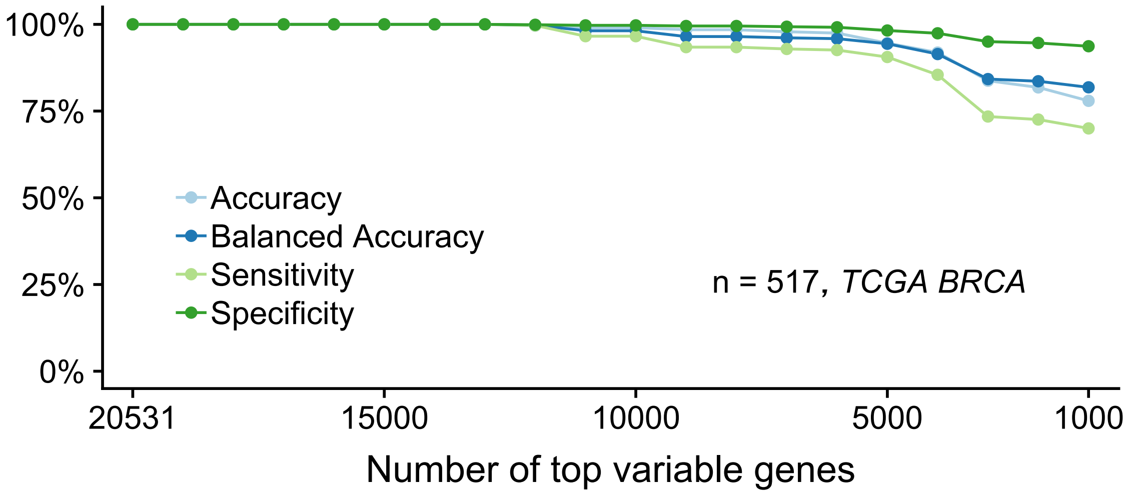

Figure S7: Kaplan-Meier survival curves of patients in four independent breast cancer data sets (*TANSBIG, UNT, UPP, NK*)

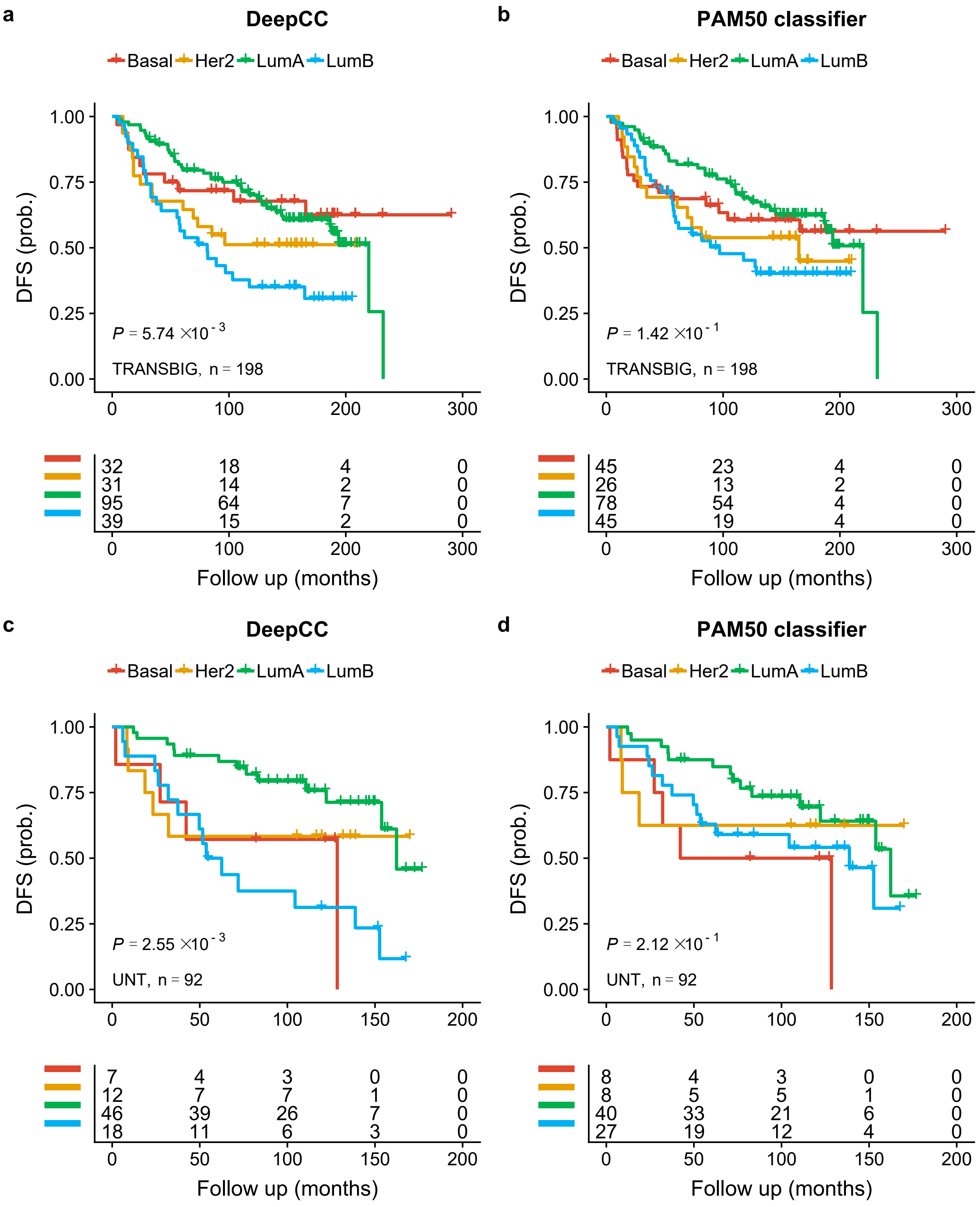

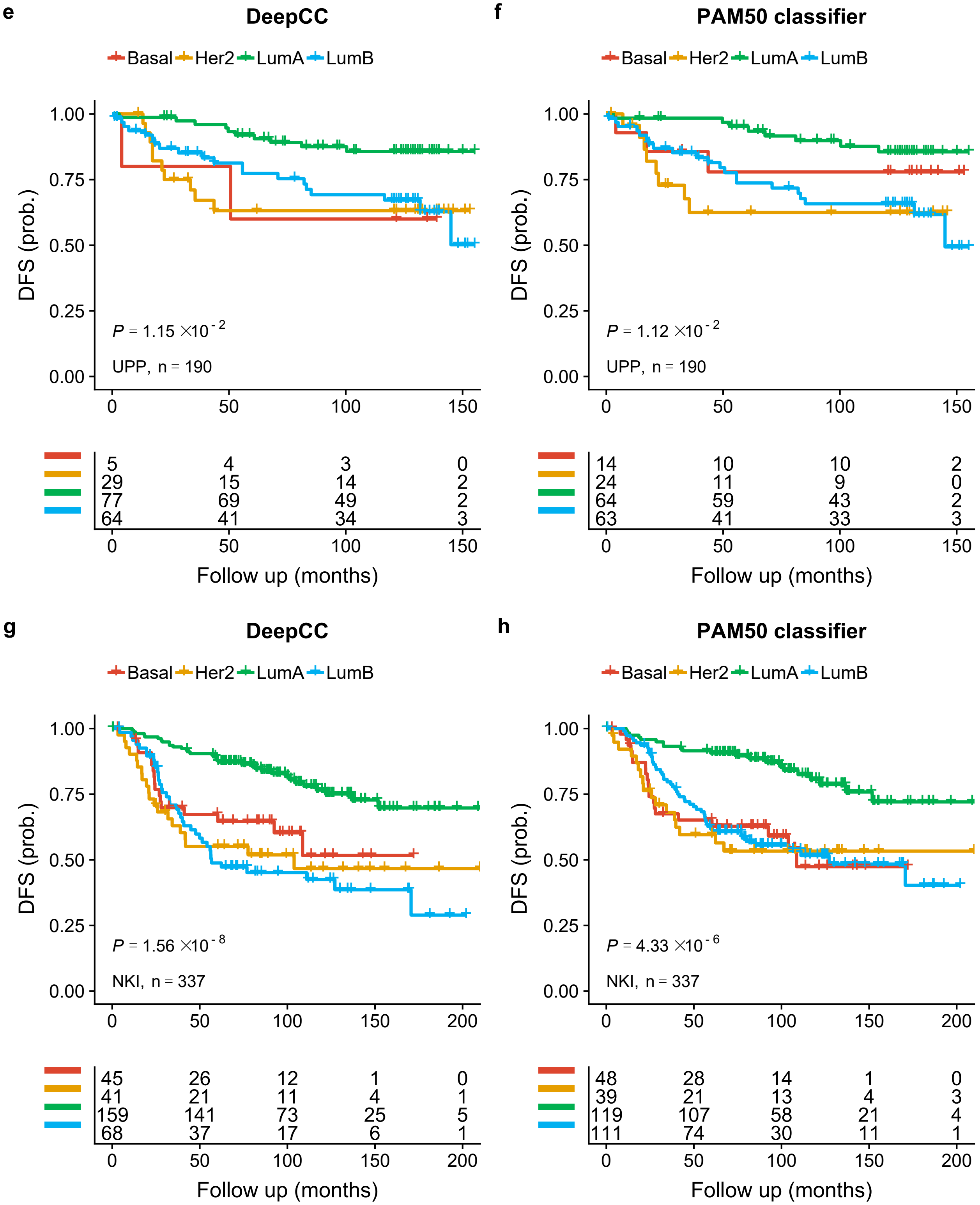
e
DeepCC
Basal Her2 LumA LumB
DFS (prob.)
P = 1.15 ×10^-2
UPP, n = 190
5 4 3 0
29 15 14 2
77 69 49 2
64 41 34 3
Follow up (months)
f
PAM50 classifier
Basal Her2 LumA LumB
DFS (prob.)
P = 1.12 ×10^-2
UPP, n = 190
14 10 10 2
24 11 9 0
64 59 43 2
63 41 33 3
Follow up (months)
g
DeepCC
Basal Her2 LumA LumB
DFS (prob.)
P = 1.56 ×10^-8
NKI, n = 337
45 26 12 1 0
41 21 11 4 1
159 141 73 25 5
68 37 17 6 1
Follow up (months)
h
PAM50 classifier
Basal Her2 LumA LumB
DFS (prob.)
P = 4.33 ×10^-6
NKI, n = 337
48 28 14 1 0
39 21 13 4 3
119 107 58 21 4
111 74 30 11 1
Follow up (months)
