# Supplementary material for: DeepCC: a novel deep learning-based framework for cancer molecular subtype classification
Source: Oncogenesis. 2019 Aug 16;8(9):44. doi: 10.1038/s41389-019-0157-8 (PMC6697729; doi:10.1038/s41389-019-0157-8)
Supplement: Supplementary file 2 — Supplementary tables. [file 41389_2019_157_MOESM2_ESM.pdf]

Supplementary Tables

1 Table S1 Summary of CRC data sets 2

2 Table S2 CRC classification performance 3

3 Table S3 CIT (GSE39582) classification results 6

4 Table S4 CIT (GSE39582) clinical relevance 17

5 Table S5 CRC top correlated biological features 20

6 Table S6 Summary of breast cancer data sets 26

7 Table S7 Breast cancer top correlated biological features 27

8 Table S8 Breast cancer classification results 33

9 Table S9 Breast cancer data sets clinical relevance 60

1 Table S1 Summary of CRC data sets

Dataset	Platform	Tissue	Total	with CMS*	Source	Number of unique genes (EnrezID)
TCGA	Illumina GA/HiSeq RNA-Seq	Fresh frozen	626	456	TCGA	20531
GSE13067	Affymetrix HG133plus2	Fresh frozen	73	56	CRCSC	5973
GSE13294	Affymetrix HG133plus2	Fresh frozen	155	124	CRCSC	5973
GSE14333	Affymetrix HG133plus2	Fresh frozen	157	129	CRCSC	5973
GSE17536	Affymetrix HG133plus2	Fresh frozen	174	144	CRCSC	5973
GSE20916	Affymetrix HG133plus2	Fresh frozen	90	45	CRCSC	5973
GSE2109	Affymetrix HG133plus2	Fresh frozen	287	239	CRCSC	5973
NKIAZ (GSE35896)	Affymetrix HG133plus2	Fresh frozen	62	51	CRCSC	5973
GSE37892	Affymetrix HG133plus2	Fresh frozen	127	105	CRCSC	5973
CIT (GSE39582)	Affymetrix HG133plus2	Fresh frozen	557	458	CRCSC	5973
KFSYSCC	Affymetrix HG133plus2	Fresh frozen	305	227	CRCSC	5973
PETACC3	Almac's Affymetrix ADXCRC	Fresh frozen	687	526	CRCSC	5973
AMC (GSE33113)	Affymetrix HG133plus2	Fresh frozen	90	80	GEO	21180
Agendia (GSE42284)	Agilent 37K discoverprint_19742	Fresh frozen	188	139	GEO	16033
Total			3578	2779		23557

Table S1: Summary of CRC data sets

*Reference:

1. Guinney, J. et al. The consensus molecular subtypes of colorectal cancer. Nat. Med. (2015). doi:10.1038/nm.3967

2 Table S2 CRC classification performance

	Accuracy	Balanced Accuracy	Sensitivity	Specificity
GSE13067	0.893	0.922	0.879	0.965
GSE13294	0.935	0.961	0.944	0.979
GSE14333	0.922	0.946	0.921	0.97
GSE17536	0.91	0.939	0.908	0.969
GSE20916	0.889	0.948	0.932	0.965
GSE2109	0.933	0.946	0.915	0.977
GSE35896	1	1	1	1
GSE37892	0.933	0.943	0.908	0.978
GSE39582	0.862	0.913	0.872	0.954
KFSYSCC	0.947	0.952	0.923	0.981
PETACC3	0.878	0.895	0.834	0.957
GSE33113	0.85	0.846	0.746	0.945
GSE42284	0.964	0.97	0.954	0.986
ALL	0.905	0.928	0.889	0.967

Table S2.1: DeepCC

	Accuracy	Balanced Accuracy	Sensitivity	Specificity
GSE13067	0.911	0.937	0.904	0.97
GSE13294	0.919	0.938	0.902	0.973
GSE14333	0.922	0.935	0.899	0.97
GSE17536	0.91	0.927	0.884	0.969
GSE20916	0.956	0.96	0.935	0.984
GSE2109	0.904	0.908	0.851	0.965
GSE35896	1	1	1	1
GSE37892	0.924	0.934	0.893	0.976
GSE39582	0.849	0.894	0.84	0.949
KFSYSCC	0.952	0.954	0.925	0.982
PETACC3	0.865	0.876	0.802	0.95
GSE33113	0.787	0.852	0.774	0.929
GSE42284	0.95	0.948	0.918	0.977
ALL	0.894	0.913	0.864	0.962

Table S2.2: DeepCC SSP

	Accuracy	Balanced Accuracy	Sensitivity	Specificity
GSE13067	0.885	0.873	0.802	0.944
GSE13294	0.856	0.864	0.795	0.933
GSE14333	0.882	0.884	0.821	0.946
GSE17536	0.903	0.905	0.852	0.958
GSE20916	0.875	0.888	0.831	0.946
GSE2109	0.821	0.83	0.739	0.921
GSE35896	0.826	0.862	0.79	0.934
GSE37892	0.912	0.857	0.756	0.958
GSE39582	0.887	0.886	0.825	0.946
KFSYSCC	0.826	0.825	0.725	0.925
PETACC3	0.873	0.831	0.742	0.921
GSE33113	0.954	0.963	0.946	0.98
GSE42284	0.522	0.509	0.264	0.754
ALL	0.847	0.837	0.75	0.925

Table S2.3: Random Forest

	Accuracy	Balanced Accuracy	Sensitivity	Specificity
GSE13067	0.976			0.982
GSE13294	0.911	0.786	0.619	0.953
GSE14333	0.893	0.773	0.6	0.946
GSE17536	0.886	0.764	0.589	0.94
GSE20916	0.808			0.888
GSE2109	0.82	0.731	0.545	0.916
GSE35896	0.743	0.696	0.508	0.884
GSE37892	0.871	0.782	0.625	0.938
GSE39582	0.913	0.788	0.629	0.947
KFSYSCC	0.837	0.755	0.596	0.914
PETACC3	0.813	0.715	0.541	0.889
GSE33113	0.875	0.765	0.583	0.947
GSE42284	0.628	0.574	0.352	0.797
ALL	0.842	0.737	0.562	0.912

Table S2.4: SVM

	Accuracy	Balanced Accuracy	Sensitivity	Specificity
GSE13067	0.833	0.836	0.744	0.928
GSE13294	0.833	0.841	0.754	0.929
GSE14333	0.849	0.857	0.775	0.939
GSE17536	0.862	0.864	0.783	0.944
GSE20916	0.767	0.745	0.583	0.907
GSE2109	0.781	0.786	0.661	0.91
GSE35896	0.83	0.855	0.778	0.933
GSE37892	0.914	0.871	0.783	0.96
GSE39582	0.859	0.85	0.765	0.934
KFSYSCC	0.826	0.829	0.73	0.928
PETACC3	0.82	0.795	0.673	0.916
GSE33113	0.956	0.955	0.926	0.984
GSE42284	0.603	0.539	0.312	0.765
ALL	0.825	0.815	0.709	0.921

Table S2.5: GBM

	Accuracy	Balanced Accuracy	Sensitivity	Specificity
GSE13067	0.782	0.823	0.722	0.925
GSE13294	0.772	0.852	0.78	0.923
GSE14333	0.805	0.828	0.728	0.928
GSE17536	0.797	0.83	0.732	0.927
GSE20916	0.844	0.914	0.873	0.955
GSE2109	0.706	0.75	0.606	0.895
GSE35896	0.78	0.797	0.673	0.92
GSE37892	0.865	0.841	0.737	0.945
GSE39582	0.783	0.832	0.746	0.918
KFSYSCC	0.793	0.806	0.689	0.923
PETACC3	0.582	0.673	0.495	0.852
GSE33113	0.662	0.748	0.608	0.888
GSE42284	0.748	0.735	0.586	0.883
ALL	0.731	0.779	0.656	0.902

Table S2.6: Logistic regression model

2.0.0.1 McNemar's test

Compare the classification results of DeepCC with Random Forest, SVM, GBM, Logistic regression model, McNemar's test shows p = 8.47×10^{-20}, 1.61×10^{-10}, 3.07×10^{-21}, 4.56×10^{-68}.
Compare the classification results of DeepCC SSP with Random Forest, SVM, GBM, Logistic regression model, McNemar's test shows p = 3.08×10^{-16}, 5.19×10^{-9}, 6.56×10^{-17}, 8.2×10^{-60}.

3 Table S3 CIT (GSE39582) classification results

	CMS	DeepCC	Random Forest	Random Forest	SVM	GBM	Logistic regression model
GSM971957		CMS4	CMS4			CMS4	CMS4
GSM971958	CMS4	CMS4	CMS4	CMS4		CMS4	CMS4
GSM971959	CMS3					CMS3	CMS3
GSM971960	CMS2	CMS2	CMS2	CMS2	CMS2	CMS2	CMS2
GSM971961	CMS4	CMS4	CMS4	CMS4		CMS4	CMS4
GSM971962	CMS4	CMS4	CMS4	CMS4		CMS4	CMS4
GSM971963	CMS1	CMS1	CMS1	CMS1	CMS1	CMS1	CMS1
GSM971964	CMS2	CMS2	CMS2	CMS2	CMS2	CMS2	CMS2
GSM971965	CMS2	CMS4	CMS4		CMS2	CMS4	CMS4
GSM971966		CMS4	CMS4			CMS4	CMS4
GSM971967	CMS3	CMS3	CMS3	CMS3		CMS3	CMS3
GSM971968	CMS4	CMS4	CMS4	CMS4	CMS4	CMS4	CMS4
GSM971969	CMS1		CMS1	CMS2		CMS2	CMS2
GSM971970	CMS3	CMS3	CMS3		CMS1		CMS1
GSM971971	CMS1	CMS3	CMS3			CMS2	CMS2
GSM971972	CMS2		CMS2	CMS2	CMS2	CMS2	CMS1
GSM971973	CMS1	CMS1	CMS1		CMS1	CMS1	CMS4
GSM971974	CMS3	CMS3	CMS4			CMS3	CMS3
GSM971975	CMS2	CMS2	CMS2	CMS2	CMS2	CMS2	CMS2
GSM971976	CMS2	CMS2	CMS2	CMS2	CMS2	CMS2	CMS2
GSM971977			CMS4	CMS4		CMS4	CMS4
GSM971978	CMS1	CMS1	CMS1		CMS1		CMS1
GSM971979	CMS1	CMS1	CMS1	CMS1	CMS1		CMS1
GSM971980	CMS1	CMS1	CMS1	CMS1	CMS1		CMS3
GSM971981	CMS1	CMS1	CMS1		CMS1	CMS4	CMS1
GSM971982	CMS1	CMS1	CMS1	CMS4	CMS1	CMS4	CMS4
GSM971983	CMS1	CMS1	CMS1	CMS1	CMS1		CMS1
GSM971984	CMS1	CMS1	CMS1		CMS1		CMS1
GSM971985	CMS3	CMS3	CMS3	CMS3		CMS3	CMS2
GSM971986		CMS4	CMS4		CMS2	CMS4	CMS4
GSM971987	CMS4	CMS4	CMS4	CMS4		CMS4	CMS4
GSM971988	CMS1	CMS3		CMS1	CMS1		CMS1
GSM971989	CMS3	CMS3	CMS3			CMS3	CMS3
GSM971990						CMS2	CMS2
GSM971991		CMS4	CMS4	CMS2	CMS2	CMS2	CMS4
GSM971992	CMS2	CMS2	CMS2	CMS2	CMS2	CMS2	CMS2
GSM971993		CMS3	CMS3	CMS2		CMS2	CMS3
GSM971994	CMS2	CMS2	CMS2	CMS2	CMS2	CMS2	CMS2
GSM971995		CMS2	CMS2	CMS2	CMS2	CMS2	CMS2
GSM971996	CMS4	CMS4	CMS4	CMS4	CMS4	CMS4	CMS4
GSM971997	CMS1	CMS1	CMS1	CMS1	CMS1		CMS1
GSM971998		CMS2	CMS2	CMS2	CMS2	CMS2	CMS2
GSM971999	CMS2	CMS4	CMS4	CMS2	CMS2	CMS2	CMS2
GSM972000	CMS2	CMS2	CMS2	CMS2	CMS2	CMS2	CMS2
GSM972001		CMS2	CMS2	CMS2	CMS2	CMS2	CMS2
GSM972002	CMS3	CMS3	CMS3	CMS3	CMS1		CMS3
GSM972003	CMS2		CMS2	CMS2	CMS2	CMS2	CMS2
GSM972004	CMS2	CMS2	CMS2	CMS2	CMS2	CMS2	CMS2
GSM972005	CMS3	CMS3	CMS3	CMS3		CMS3	CMS3
GSM972006	CMS4	CMS4	CMS4	CMS2	CMS2	CMS4	CMS2

GSM972007	CMS4	CMS4	CMS4		CMS4		CMS4			CMS4
GSM972008	CMS4	CMS1	CMS1		CMS4	CMS1	CMS4			CMS4
GSM972009		CMS4	CMS4							CMS2
GSM972010	CMS4	CMS4	CMS4		CMS2					CMS4
GSM972011	CMS4	CMS4	CMS4		CMS4	CMS4	CMS4			CMS4
GSM972012	CMS3	CMS3	CMS3				CMS2			CMS3
GSM972013	CMS2	CMS2	CMS2		CMS2	CMS2	CMS2			CMS2
GSM972014	CMS2	CMS2	CMS2		CMS2	CMS2	CMS2			CMS2
GSM972015	CMS1	CMS1	CMS1		CMS1	CMS1	CMS1			CMS1
GSM972016	CMS1	CMS1				CMS1				CMS1
GSM972017	CMS2	CMS4	CMS4		CMS2	CMS2	CMS2			CMS2
GSM972018	CMS2	CMS4	CMS4		CMS2	CMS2	CMS2			CMS4
GSM972019	CMS4	CMS4	CMS4		CMS4		CMS4			CMS4
GSM972020	CMS2	CMS4	CMS4			CMS2	CMS2			CMS4
GSM972021	CMS1	CMS1	CMS1			CMS1				CMS1
GSM972022	CMS2	CMS4	CMS4		CMS2	CMS2	CMS2			CMS2
GSM972023	CMS3	CMS4	CMS4		CMS4					CMS4
GSM972024	CMS2	CMS2	CMS2		CMS2	CMS2	CMS2			CMS2
GSM972025	CMS2				CMS2	CMS2	CMS2			CMS4
GSM972026	CMS4	CMS4	CMS4		CMS4		CMS4			CMS4
GSM972027	CMS2	CMS4	CMS4		CMS2	CMS2	CMS2			CMS4
GSM972028	CMS2	CMS4	CMS4		CMS2	CMS2	CMS2			CMS2
GSM972029	CMS4	CMS4	CMS4		CMS4		CMS4			CMS4
GSM972030		CMS4	CMS4		CMS4	CMS4	CMS4			CMS4
GSM972031	CMS2	CMS2	CMS2		CMS2	CMS2	CMS2			CMS4
GSM972032	CMS2	CMS2	CMS2		CMS2	CMS2	CMS2			CMS2
GSM972033	CMS2	CMS2	CMS2		CMS2	CMS2	CMS2			CMS2
GSM972034	CMS2		CMS2		CMS2		CMS2			CMS2
GSM972035	CMS2	CMS2	CMS2		CMS2	CMS2	CMS2			CMS2
GSM972036	CMS3	CMS3	CMS3		CMS2	CMS2	CMS2			CMS2
GSM972037	CMS3	CMS3	CMS3				CMS2			CMS2
GSM972038	CMS2	CMS2	CMS2		CMS2	CMS2	CMS2			CMS2
GSM972039	CMS4	CMS4	CMS4		CMS4	CMS4	CMS4			CMS4
GSM972040	CMS2	CMS2	CMS2		CMS2	CMS2	CMS2			CMS2
GSM972041	CMS2	CMS2	CMS2		CMS2	CMS2	CMS2			CMS2
GSM972042	CMS3	CMS3	CMS3		CMS3		CMS3			CMS3
GSM972043	CMS2	CMS2	CMS2		CMS2	CMS2	CMS2			CMS2
GSM972044	CMS3	CMS3	CMS3		CMS3		CMS3			CMS3
GSM972045	CMS3	CMS3	CMS3				CMS3			CMS3
GSM972046	CMS4	CMS4	CMS4				CMS2			CMS4
GSM972047		CMS4	CMS4		CMS2	CMS2	CMS2			CMS4
GSM972048	CMS2	CMS2	CMS2		CMS2	CMS2	CMS2			CMS2
GSM972049	CMS3	CMS3	CMS3		CMS3		CMS3			CMS3
GSM972050	CMS3	CMS3	CMS3		CMS3		CMS3			CMS2
GSM972051	CMS4	CMS4	CMS4		CMS4		CMS4			CMS4
GSM972052	CMS2	CMS2	CMS2		CMS2	CMS2	CMS2			CMS2
GSM972053	CMS2	CMS2	CMS2		CMS2	CMS2	CMS2			CMS2
GSM972054	CMS4	CMS4	CMS4		CMS4		CMS4			CMS4
GSM972055		CMS4	CMS4		CMS4		CMS4			CMS4
GSM972056	CMS1	CMS1	CMS1		CMS1	CMS1				CMS1
GSM972057	CMS2	CMS2	CMS2		CMS2	CMS2	CMS2			CMS2
GSM972058	CMS1	CMS1	CMS1		CMS1	CMS1	CMS1			CMS1
GSM972059	CMS1	CMS4	CMS1		CMS4	CMS1	CMS4			CMS4
GSM972060	CMS2	CMS2	CMS2		CMS2	CMS2	CMS2			CMS2

GSM972061	CMS2	CMS2	CMS4	CMS2	CMS2	CMS2	CMS2
GSM972062	CMS2	CMS2	CMS2	CMS2	CMS2	CMS2	CMS2
GSM972063		CMS4	CMS4	CMS4		CMS4	CMS2
GSM972064	CMS4	CMS4	CMS4	CMS4		CMS4	CMS4
GSM972065	CMS2	CMS2	CMS2	CMS2	CMS2	CMS2	CMS2
GSM972066	CMS2	CMS2	CMS2	CMS2	CMS2	CMS2	CMS2
GSM972067	CMS4	CMS4	CMS4	CMS4		CMS4	CMS4
GSM972068	CMS4	CMS4	CMS4	CMS4	CMS4	CMS4	CMS4
GSM972069		CMS2	CMS2	CMS2	CMS2	CMS2	CMS2
GSM972070		CMS2	CMS2		CMS2	CMS2	CMS2
GSM972071	CMS2	CMS2	CMS2	CMS2	CMS2	CMS2	CMS4
GSM972072	CMS1	CMS1	CMS1	CMS1	CMS1	CMS4	CMS1
GSM972073	CMS4	CMS4	CMS4	CMS4		CMS4	CMS4
GSM972074	CMS2	CMS2	CMS2	CMS2	CMS2	CMS2	CMS2
GSM972075	CMS1	CMS1	CMS1	CMS2	CMS1	CMS2	CMS3
GSM972076	CMS1	CMS1	CMS1		CMS1	CMS4	CMS4
GSM972077		CMS4	CMS4	CMS4		CMS4	CMS4
GSM972078	CMS2	CMS4	CMS4	CMS4		CMS4	CMS4
GSM972079				CMS4	CMS4	CMS4	CMS4
GSM972080	CMS1	CMS1	CMS1	CMS1	CMS1		CMS1
GSM972081	CMS2	CMS2	CMS2	CMS2	CMS2	CMS2	CMS2
GSM972082	CMS4	CMS4	CMS4	CMS4	CMS4	CMS4	CMS4
GSM972083		CMS4	CMS4	CMS2	CMS2	CMS2	CMS2
GSM972084	CMS4	CMS4	CMS4	CMS4		CMS4	CMS4
GSM972085	CMS2	CMS2	CMS2	CMS2	CMS2	CMS2	CMS2
GSM972086	CMS2	CMS2	CMS2	CMS2	CMS2	CMS2	CMS2
GSM972087	CMS2	CMS2	CMS2	CMS2	CMS2	CMS2	CMS2
GSM972088		CMS4	CMS4	CMS4		CMS4	CMS4
GSM972089	CMS4	CMS4	CMS4	CMS4	CMS4	CMS4	CMS4
GSM972090	CMS2	CMS2	CMS2	CMS2	CMS2	CMS2	CMS2
GSM972091	CMS3	CMS3	CMS3	CMS2			CMS2
GSM972092	CMS4	CMS4	CMS4	CMS4		CMS4	CMS4
GSM972093		CMS4	CMS4	CMS4		CMS4	CMS4
GSM972094		CMS4	CMS4	CMS4		CMS4	CMS4
GSM972095		CMS4	CMS4	CMS4		CMS4	CMS4
GSM972096	CMS2		CMS2	CMS2	CMS2	CMS2	CMS2
GSM972097	CMS4	CMS4	CMS4	CMS4	CMS4	CMS4	CMS4
GSM972098	CMS3	CMS3	CMS3	CMS3		CMS3	CMS3
GSM972099	CMS2	CMS2	CMS2	CMS2	CMS2	CMS2	CMS2
GSM972100	CMS3	CMS3	CMS3			CMS2	CMS3
GSM972101		CMS3	CMS3		CMS2	CMS2	CMS2
GSM972102		CMS4	CMS4			CMS4	CMS4
GSM972103		CMS1	CMS4				
GSM972104	CMS4	CMS4	CMS4	CMS4		CMS4	CMS4
GSM972105	CMS2	CMS4	CMS4	CMS2	CMS2	CMS2	CMS4
GSM972106	CMS2	CMS4	CMS4		CMS2	CMS2	CMS4
GSM972107	CMS2	CMS4	CMS4	CMS2	CMS2	CMS2	CMS4
GSM972108	CMS2	CMS2	CMS2	CMS2	CMS2	CMS2	CMS2
GSM972109	CMS1	CMS3	CMS3		CMS1		CMS3
GSM972110	CMS3	CMS3	CMS3	CMS2		CMS2	CMS2
GSM972111	CMS3	CMS3	CMS3	CMS2	CMS2	CMS2	CMS3
GSM972112	CMS4	CMS4	CMS4	CMS4	CMS4	CMS4	CMS4
GSM972114	CMS3	CMS3	CMS3		CMS1		CMS4
GSM972115	CMS2	CMS2	CMS2	CMS2	CMS2	CMS2	CMS2

GSM972116		CMS4				CMS4	CMS1
GSM972117			CMS1		CMS1	CMS1	CMS1
GSM972118		CMS2	CMS2	CMS2		CMS2	CMS1
GSM972119	CMS2	CMS2	CMS2	CMS2	CMS2	CMS2	CMS2
GSM972120		CMS1	CMS1	CMS2			CMS4
GSM972121	CMS3	CMS3	CMS3	CMS3			CMS3
GSM972122	CMS2	CMS2	CMS2	CMS2	CMS2	CMS2	CMS2
GSM972123	CMS2	CMS2	CMS2	CMS2	CMS2	CMS2	CMS2
GSM972124	CMS2	CMS2	CMS2	CMS2	CMS2	CMS2	CMS2
GSM972125	CMS4	CMS4	CMS4	CMS4		CMS4	CMS4
GSM972126	CMS2	CMS2	CMS2	CMS2	CMS2	CMS2	CMS2
GSM972127		CMS4	CMS4			CMS4	CMS4
GSM972128		CMS4	CMS4	CMS4		CMS4	CMS4
GSM972129		CMS1	CMS3		CMS1	CMS2	CMS1
GSM972130	CMS2	CMS2	CMS2	CMS2	CMS2	CMS2	CMS2
GSM972131	CMS4	CMS4	CMS4	CMS4		CMS4	CMS4
GSM972132		CMS2	CMS2	CMS4		CMS4	CMS2
GSM972133	CMS4	CMS4	CMS4	CMS4		CMS4	CMS4
GSM972134		CMS4	CMS4		CMS2	CMS2	CMS3
GSM972135	CMS3	CMS3	CMS3	CMS3		CMS3	CMS2
GSM972136	CMS2	CMS2	CMS2	CMS2	CMS2	CMS2	CMS2
GSM972137	CMS2	CMS2	CMS2	CMS2	CMS2	CMS2	CMS2
GSM972138	CMS2		CMS2	CMS2	CMS2	CMS2	CMS2
GSM972139	CMS2	CMS2	CMS2	CMS2	CMS2	CMS2	CMS2
GSM972140	CMS4	CMS4	CMS4	CMS4	CMS4	CMS4	CMS4
GSM972141		CMS4	CMS4	CMS2	CMS2	CMS2	CMS4
GSM972142	CMS2	CMS2	CMS2	CMS2	CMS2	CMS2	CMS2
GSM972143	CMS2	CMS4	CMS4	CMS2	CMS2	CMS2	CMS2
GSM972144	CMS2	CMS2	CMS2	CMS2	CMS2	CMS2	CMS2
GSM972145		CMS2	CMS2	CMS2	CMS2	CMS2	CMS2
GSM972146		CMS4	CMS4		CMS4	CMS4	CMS4
GSM972147	CMS2	CMS2	CMS4	CMS2	CMS2	CMS2	CMS4
GSM972148	CMS2	CMS2	CMS2	CMS2	CMS2	CMS2	CMS2
GSM972149	CMS2	CMS2	CMS2	CMS2	CMS2	CMS2	CMS2
GSM972150	CMS2	CMS2	CMS2	CMS2	CMS2	CMS2	CMS2
GSM972151	CMS2	CMS2	CMS2	CMS2	CMS2	CMS2	CMS2
GSM972152	CMS2	CMS2	CMS2	CMS2	CMS2	CMS2	CMS2
GSM972153	CMS2	CMS2	CMS2	CMS2	CMS2	CMS2	CMS2
GSM972154	CMS2	CMS3	CMS3		CMS2	CMS2	CMS3
GSM972155	CMS2	CMS3	CMS2		CMS2	CMS2	CMS2
GSM972156	CMS2	CMS2	CMS2	CMS2	CMS2	CMS2	CMS2
GSM972157	CMS2	CMS4	CMS2	CMS2	CMS2	CMS2	CMS4
GSM972158	CMS4	CMS4	CMS4	CMS4		CMS4	CMS4
GSM972159	CMS3	CMS3	CMS3	CMS3		CMS2	CMS3
GSM972160	CMS2	CMS4	CMS4	CMS2	CMS2	CMS2	CMS2
GSM972161		CMS2	CMS2	CMS2	CMS2	CMS2	CMS2
GSM972162	CMS4	CMS4	CMS4	CMS4		CMS4	CMS4
GSM972163		CMS2	CMS2	CMS2	CMS2	CMS2	CMS2
GSM972164		CMS4	CMS4		CMS2	CMS2	CMS4
GSM972165		CMS4	CMS4	CMS4		CMS4	CMS4
GSM972166	CMS2		CMS4		CMS2	CMS2	CMS3
GSM972167	CMS2	CMS2	CMS2	CMS2	CMS2	CMS2	CMS2
GSM972168	CMS1	CMS1	CMS1	CMS1	CMS1		CMS1
GSM972169	CMS2	CMS2	CMS2	CMS2	CMS2	CMS2	CMS2

GSM972170	CMS2	CMS4	CMS4	CMS2	CMS2	CMS2	CMS4
GSM972171	CMS1	CMS1	CMS1	CMS1	CMS1	CMS4	CMS1
GSM972172		CMS3	CMS2	CMS2	CMS2	CMS2	CMS3
GSM972173	CMS2	CMS2	CMS2	CMS2	CMS2	CMS2	CMS2
GSM972174	CMS2	CMS2	CMS2	CMS2	CMS2	CMS2	CMS2
GSM972175	CMS2	CMS2	CMS2	CMS2	CMS2	CMS2	CMS2
GSM972176	CMS4	CMS4	CMS4	CMS4		CMS4	CMS4
GSM972178	CMS3	CMS3	CMS3	CMS3		CMS3	CMS3
GSM972179		CMS4	CMS4	CMS4		CMS2	CMS4
GSM972180	CMS1	CMS1	CMS1	CMS1	CMS1	CMS1	CMS1
GSM972181	CMS4	CMS4	CMS4	CMS4	CMS4	CMS4	CMS4
GSM972182	CMS2	CMS2	CMS2	CMS2	CMS2	CMS2	CMS2
GSM972183	CMS3	CMS3	CMS3	CMS3		CMS3	CMS3
GSM972184		CMS4	CMS4	CMS4	CMS4	CMS4	CMS4
GSM972185	CMS2	CMS2	CMS2	CMS2	CMS2	CMS2	CMS2
GSM972186	CMS4	CMS4	CMS4	CMS4		CMS4	CMS4
GSM972187	CMS4	CMS4	CMS4	CMS4		CMS4	CMS4
GSM972188	CMS2	CMS2	CMS2	CMS2	CMS2	CMS2	CMS4
GSM972189		CMS1	CMS1	CMS2		CMS2	CMS2
GSM972190	CMS2	CMS2	CMS2	CMS2	CMS2	CMS2	CMS2
GSM972191	CMS3	CMS3	CMS3	CMS2	CMS2	CMS2	CMS3
GSM972192		CMS1	CMS4				CMS4
GSM972193	CMS2	CMS2	CMS2	CMS2	CMS2	CMS2	CMS3
GSM972194	CMS2	CMS2	CMS2	CMS2	CMS2	CMS2	CMS2
GSM972195	CMS3	CMS1	CMS1			CMS2	CMS3
GSM972196	CMS1	CMS1	CMS1	CMS1	CMS1	CMS1	CMS1
GSM972197	CMS3	CMS3	CMS1	CMS3	CMS1	CMS3	CMS1
GSM972198	CMS2	CMS2	CMS2	CMS2	CMS2	CMS2	CMS2
GSM972199	CMS2	CMS2	CMS2	CMS2	CMS2	CMS2	CMS2
GSM972200	CMS2	CMS2	CMS2	CMS2	CMS2	CMS2	CMS2
GSM972201	CMS4	CMS4	CMS4	CMS4		CMS4	CMS4
GSM972202	CMS2	CMS2	CMS2	CMS2	CMS2	CMS2	CMS2
GSM972203	CMS2	CMS2		CMS2	CMS2	CMS2	CMS2
GSM972204		CMS4	CMS4	CMS2	CMS2	CMS2	CMS4
GSM972205	CMS2		CMS2	CMS2	CMS2	CMS2	CMS2
GSM972206	CMS2	CMS4	CMS4	CMS4		CMS4	CMS4
GSM972207		CMS4	CMS4	CMS2	CMS2	CMS2	CMS2
GSM972208	CMS4	CMS4	CMS4	CMS4		CMS4	CMS4
GSM972209	CMS1	CMS1	CMS1	CMS1	CMS1		CMS1
GSM972210		CMS4	CMS4	CMS2	CMS2	CMS4	CMS4
GSM972211	CMS2	CMS4	CMS4	CMS2	CMS2	CMS2	
GSM972212	CMS3	CMS3	CMS3		CMS2	CMS2	CMS2
GSM972213		CMS4	CMS1		CMS1	CMS4	CMS4
GSM972214	CMS1	CMS1	CMS1		CMS1	CMS1	CMS2
GSM972215		CMS1	CMS1	CMS2	CMS2	CMS2	CMS4
GSM972216	CMS2	CMS2	CMS2	CMS2	CMS2	CMS2	CMS2
GSM972217	CMS2	CMS4	CMS4	CMS2	CMS2	CMS2	CMS4
GSM972218	CMS3	CMS3	CMS3	CMS3		CMS3	CMS3
GSM972219	CMS2	CMS2	CMS2	CMS2	CMS2	CMS2	CMS4
GSM972220	CMS1	CMS1	CMS1	CMS1	CMS1		CMS1
GSM972221	CMS1		CMS3			CMS3	CMS3
GSM972222		CMS3	CMS3	CMS2		CMS2	CMS2
GSM972223	CMS1	CMS1	CMS1	CMS1	CMS1		CMS1
GSM972224	CMS1	CMS1	CMS1	CMS1	CMS1	CMS1	CMS1

GSM972225		CMS2	CMS2	CMS2	CMS2	CMS2	CMS2
GSM972226	CMS3	CMS3	CMS3	CMS3		CMS3	CMS3
GSM972227	CMS3	CMS3	CMS3	CMS3		CMS3	CMS2
GSM972228	CMS3	CMS3	CMS3	CMS3			CMS2
GSM972229	CMS1	CMS1	CMS1		CMS1		CMS2
GSM972230	CMS2	CMS2	CMS2	CMS2	CMS2	CMS2	CMS2
GSM972231	CMS4	CMS4	CMS4	CMS4	CMS2	CMS4	CMS2
GSM972232	CMS4	CMS4	CMS4	CMS2	CMS2	CMS2	CMS4
GSM972233	CMS2	CMS2	CMS2	CMS2	CMS2	CMS2	CMS2
GSM972234	CMS4	CMS4	CMS4	CMS4		CMS4	CMS4
GSM972235		CMS2	CMS2	CMS2	CMS2	CMS2	CMS2
GSM972236	CMS2	CMS2	CMS2	CMS2	CMS2	CMS2	CMS2
GSM972237	CMS2	CMS2	CMS2	CMS2	CMS2	CMS2	CMS2
GSM972238		CMS2	CMS2	CMS2	CMS2	CMS2	CMS2
GSM972239			CMS2	CMS2	CMS2	CMS2	CMS2
GSM972240	CMS4		CMS4		CMS1	CMS1	CMS4
GSM972241	CMS1	CMS3	CMS2			CMS2	CMS2
GSM972242		CMS3	CMS3	CMS2	CMS2	CMS2	CMS3
GSM972243	CMS2	CMS2	CMS2	CMS2	CMS2	CMS2	CMS2
GSM972245		CMS2	CMS2	CMS2	CMS2	CMS2	CMS2
GSM972246	CMS2	CMS2	CMS2	CMS2	CMS2	CMS2	CMS2
GSM972247	CMS2	CMS2	CMS2	CMS2	CMS2	CMS2	CMS2
GSM972248	CMS2	CMS2	CMS2	CMS2	CMS2	CMS2	CMS2
GSM972249	CMS2	CMS2	CMS2	CMS2	CMS2	CMS2	CMS2
GSM972250	CMS2	CMS2	CMS2	CMS2	CMS2	CMS2	CMS2
GSM972251	CMS4	CMS4	CMS4			CMS4	CMS4
GSM972252		CMS2	CMS2	CMS2	CMS2	CMS2	CMS4
GSM972253	CMS2	CMS2	CMS2	CMS2	CMS2	CMS2	CMS2
GSM972254	CMS4	CMS4	CMS4	CMS2	CMS2	CMS2	CMS4
GSM972255	CMS4	CMS4	CMS4	CMS2	CMS2	CMS2	CMS4
GSM972256	CMS2	CMS2	CMS2	CMS2	CMS2	CMS2	CMS2
GSM972258	CMS2	CMS2	CMS2	CMS2	CMS2	CMS2	CMS2
GSM972259	CMS3	CMS3	CMS3	CMS3		CMS3	CMS2
GSM972260	CMS2	CMS2	CMS2	CMS2	CMS2	CMS2	CMS2
GSM972261	CMS1	CMS1	CMS1	CMS1	CMS1	CMS1	CMS1
GSM972262	CMS4	CMS4	CMS4	CMS4	CMS4	CMS4	CMS4
GSM972263	CMS1	CMS2	CMS2	CMS2	CMS1	CMS2	CMS1
GSM972264	CMS1	CMS1	CMS1	CMS2		CMS2	CMS1
GSM972265	CMS1	CMS1	CMS1	CMS1	CMS1	CMS1	CMS1
GSM972266		CMS3		CMS2	CMS2	CMS2	CMS2
GSM972267	CMS2	CMS2	CMS2	CMS2	CMS2	CMS2	CMS2
GSM972268	CMS3	CMS3	CMS3	CMS3		CMS3	CMS2
GSM972270	CMS1				CMS1	CMS1	CMS1
GSM972271	CMS2	CMS2	CMS2	CMS2	CMS2	CMS2	CMS2
GSM972272	CMS2	CMS3	CMS2	CMS2	CMS2	CMS2	CMS2
GSM972273	CMS3	CMS3		CMS3		CMS3	CMS2
GSM972274	CMS1	CMS1	CMS1	CMS1	CMS1	CMS1	CMS1
GSM972275		CMS1	CMS1	CMS1	CMS1	CMS1	CMS1
GSM972276	CMS1	CMS1	CMS1	CMS1		CMS3	CMS1
GSM972277	CMS1	CMS3	CMS3	CMS1		CMS3	CMS3
GSM972278	CMS1	CMS1	CMS1	CMS1	CMS1	CMS1	CMS1
GSM972279		CMS1	CMS1		CMS1		CMS1
GSM972280	CMS1	CMS1	CMS1	CMS1	CMS1	CMS1	CMS1
GSM972281	CMS2	CMS2	CMS2	CMS2	CMS2	CMS2	CMS2

GSM972282	CMS1	CMS2	CMS2	CMS2	CMS2	CMS2	CMS2
GSM972283	CMS1	CMS1	CMS1	CMS1	CMS1	CMS1	CMS1
GSM972284	CMS2	CMS2	CMS2	CMS2	CMS2	CMS2	CMS2
GSM972285	CMS1	CMS3	CMS3			CMS2	CMS2
GSM972286	CMS1	CMS1	CMS1	CMS1	CMS1		CMS1
GSM972287	CMS1	CMS1	CMS1	CMS1	CMS1	CMS1	CMS1
GSM972289	CMS2	CMS2	CMS2	CMS2	CMS2	CMS2	CMS2
GSM972290		CMS4	CMS4	CMS2	CMS2	CMS2	CMS4
GSM972291	CMS4	CMS2	CMS2	CMS2	CMS2	CMS2	CMS2
GSM972292	CMS1	CMS1	CMS1		CMS1		CMS1
GSM972293	CMS4		CMS4		CMS1		CMS1
GSM972294	CMS1	CMS1	CMS1	CMS1	CMS1	CMS1	CMS1
GSM972296	CMS3	CMS3	CMS3	CMS3		CMS3	CMS2
GSM972297	CMS4	CMS4	CMS4	CMS2			CMS2
GSM972298	CMS1	CMS3	CMS3	CMS1	CMS1	CMS1	CMS3
GSM972299	CMS2	CMS2	CMS2	CMS2	CMS2	CMS2	CMS2
GSM972301			CMS3		CMS2	CMS2	CMS2
GSM972302	CMS2	CMS2	CMS2	CMS2	CMS2	CMS2	CMS2
GSM972303	CMS2	CMS2	CMS2	CMS2	CMS2	CMS2	CMS2
GSM972304	CMS3	CMS3	CMS3	CMS2		CMS2	CMS2
GSM972305		CMS3	CMS3	CMS3	CMS1	CMS3	CMS3
GSM972306	CMS2	CMS2	CMS2	CMS2	CMS2	CMS2	CMS2
GSM972307	CMS2	CMS2	CMS2	CMS2	CMS2	CMS2	CMS2
GSM972308	CMS1	CMS3	CMS3		CMS1		CMS3
GSM972309	CMS2	CMS2	CMS2	CMS2	CMS2	CMS2	CMS2
GSM972310		CMS2	CMS3		CMS2	CMS2	CMS3
GSM972311	CMS4	CMS2	CMS2	CMS2		CMS2	CMS1
GSM972312	CMS2	CMS2	CMS2	CMS2	CMS2	CMS2	CMS2
GSM972313	CMS2	CMS2	CMS2	CMS2	CMS2	CMS2	CMS2
GSM972314	CMS1	CMS1	CMS1		CMS1	CMS2	CMS1
GSM972315	CMS3	CMS3	CMS3			CMS3	CMS2
GSM972316	CMS1	CMS1		CMS1	CMS1	CMS1	CMS1
GSM972317	CMS1	CMS1	CMS1	CMS1	CMS1	CMS3	CMS1
GSM972318	CMS2	CMS2	CMS2	CMS2	CMS2	CMS2	CMS2
GSM972319	CMS2	CMS2	CMS2	CMS2	CMS2	CMS2	CMS2
GSM972320	CMS2	CMS2	CMS2	CMS2	CMS2	CMS2	CMS2
GSM972321	CMS2	CMS2	CMS2	CMS2	CMS2	CMS2	CMS2
GSM972322	CMS2	CMS2	CMS2	CMS2	CMS2	CMS2	CMS2
GSM972323	CMS2	CMS2	CMS2	CMS2	CMS2	CMS2	CMS2
GSM972324	CMS2	CMS2	CMS2	CMS2	CMS2	CMS2	CMS2
GSM972325	CMS1	CMS3	CMS3	CMS2	CMS1		CMS2
GSM972326	CMS3	CMS3	CMS3	CMS3		CMS3	CMS3
GSM972327		CMS3	CMS3		CMS1		CMS3
GSM972328	CMS2	CMS2	CMS2	CMS2	CMS2	CMS2	CMS2
GSM972329		CMS2	CMS2	CMS2	CMS2	CMS2	CMS2
GSM972330	CMS1	CMS1	CMS1	CMS1	CMS1	CMS1	CMS1
GSM972331	CMS2	CMS2	CMS2	CMS2	CMS2	CMS2	CMS2
GSM972332	CMS1	CMS1	CMS1	CMS1	CMS1	CMS1	CMS1
GSM972333	CMS2	CMS2	CMS2	CMS2	CMS2	CMS2	CMS2
GSM972334	CMS1	CMS1	CMS1	CMS1	CMS1	CMS1	CMS1
GSM972335	CMS2	CMS2	CMS2	CMS2	CMS2	CMS2	CMS2
GSM972336	CMS2	CMS2	CMS2	CMS2	CMS2	CMS2	CMS2
GSM972337	CMS1	CMS1	CMS1	CMS1	CMS1		CMS3
GSM972338	CMS2	CMS2	CMS2	CMS2	CMS2	CMS2	CMS2

GSM972339	CMS2	CMS2	CMS2	CMS2	CMS2	CMS2	CMS2
GSM972340	CMS2	CMS2	CMS2	CMS2	CMS2	CMS2	CMS2
GSM972341	CMS2	CMS2	CMS2	CMS2	CMS2	CMS2	CMS2
GSM972342	CMS2	CMS2	CMS2	CMS2	CMS2	CMS2	CMS2
GSM972343	CMS2	CMS2	CMS2	CMS2	CMS2	CMS2	CMS2
GSM972344	CMS2	CMS2	CMS2	CMS2	CMS2	CMS2	CMS2
GSM972345	CMS4	CMS4	CMS4			CMS4	CMS4
GSM972346	CMS1	CMS3	CMS3	CMS1	CMS1	CMS1	CMS1
GSM972347	CMS2	CMS2	CMS2	CMS2	CMS2	CMS2	CMS2
GSM972348	CMS2	CMS2	CMS2	CMS2	CMS2	CMS2	CMS2
GSM972349	CMS4	CMS4	CMS4	CMS4	CMS4	CMS4	CMS4
GSM972350	CMS3	CMS3	CMS2	CMS2		CMS2	CMS2
GSM972351	CMS2	CMS2	CMS2	CMS2	CMS2	CMS2	CMS2
GSM972352	CMS4	CMS4	CMS4	CMS4		CMS4	CMS4
GSM972353	CMS1	CMS1	CMS1		CMS1		
GSM972354	CMS1	CMS1	CMS1	CMS2	CMS2	CMS2	CMS1
GSM972355		CMS4	CMS4	CMS4		CMS4	CMS4
GSM972356	CMS2	CMS2	CMS2	CMS2	CMS2	CMS2	CMS2
GSM972357		CMS3	CMS2		CMS2	CMS2	CMS2
GSM972358	CMS2	CMS2	CMS2	CMS2	CMS2	CMS2	CMS2
GSM972359	CMS4	CMS4	CMS4	CMS4		CMS4	CMS4
GSM972360	CMS1	CMS1		CMS2	CMS2	CMS2	CMS4
GSM972361	CMS4	CMS4	CMS1	CMS4	CMS4	CMS4	CMS4
GSM972362	CMS2	CMS2	CMS2	CMS2	CMS2	CMS2	CMS2
GSM972363	CMS2	CMS2		CMS2	CMS2	CMS2	CMS2
GSM972364	CMS3	CMS3	CMS3	CMS3		CMS3	CMS3
GSM972365		CMS4	CMS4	CMS4	CMS1	CMS4	CMS4
GSM972366		CMS4	CMS4	CMS4		CMS4	CMS4
GSM972367	CMS4	CMS4	CMS4	CMS4		CMS4	CMS4
GSM972368	CMS2	CMS2	CMS2	CMS2	CMS2	CMS2	CMS2
GSM972369	CMS4	CMS4	CMS4	CMS4		CMS4	CMS4
GSM972370	CMS2	CMS2	CMS2	CMS2	CMS2	CMS2	CMS2
GSM972371	CMS2	CMS2	CMS2	CMS2	CMS2	CMS2	CMS4
GSM972372	CMS2	CMS2	CMS2	CMS2	CMS2	CMS2	CMS4
GSM972373	CMS2	CMS2	CMS2	CMS2	CMS2	CMS2	CMS2
GSM972374	CMS2	CMS2	CMS2	CMS2	CMS2	CMS2	CMS2
GSM972375	CMS4	CMS4	CMS4	CMS4		CMS4	CMS4
GSM972376		CMS4	CMS4	CMS4		CMS4	CMS4
GSM972377	CMS4	CMS4	CMS4	CMS4		CMS4	CMS4
GSM972378	CMS2	CMS4	CMS4		CMS2	CMS2	CMS4
GSM972379	CMS3	CMS3	CMS3	CMS2		CMS2	CMS2
GSM972380	CMS4	CMS4	CMS4	CMS4		CMS4	CMS4
GSM972381	CMS2	CMS2	CMS4		CMS2	CMS2	CMS4
GSM972382	CMS2	CMS2	CMS2	CMS2	CMS2	CMS2	CMS2
GSM972383	CMS2	CMS2	CMS2	CMS2	CMS2	CMS2	CMS2
GSM972384		CMS4	CMS4	CMS2	CMS2	CMS2	CMS4
GSM972385	CMS4	CMS4	CMS4	CMS4		CMS4	CMS4
GSM972386	CMS2	CMS2	CMS2	CMS2	CMS2	CMS2	CMS2
GSM972387	CMS2	CMS2	CMS2	CMS2	CMS2	CMS2	CMS2
GSM972388	CMS2	CMS2	CMS2	CMS2	CMS2	CMS2	CMS2
GSM972389	CMS3	CMS3	CMS3	CMS3		CMS3	CMS3
GSM972390	CMS3	CMS3	CMS1			CMS2	CMS3
GSM972391	CMS1	CMS1	CMS1		CMS1		CMS1
GSM972392	CMS2	CMS2	CMS2	CMS2	CMS2	CMS2	CMS2

GSM972393	CMS2	CMS2	CMS2	CMS2	CMS2	CMS2	CMS2
GSM972394		CMS4	CMS4	CMS4		CMS4	CMS4
GSM972395	CMS2	CMS2	CMS2	CMS2	CMS2	CMS2	CMS2
GSM972396	CMS3	CMS3	CMS3	CMS3		CMS3	CMS3
GSM972397	CMS2	CMS2	CMS2	CMS2	CMS2	CMS2	CMS2
GSM972398	CMS2	CMS2	CMS2	CMS2	CMS2	CMS2	CMS2
GSM972399	CMS2	CMS2	CMS2	CMS2	CMS2	CMS2	CMS2
GSM972400		CMS2	CMS3		CMS2	CMS2	CMS2
GSM972401	CMS4	CMS4	CMS4	CMS4	CMS4	CMS4	CMS4
GSM972402	CMS4	CMS4	CMS4	CMS4		CMS4	CMS4
GSM972403	CMS1	CMS1	CMS1	CMS1	CMS1		CMS1
GSM972404	CMS2	CMS2	CMS2	CMS2	CMS2	CMS2	CMS4
GSM972405		CMS4	CMS4	CMS2	CMS2	CMS2	CMS4
GSM972406	CMS1	CMS1	CMS1		CMS1		CMS1
GSM972407	CMS2	CMS4	CMS4	CMS4		CMS4	CMS4
GSM972408		CMS4	CMS4		CMS1		CMS2
GSM972409	CMS4	CMS4	CMS4	CMS4	CMS4	CMS4	CMS4
GSM972410	CMS4	CMS4	CMS4	CMS4		CMS4	CMS4
GSM972411	CMS2	CMS2	CMS2	CMS2	CMS2	CMS2	CMS2
GSM972412	CMS4	CMS4	CMS4		CMS2	CMS2	CMS4
GSM972413	CMS4	CMS4	CMS4	CMS4	CMS4	CMS4	CMS4
GSM972414	CMS2	CMS2	CMS2	CMS2	CMS2	CMS2	CMS2
GSM972415	CMS2	CMS2	CMS2	CMS2	CMS2	CMS2	CMS2
GSM972416	CMS4	CMS4	CMS4	CMS4		CMS4	CMS4
GSM972417		CMS4	CMS4	CMS2	CMS2	CMS2	CMS4
GSM972418	CMS2	CMS2	CMS2	CMS2	CMS2	CMS2	CMS4
GSM972419	CMS4	CMS4	CMS4	CMS4		CMS4	CMS4
GSM972420		CMS1	CMS1		CMS1	CMS4	CMS1
GSM972421	CMS4	CMS4	CMS4	CMS4		CMS4	CMS4
GSM972422	CMS4	CMS4	CMS4	CMS4		CMS4	CMS4
GSM972423	CMS2	CMS2	CMS2	CMS2	CMS2	CMS2	CMS4
GSM972424	CMS2	CMS2	CMS2	CMS2	CMS2	CMS2	CMS3
GSM972425	CMS4	CMS4	CMS4	CMS4	CMS4	CMS4	CMS4
GSM972426		CMS4	CMS4	CMS4		CMS4	CMS4
GSM972427	CMS2	CMS4	CMS4	CMS4	CMS2	CMS2	CMS4
GSM972428	CMS4	CMS4	CMS4	CMS4		CMS4	CMS4
GSM972429	CMS3	CMS2	CMS2	CMS2	CMS2	CMS2	CMS2
GSM972430	CMS1	CMS1	CMS1		CMS1		CMS2
GSM972431		CMS4	CMS4	CMS4		CMS4	CMS4
GSM972432	CMS2	CMS4	CMS4	CMS4		CMS4	CMS4
GSM972433	CMS4	CMS4	CMS4	CMS4		CMS4	CMS4
GSM972434	CMS2	CMS2	CMS2	CMS2	CMS2	CMS2	CMS2
GSM972435	CMS2	CMS2	CMS2	CMS2	CMS2	CMS2	CMS4
GSM972436	CMS2	CMS4	CMS4	CMS2	CMS2	CMS2	CMS4
GSM972437	CMS4	CMS4	CMS4	CMS4		CMS4	CMS4
GSM972438	CMS4	CMS4	CMS4	CMS4	CMS4	CMS4	CMS4
GSM972439		CMS4	CMS2	CMS2	CMS2	CMS2	CMS4
GSM972440	CMS4	CMS4	CMS4	CMS4		CMS4	CMS4
GSM972441	CMS4	CMS4	CMS4	CMS4		CMS4	CMS4
GSM972442	CMS2	CMS1		CMS2	CMS2	CMS2	CMS2
GSM972443	CMS3	CMS3	CMS2	CMS2		CMS2	CMS2
GSM972444	CMS1	CMS1	CMS1	CMS4	CMS1	CMS1	CMS4
GSM972445	CMS1	CMS1	CMS1		CMS1	CMS2	CMS1
GSM972446		CMS3	CMS2	CMS2		CMS2	CMS1

GSM972447		CMS3	CMS3	CMS1	CMS1		CMS3
GSM972448	CMS1		CMS3	CMS1	CMS1		CMS1
GSM972449				CMS2	CMS2	CMS2	CMS3
GSM972450	CMS2	CMS2	CMS2	CMS2	CMS2	CMS2	CMS2
GSM972451	CMS2	CMS2	CMS2	CMS2	CMS2	CMS2	CMS2
GSM972452	CMS1	CMS1	CMS1	CMS1	CMS1	CMS1	CMS1
GSM972453	CMS3	CMS3	CMS3	CMS2		CMS3	CMS3
GSM972454	CMS2	CMS2	CMS2	CMS2	CMS2	CMS2	CMS2
GSM972455	CMS1	CMS1	CMS1	CMS4	CMS1	CMS4	CMS1
GSM972456	CMS2	CMS2	CMS2	CMS2	CMS2	CMS2	CMS2
GSM972457	CMS3	CMS3	CMS3	CMS3		CMS3	CMS3
GSM972458	CMS1	CMS1	CMS1		CMS1	CMS1	CMS1
GSM972459	CMS3	CMS2	CMS2	CMS2		CMS3	CMS3
GSM972460	CMS2	CMS2	CMS2	CMS2	CMS2	CMS2	CMS2
GSM972461	CMS2	CMS2	CMS2	CMS2	CMS2	CMS2	CMS3
GSM972462	CMS2	CMS2	CMS2	CMS2	CMS2	CMS2	CMS2
GSM972463	CMS3	CMS3	CMS3	CMS3		CMS3	CMS3
GSM972464	CMS4	CMS2	CMS2	CMS2	CMS2	CMS2	CMS2
GSM972465	CMS1			CMS1	CMS1		CMS1
GSM972466	CMS2	CMS2	CMS2	CMS2	CMS2	CMS2	CMS2
GSM972467	CMS4	CMS4	CMS4	CMS4		CMS4	CMS4
GSM972468	CMS2	CMS2	CMS2	CMS2	CMS2	CMS2	CMS2
GSM972469	CMS2	CMS2	CMS2	CMS2	CMS2	CMS2	CMS2
GSM972470	CMS1	CMS1	CMS1	CMS1	CMS1	CMS1	CMS4
GSM972471	CMS1			CMS1		CMS2	CMS2
GSM972472		CMS3	CMS3	CMS3	CMS1	CMS3	CMS3
GSM972473		CMS2	CMS3	CMS2	CMS2	CMS2	CMS2
GSM972474	CMS2	CMS2	CMS2	CMS2	CMS2	CMS2	CMS2
GSM972475	CMS3	CMS3	CMS3	CMS3		CMS3	CMS3
GSM972476	CMS4	CMS4	CMS4	CMS4		CMS4	CMS4
GSM972477		CMS2	CMS2	CMS2		CMS2	CMS2
GSM972478	CMS2	CMS2	CMS2	CMS2	CMS2	CMS2	CMS2
GSM972479	CMS4	CMS4	CMS4	CMS4	CMS4	CMS4	CMS4
GSM972480	CMS3	CMS3	CMS3	CMS2		CMS2	CMS3
GSM972481		CMS2	CMS2	CMS2	CMS2	CMS2	CMS2
GSM972482		CMS3	CMS3		CMS1	CMS3	CMS3
GSM972483	CMS2	CMS2	CMS2	CMS2	CMS2	CMS2	CMS2
GSM972484		CMS2	CMS2	CMS2	CMS2	CMS2	CMS2
GSM972485	CMS2	CMS2	CMS2	CMS2	CMS2	CMS2	CMS2
GSM972486	CMS4	CMS2	CMS2	CMS2	CMS2	CMS2	CMS2
GSM972487	CMS3	CMS3	CMS3	CMS3		CMS3	CMS3
GSM972488	CMS3	CMS3	CMS3	CMS3		CMS3	CMS3
GSM972489	CMS3	CMS3	CMS3	CMS3		CMS3	CMS3
GSM972490	CMS2	CMS2	CMS2	CMS2	CMS2	CMS2	CMS2
GSM972491	CMS2	CMS2	CMS2	CMS2	CMS2	CMS2	CMS2
GSM972492	CMS2	CMS2	CMS2	CMS2	CMS2	CMS2	CMS2
GSM972493	CMS4	CMS2	CMS2	CMS2	CMS2	CMS2	CMS2
GSM972494	CMS1	CMS3	CMS3	CMS2	CMS2	CMS2	CMS3
GSM972495	CMS2	CMS2	CMS2	CMS2	CMS2	CMS2	CMS2
GSM972496	CMS3	CMS3	CMS2	CMS2	CMS2	CMS2	CMS2
GSM972497	CMS2	CMS2	CMS2	CMS2	CMS2	CMS2	CMS2
GSM972498	CMS2	CMS2	CMS2	CMS2	CMS2	CMS2	CMS2
GSM972499	CMS1	CMS1	CMS1	CMS1	CMS1	CMS1	CMS1
GSM972501	CMS2	CMS2	CMS2	CMS2	CMS2	CMS2	CMS2

GSM972502	CMS3	CMS3	CMS3		CMS3		CMS3			CMS2
GSM972503	CMS4	CMS4	CMS4		CMS2	CMS2	CMS4			CMS4
GSM972504	CMS4	CMS4	CMS4			CMS2				CMS4
GSM972505	CMS2	CMS2	CMS2		CMS2	CMS2	CMS2			CMS2
GSM972506	CMS2	CMS2	CMS2		CMS2	CMS2	CMS2			CMS2
GSM972507	CMS1		CMS3		CMS3					CMS3
GSM972508	CMS2	CMS2	CMS2		CMS2	CMS2	CMS2			CMS2
GSM972509	CMS2	CMS2	CMS2		CMS2	CMS2	CMS2			CMS2
GSM972510	CMS3	CMS3	CMS3		CMS3		CMS3			CMS2
GSM972511	CMS2	CMS2	CMS2		CMS2	CMS2	CMS2			CMS2
GSM972512	CMS3	CMS3	CMS3		CMS3		CMS3			CMS3
GSM972513	CMS2	CMS2	CMS2		CMS2	CMS2	CMS2			CMS2
GSM972514	CMS2	CMS2	CMS2		CMS2	CMS2	CMS2			CMS2
GSM972515	CMS2	CMS2	CMS2		CMS2	CMS2	CMS2			CMS2
GSM972516	CMS2	CMS3	CMS2		CMS2		CMS2			CMS2
GSM972517		CMS2	CMS2		CMS2	CMS2	CMS2			CMS2
GSM972518		CMS4	CMS4		CMS2	CMS2	CMS2			CMS2
GSM972519		CMS4	CMS4		CMS2	CMS2	CMS2			CMS2
GSM972520		CMS2	CMS2		CMS2	CMS2	CMS2			CMS2
GSM972521	CMS4	CMS4	CMS4		CMS4		CMS4			CMS4
GSM972522			CMS4			CMS1				CMS1
Total classifiable samples	440	531	539		477	398	507			554

Table S3: CIT classification results

4 Table S4 CIT (GSE39582) clinical relevance

	Neg	Pos	Fisher's Exact Test	Accuracy
MMR(MSI/MSS)				
CMS1	43	22	5.13×10^{-26}	0.941
Non-CMS1	32	414		
CIMP				
CMS1	25	42	1.51×10^{-18}	0.941
Non-CMS1	372	49		
CIN				
CMS2	12	169	1.72×10^{-13}	0.874
Non-CMS2	97	180		
TP53				
CMS2&4	85	155	5.09×10^{-9}	0.837
CMS1&3	76	34		
BRAF				
CMS1	40	30	5.52×10^{-16}	0.941
Non-CMS1	413	21		
KRAS				
CMS3	25	57	3.05×10^{-9}	0.941
Non-CMS3	300	155		

Table S4.1: DeepCC (531 out of 557)

	Neg	Pos	Fisher's Exact Test	Accuracy
MMR(MSI/MSS)				
CMS1	43	20	6.59×10^{-27}	0.937
Non-CMS1	32	416		
CIMP				
CMS1	22	45	5.07×10^{-22}	0.937
Non-CMS1	375	46		
CIN				
CMS2	19	168	4.22×10^{-9}	0.873
Non-CMS2	90	181		
TP53				
CMS2&4	95	153	1.07×10^{-5}	0.840
CMS1&3	66	36		
BRAF				
CMS1	38	32	2.09×10^{-18}	0.937
Non-CMS1	415	19		
KRAS				
CMS3	21	53	2.54×10^{-9}	0.930
Non-CMS3	304	159		

Table S4.2: DeepCC SSP (539 out of 557)

	Neg	Pos	Fisher's Exact Test	Accuracy
MMR(MSI/MSS)				
CMS1	33	7	2.12×10^{-24}	0.928
Non-CMS1	42	429		
CIMP				
CMS1	6	36	1.62×10^{-23}	0.928
Non-CMS1	391	55		
CIN				
CMS2	27	205	4.32×10^{-10}	0.833
Non-CMS2	82	144		
TP53				
CMS2&4	98	153	4.57×10^{-5}	0.803
CMS1&3	63	36		
BRAF				
CMS1	16	27	6.5×10^{-20}	0.928
Non-CMS1	437	24		
KRAS				
CMS3	9	30	1.43×10^{-6}	0.944
Non-CMS3	316	182		

Table S4.3: Random Forest (477 out of 557)

	Neg	Pos	Fisher's Exact Test	Accuracy
MMR(MSI/MSS)				
CMS1	49	28	1.96×10^{-29}	0.937
Non-CMS1	26	408		
CIMP				
CMS1	28	55	1.02×10^{-27}	0.937
Non-CMS1	369	36		
CIN				
CMS2	16	207	4.37×10^{-17}	0.865
Non-CMS2	93	142		
TP53				
CMS2&4	74	132	7.35×10^{-6}	0.776
CMS1&3	87	57		
BRAF				
CMS1	46	40	2.65×10^{-25}	0.937
Non-CMS1	407	11		
KRAS				
CMS3	0	0	1	0.885
Non-CMS3	325	212		

Table S4.4: SVM (398 out of 557)

	Neg	Pos	Fisher's Exact Test	Accuracy
MMR(MSI/MSS)				
CMS1	23	5	1.48×10^{-16}	0.896
Non-CMS1	52	431		
CIMP				
CMS1	6	22	1.37×10^{-12}	0.896
Non-CMS1	391	69		
CIN				
CMS2	32	216	3.11×10^{-9}	0.819
Non-CMS2	77	133		
TP53				
CMS2&4	110	168	2.5×10^{-6}	0.768
CMS1&3	51	21		
BRAF				
CMS1	14	15	4.22×10^{-9}	0.896
Non-CMS1	439	36		
KRAS				
CMS3	12	34	9.58×10^{-7}	0.943
Non-CMS3	313	178		

Table S4.5: GBM (507 out of 557)

	Neg	Pos	Fisher's Exact Test	Accuracy
MMR(MSI/MSS)				
CMS1	43	17	2.35×10^{-28}	0.925
Non-CMS1	32	419		
CIMP				
CMS1	20	43	2.54×10^{-21}	0.925
Non-CMS1	377	48		
CIN				
CMS2	30	184	3.48×10^{-6}	0.797
Non-CMS2	79	165		
TP53				
CMS2&4	104	162	4.98×10^{-6}	0.768
CMS1&3	57	27		
BRAF				
CMS1	34	32	2×10^{-19}	0.925
Non-CMS1	419	19		
KRAS				
CMS3	20	41	5.14×10^{-6}	0.905
Non-CMS3	305	171		

Table S4.6: Logistic regression model (554 out of 557)

5 Table S5 CRC top correlated biological features

Top correlated biological feature	Correlation
KANG AR TARGETS DN	0.703
JECHLINGER EPITHELIAL TO MESENCHYMAL TRANSITION UP	0.700
SEMBA FHIT TARGETS UP	0.698
LU TUMOR VASCULATURE UP	0.686
MIKKELSEN DEDIFFERENTIATED STATE DN	0.676
SAGIV CD24 TARGETS DN	0.673
chr7p	0.673
PETROVA PROX1 TARGETS DN	0.666
IGLESIAS E2F TARGETS UP	0.658
GILDEA METASTASIS	0.655
REACTOME MITOCHONDRIAL TRNA AMINOACYLATION	-0.380
MICROBODY	-0.399
PEROXISOME	-0.399
ORGANELLE INNER MEMBRANE	-0.405
WALLACE PROSTATE CANCER UP	-0.419
PEROXISOME ORGANIZATION AND BIOGENESIS	-0.422
REACTOME PEROXISOMAL LIPID METABOLISM	-0.441
MANNOSYLTRANSFERASE ACTIVITY	-0.445
KEGG PEROXISOME	-0.451
WATANABE COLON CANCER MSI VS MSS DN	-0.509

Table S5.1: Deep biological feature 1

Top correlated biological feature	Correlation
WATANABE COLON CANCER MSI VS MSS UP	0.615
FOURNIER ACINAR DEVELOPMENT LATE DN	0.509
REACTOME ERKS ARE INACTIVATED	0.495
LIU IL13 MEMORY MODEL DN	0.482
JOHANSSON BRAIN CANCER EARLY VS LATE UP	0.466
PID RANBP2 PATHWAY	0.459
INAMURA LUNG CANCER SCC SUBTYPES UP	0.449
BIOCARTA ETC PATHWAY	0.443
BIOCARTA EPONFKB PATHWAY	0.435
SESTO RESPONSE TO UV C7	0.435
AMINE TRANSMEMBRANE TRANSPORTER ACTIVITY	-0.570
WATANABE COLON CANCER MSI VS MSS DN	-0.570
SCHLESINGER METHYLATED IN COLON CANCER	-0.574
BOYAULT LIVER CANCER SUBCLASS G3 DN	-0.590
KOINUMA COLON CANCER MSI DN	-0.604
DIERICK SEROTONIN FUNCTION GENES	-0.632
chr20q11	-0.636
BODY FLUID SECRETION	-0.638
chr20q13	-0.646
NIKOLSKY BREAST CANCER 20Q11 AMPLICON	-0.673

Table S5.2: Deep biological feature 2

Top correlated biological feature	Correlation
REACTOME MITOCHONDRIAL TRNA AMINOACYLATION	0.513
IRITANI MAD1 TARGETS DN	0.512
REACTOME MITOCHONDRIAL PROTEIN IMPORT	0.507
GNF2 DAP3	0.473
ORGANELLE INNER MEMBRANE	0.472
GCM ACTG1	0.457
RRNA METABOLIC PROCESS	0.456
MORF UBE2I	0.451
KEGG BASE EXCISION REPAIR	0.448
BASE EXCISION REPAIR	0.447
SCHUETZ BREAST CANCER DUCTAL INVASIVE UP	-0.746
COWLING MYCN TARGETS	-0.747
MODULE 12	-0.750
MODULE 1	-0.751
MIKKELSEN DEDIFFERENTIATED STATE DN	-0.752
LIM MAMMARY STEM CELL UP	-0.754
SERVITJA ISLET HNF1A TARGETS UP	-0.754
HALLMARK EPITHELIAL MESENCHYMAL TRANSITION	-0.760
VECCHI GASTRIC CANCER ADVANCED VS EARLY UP	-0.761
MODULE 47	-0.770

Table S5.3: Deep biological feature 3

Top correlated biological feature	Correlation
WATANABE COLON CANCER MSI VS MSS DN	0.581
MANNOSYLTRANSFERASE ACTIVITY	0.445
KEGG PEROXISOME	0.431
NIKOLSKY BREAST CANCER 20Q11 AMPLICON	0.425
PEROXISOME ORGANIZATION AND BIOGENESIS	0.425
REACTOME MITOCHONDRIAL TRNA AMINOACYLATION	0.423
IRITANI MAD1 TARGETS DN	0.421
KOINUMA COLON CANCER MSI DN	0.416
MICROBODY	0.411
PEROXISOME	0.411
GSE360 HIGH DOSE B MALAYI VS M TUBERCULOSIS DC DN	-0.689
HOSHIDA LIVER CANCER SUBCLASS S1	-0.693
MARKEY RB1 ACUTE LOF UP	-0.695
ZHANG ANTIVIRAL RESPONSE TO RIBAVIRIN DN	-0.695
PETROVA PROX1 TARGETS DN	-0.697
SEMBA FHIT TARGETS UP	-0.698
CALMODULIN BINDING	-0.706
MARSON FOXP3 TARGETS UP	-0.711
SAGIV CD24 TARGETS DN	-0.713
KOINUMA COLON CANCER MSI UP	-0.716

Table S5.4: Deep biological feature 4

Top correlated biological feature	Correlation
GRADE COLON AND RECTAL CANCER UP	0.468
NIKOLSKY BREAST CANCER 20Q11 AMPLICON	0.446
REACTOME METABOLISM OF VITAMINS AND COFACTORS	0.434
KOINUMA COLON CANCER MSI DN	0.427
WATANABE COLON CANCER MSI VS MSS DN	0.417
DIERICK SEROTONIN FUNCTION GENES	0.416
CAIRO HEPATOBLASTOMA CLASSES UP	0.405
chr20q11	0.403
PID E2F PATHWAY	0.400
ELVIDGE HIF1A AND HIF2A TARGETS UP	0.399
N ACETYLGLUCOSAMINE METABOLIC PROCESS	-0.332
HINATA NFKB TARGETS KERATINOCYTE DN	-0.333
REACTOME ACYL CHAIN REMODELLING OF PE	-0.338
REACTOME ERKS ARE INACTIVATED	-0.344
chr11q11	-0.345
REACTOME ACYL CHAIN REMODELLING OF PG	-0.351
LIU IL13 MEMORY MODEL DN	-0.352
PID HNF3A PATHWAY	-0.352
TIAN BHLHA15 TARGETS	-0.366
WATANABE COLON CANCER MSI VS MSS UP	-0.477

Table S5.5: Deep biological feature 5

Top correlated biological feature	Correlation
NIKOLSKY BREAST CANCER 20Q11 AMPLICON	0.635
chr20q13	0.628
chr20q11	0.591
BODY FLUID SECRETION	0.582
SCHLESINGER METHYLATED IN COLON CANCER	0.581
BOYAULT LIVER CANCER SUBCLASS G3 DN	0.562
ASTON MAJOR DEPRESSIVE DISORDER UP	0.558
REGULATION OF NEUROTRANSMITTER LEVELS	0.550
DIERICK SEROTONIN FUNCTION GENES	0.550
MODULE 201	0.544
LIU IL13 MEMORY MODEL DN	-0.465
JOHANSSON BRAIN CANCER EARLY VS LATE UP	-0.465
BIOCARTA ETC PATHWAY	-0.469
VECCHI GASTRIC CANCER ADVANCED VS EARLY DN	-0.469
GREENBAUM E2A TARGETS UP	-0.474
WAMUNYOKOLI OVARIAN CANCER LMP UP	-0.476
BIOCARTA MITOCHONDRIA PATHWAY	-0.485
FOURNIER ACINAR DEVELOPMENT LATE DN	-0.487
WAMUNYOKOLI OVARIAN CANCER GRADES 1 2 UP	-0.520
WATANABE COLON CANCER MSI VS MSS UP	-0.565

Table S5.6: Deep biological feature 6

Top correlated biological feature	Correlation
WATANABE COLON CANCER MSI VS MSS UP	0.758
KOINUMA COLON CANCER MSI UP	0.543
LIU IL13 MEMORY MODEL DN	0.540
REACTOME ERKS ARE INACTIVATED	0.534
BIOCARTA EPONFKB PATHWAY	0.528
chr18q21	0.501
HUPER BREAST BASAL VS LUMINAL DN	0.465
KIM LRRC3B TARGETS	0.460
N ACETYLGLUCOSAMINE METABOLIC PROCESS	0.458
RICKMAN METASTASIS DN	0.457
chr13q34	-0.517
REACTOME ORGANIC CATION ANION ZWITTERION TRANSPORT	-0.548
REACTOME GAMMA CARBOXYLATION TRANSPORT AND AMINO TERMINAL CLEAVAGE OF PROTEINS	-0.574
BODY FLUID SECRETION	-0.577
chr20q13	-0.604
DIERICK SEROTONIN FUNCTION GENES	-0.651
chr20q11	-0.665
KOINUMA COLON CANCER MSI DN	-0.709
NIKOLSKY BREAST CANCER 20Q11 AMPLICON	-0.722
WATANABE COLON CANCER MSI VS MSS DN	-0.725

Table S5.7: Deep biological feature 7

Top correlated biological feature	Correlation
WATANABE COLON CANCER MSI VS MSS UP	0.465
TIAN BHLHA15 TARGETS	0.422
VECCHI GASTRIC CANCER ADVANCED VS EARLY DN	0.416
PID HNF3A PATHWAY	0.415
LIU IL13 MEMORY MODEL DN	0.412
UDP GLYCOSYLTRANSFERASE ACTIVITY	0.410
ACETYLGALACTOSAMINYLTRANSFERASE ACTIVITY	0.403
SMID BREAST CANCER RELAPSE IN BONE UP	0.399
GALACTOSYLTRANSFERASE ACTIVITY	0.397
MODULE 294	0.391
REACTOME GAMMA CARBOXYLATION TRANSPORT AND AMINO TERMINAL CLEAVAGE OF PROTEINS	-0.328
GSE10239 KLRG1INT VS KLRG1HIGH EFF CD8 TCELL UP	-0.335
GRADE COLON AND RECTAL CANCER UP	-0.342
chr13q34	-0.364
NIKOLSKY BREAST CANCER 20Q12 Q13 AMPLICON	-0.368
KOINUMA COLON CANCER MSI DN	-0.368
DIERICK SEROTONIN FUNCTION GENES	-0.376
chr20q13	-0.388
chr20q11	-0.404
NIKOLSKY BREAST CANCER 20Q11 AMPLICON	-0.445

Table S5.8: Deep biological feature 8

Top correlated biological feature	Correlation
WATANABE COLON CANCER MSI VS MSS UP	0.759
LIU IL13 MEMORY MODEL DN	0.584
REACTOME ERKS ARE INACTIVATED	0.562
BIOCARTA EPONFKB PATHWAY	0.550
KOINUMA COLON CANCER MSI UP	0.537
chr18q21	0.525
MYLLYKANGAS AMPLIFICATION HOT SPOT 18	0.475
SCHAVOLT TARGETS OF TP53 AND TP63	0.475
MODULE 294	0.469
N ACETYLGLUCOSAMINE METABOLIC PROCESS	0.466
chr13q34	-0.522
REACTOME ORGANIC CATION ANION ZWITTERION TRANSPORT	-0.552
REACTOME GAMMA CARBOXYLATION TRANSPORT AND AMINO TERMINAL CLEAVAGE OF PROTEINS	-0.578
BODY FLUID SECRETION	-0.591
chr20q13	-0.618
DIERICK SEROTONIN FUNCTION GENES	-0.650
chr20q11	-0.682
KOINUMA COLON CANCER MSI DN	-0.686
WATANABE COLON CANCER MSI VS MSS DN	-0.687
NIKOLSKY BREAST CANCER 20Q11 AMPLICON	-0.733

Table S5.9: Deep biological feature 9

Top correlated biological feature	Correlation
WATANABE COLON CANCER MSI VS MSS DN	0.492
KEGG PEROXISOME	0.452
REACTOME PEROXISOMAL LIPID METABOLISM	0.451
MANNOSYLTRANSFERASE ACTIVITY	0.445
PEROXISOME ORGANIZATION AND BIOGENESIS	0.426
ORGANELLE INNER MEMBRANE	0.426
WALLACE PROSTATE CANCER UP	0.422
REACTOME MITOCHONDRIAL TRNA AMINOACYLATION	0.415
MICROBODY	0.396
PEROXISOME	0.396
GILDEA METASTASIS	-0.670
VECCHI GASTRIC CANCER ADVANCED VS EARLY UP	-0.672
PETROVA PROX1 TARGETS DN	-0.673
SAGIV CD24 TARGETS DN	-0.679
MIKKELSEN DEDIFFERENTIATED STATE DN	-0.690
chr7p	-0.692
SEMBA FHIT TARGETS UP	-0.694
LU TUMOR VASCULATURE UP	-0.694
JECHLINGER EPITHELIAL TO MESENCHYMAL TRANSITION UP	-0.713
KANG AR TARGETS DN	-0.715

Table S5.10: Deep biological feature 10

6 Table S6 Summary of breast cancer data sets

Data Set	Platform	Total	with PAM50*	Source
TRANSBIG	Affymetrix HG133A	198	198	breastCancerTRANSBIG**(GSE7390)
UPP	Affymetrix HG133AB	251	190	breastCancerUPP**(GSE3494)
UNT	Affymetrix HG133AB	137	92	breastCancerUNT**(GSE2990 and GSE6532)
NKI	Agilent	337	337	breastCancerNKI**
TCGA	RNA-Seq	1100	517	TCGA BRCA
MAQC II BR	Affymetrix HG U133A	230		GSE20194
Total		2253	1334	

Table S6: Summary of breast cancer data sets

*Reference:

1. Cancer Genome Atlas Network. Comprehensive molecular portraits of human breast tumours. Nature 490, 61–70 (2012).

2. Haibe-Kains, B. et al. A three-gene model to robustly identify breast cancer molecular subtypes. J. Natl. Cancer Inst. 104, 311–325 (2012).

**data package available on Bioconductor

7 Table S7 Breast cancer top correlated biological features

Top correlated biological feature	Correlation
SOTIRIOU BREAST CANCER GRADE 1 VS 3 DN	0.653
POOLA INVASIVE BREAST CANCER DN	0.635
SMID BREAST CANCER LUMINAL A UP	0.633
VANTVEER BREAST CANCER METASTASIS UP	0.600
SMID BREAST CANCER BASAL DN	0.583
ONDER CDH1 TARGETS 3 UP	0.573
KASLER HDAC7 TARGETS 2 DN	0.573
RAF UP.V1 DN	0.568
REACTOME CASPASE MEDIATED CLEAVAGE OF CYTOSKELETAL PROTEINS	0.566
DOANE BREAST CANCER ESR1 UP	0.565
GNF2 CDC2	-0.790
GNF2 HMMR	-0.791
GNF2 CKS1B	-0.791
GNF2 H2AFX	-0.791
GNF2 RRM2	-0.791
GNF2 MKI67	-0.792
SOTIRIOU BREAST CANCER GRADE 1 VS 3 UP	-0.793
GNF2 CDC20	-0.793
GNF2 CENPE	-0.793
GNF2 BUB1	-0.797

Table S7.1: Deep biological feature 1

Top correlated biological feature	Correlation
PUJANA BREAST CANCER WITH BRCA1 MUTATED UP	0.458
MORF ESPL1	0.439
GNF2 MSH2	0.438
MORF PRKDC	0.436
MORF BUB1B	0.435
KALMA E2F1 TARGETS	0.434
PUJANA BRCA CENTERED NETWORK	0.433
GNF2 ANP32B	0.431
GNF2 RFC4	0.431
GNF2 MCM5	0.430
NELSON RESPONSE TO ANDROGEN UP	-0.434
FARMER BREAST CANCER APOCRINE VS BASAL	-0.444
LUI THYROID CANCER CLUSTER 5	-0.444
VECCHI GASTRIC CANCER ADVANCED VS EARLY DN	-0.451
FRASOR RESPONSE TO ESTRADIOL DN	-0.456
WANG BARRETTS ESOPHAGUS AND ESOPHAGUS CANCER UP	-0.468
SMID BREAST CANCER RELAPSE IN LUNG DN	-0.474
DOANE RESPONSE TO ANDROGEN UP	-0.476
SMID BREAST CANCER ERBB2 UP	-0.494
DOANE BREAST CANCER CLASSES UP	-0.525

Table S7.2: Deep biological feature 2

Top correlated biological feature	Correlation
YANG BREAST CANCER ESR1 UP	0.852
VANTVEER BREAST CANCER ESR1 UP	0.832
DOANE BREAST CANCER ESR1 UP	0.812
SMID BREAST CANCER LUMINAL B UP	0.806
SMID BREAST CANCER RELAPSE IN BRAIN DN	0.772
YANG BREAST CANCER ESR1 LASER UP	0.762
SMID BREAST CANCER BASAL DN	0.758
YANG BREAST CANCER ESR1 BULK UP	0.755
HALLMARK ESTROGEN RESPONSE EARLY	0.754
SMID BREAST CANCER RELAPSE IN BONE UP	0.749
RAHMAN TP53 TARGETS PHOSPHORYLATED	-0.599
YANG BREAST CANCER ESR1 LASER DN	-0.604
SMID BREAST CANCER BASAL UP	-0.617
VANTVEER BREAST CANCER METASTASIS DN	-0.636
BENPORATH ES CORE NINE	-0.643
YANG BREAST CANCER ESR1 DN	-0.652
YANG BREAST CANCER ESR1 BULK DN	-0.662
SMID BREAST CANCER LUMINAL A DN	-0.667
BENPORATH ES CORE NINE CORRELATED	-0.680
VANTVEER BREAST CANCER ESR1 DN	-0.732

Table S7.3: Deep biological feature 3

Top correlated biological feature	Correlation
PID ATM PATHWAY	0.481
WAKASUGI HAVE ZNF143 BINDING SITES	0.470
TOYOTA TARGETS OF MIR34B AND MIR34C	0.469
PID BARD1 PATHWAY	0.466
REACTOME FANCONI ANEMIA PATHWAY	0.466
RIZ ERYTHROID DIFFERENTIATION	0.466
PUJANA XPRSS INT NETWORK	0.465
PUJANA BRCA2 PCC NETWORK	0.464
PUJANA BRCA CENTERED NETWORK	0.463
SPINDLE MICROTUBULE	0.462
LUI THYROID CANCER CLUSTER 5	-0.315
KEGG STEROID HORMONE BIOSYNTHESIS	-0.323
FRASOR RESPONSE TO ESTRADIOL DN	-0.328
VECCHI GASTRIC CANCER ADVANCED VS EARLY DN	-0.330
HOFFMAN CLOCK TARGETS UP	-0.332
WANG BARRETTS ESOPHAGUS AND ESOPHAGUS CANCER UP	-0.335
SMID BREAST CANCER RELAPSE IN LUNG DN	-0.339
DOANE RESPONSE TO ANDROGEN UP	-0.340
DOANE BREAST CANCER CLASSES UP	-0.384
SMID BREAST CANCER ERBB2 UP	-0.429

Table S7.4: Deep biological feature 4

Top correlated biological feature	Correlation
TURASHVILI BREAST LOBULAR CARCINOMA VS DUCTAL NORMAL DN	0.578
TURASHVILI BREAST DUCTAL CARCINOMA VS DUCTAL NORMAL DN	0.555
TURASHVILI BREAST LOBULAR CARCINOMA VS LOBULAR NORMAL UP	0.549
TURASHVILI BREAST DUCTAL CARCINOMA VS LOBULAR NORMAL DN	0.531
SMID BREAST CANCER ERBB2 DN	0.524
STRUCTURAL CONSTITUENT OF CYTOSKELETON	0.515
LIM MAMMARY LUMINAL MATURE DN	0.497
DORN ADENOVIRUS INFECTION 24HR UP	0.496
COLIN PILOCYTIC ASTROCYTOMA VS GLIOBLASTOMA UP	0.485
STRUCTURAL MOLECULE ACTIVITY	0.474
GNF2 CKS2	-0.473
GNF2 RRM2	-0.473
GNF2 BUB1B	-0.473
GNF2 CENPF	-0.475
GNF2 CENPE	-0.475
CROSBY E2F4 TARGETS	-0.478
MORF FEN1	-0.479
WHITFIELD CELL CYCLE LITERATURE	-0.481
GNF2 MKI67	-0.481
KANG DOXORUBICIN RESISTANCE UP	-0.482

Table S7.5: Deep biological feature 5

Top correlated biological feature	Correlation
GNF2 MKI67	0.573
CROSBY E2F4 TARGETS	0.570
GNF2 CENPE	0.569
GNF2 RRM2	0.566
GNF2 CENPF	0.566
WHITFIELD CELL CYCLE LITERATURE	0.565
GNF2 CDC20	0.565
KANG DOXORUBICIN RESISTANCE UP	0.564
GNF2 BUB1B	0.564
MONTERO THYROID CANCER POOR SURVIVAL UP	0.563
MODULE 387	-0.431
DORN ADENOVIRUS INFECTION 24HR UP	-0.433
CHANDRAN METASTASIS TOP50 DN	-0.436
AMINO ACID DERIVATIVE BIOSYNTHETIC PROCESS	-0.436
STRUCTURAL CONSTITUENT OF CYTOSKELETON	-0.470
SMID BREAST CANCER ERBB2 DN	-0.480
TURASHVILI BREAST DUCTAL CARCINOMA VS LOBULAR NORMAL DN	-0.483
TURASHVILI BREAST DUCTAL CARCINOMA VS DUCTAL NORMAL DN	-0.490
TURASHVILI BREAST LOBULAR CARCINOMA VS LOBULAR NORMAL UP	-0.544
TURASHVILI BREAST LOBULAR CARCINOMA VS DUCTAL NORMAL DN	-0.548

Table S7.6: Deep biological feature 6

Top correlated biological feature	Correlation
VANTVEER BREAST CANCER ESR1 DN	0.713
BENPORATH ES CORE NINE CORRELATED	0.654
YANG BREAST CANCER ESR1 DN	0.645
SMID BREAST CANCER LUMINAL A DN	0.643
YANG BREAST CANCER ESR1 BULK DN	0.638
BENPORATH ES CORE NINE	0.609
VANTVEER BREAST CANCER METASTASIS DN	0.605
SMID BREAST CANCER BASAL UP	0.596
YANG BREAST CANCER ESR1 LASER DN	0.586
RAHMAN TP53 TARGETS PHOSPHORYLATED	0.571
SMID BREAST CANCER BASAL DN	-0.729
SMID BREAST CANCER RELAPSE IN BONE UP	-0.731
YANG BREAST CANCER ESR1 BULK UP	-0.738
YANG BREAST CANCER ESR1 LASER UP	-0.738
HALLMARK ESTROGEN RESPONSE EARLY	-0.742
SMID BREAST CANCER RELAPSE IN BRAIN DN	-0.751
SMID BREAST CANCER LUMINAL B UP	-0.794
DOANE BREAST CANCER ESR1 UP	-0.795
VANTVEER BREAST CANCER ESR1 UP	-0.816
YANG BREAST CANCER ESR1 UP	-0.835

Table S7.7: Deep biological feature 7

Top correlated biological feature	Correlation
YANG BREAST CANCER ESR1 UP	0.877
VANTVEER BREAST CANCER ESR1 UP	0.866
DOANE BREAST CANCER ESR1 UP	0.847
SMID BREAST CANCER LUMINAL B UP	0.835
SMID BREAST CANCER RELAPSE IN BRAIN DN	0.803
SMID BREAST CANCER BASAL DN	0.795
YANG BREAST CANCER ESR1 LASER UP	0.790
HALLMARK ESTROGEN RESPONSE EARLY	0.789
VANTVEER BREAST CANCER METASTASIS UP	0.787
YANG BREAST CANCER ESR1 BULK UP	0.783
RAHMAN TP53 TARGETS PHOSPHORYLATED	-0.621
NADERI BREAST CANCER PROGNOSIS UP	-0.622
BENPORATH PROLIFERATION	-0.640
BENPORATH ES CORE NINE	-0.647
YANG BREAST CANCER ESR1 DN	-0.660
VANTVEER BREAST CANCER METASTASIS DN	-0.672
YANG BREAST CANCER ESR1 BULK DN	-0.678
SMID BREAST CANCER LUMINAL A DN	-0.694
BENPORATH ES CORE NINE CORRELATED	-0.702
VANTVEER BREAST CANCER ESR1 DN	-0.757

Table S7.8: Deep biological feature 8

Top correlated biological feature	Correlation
SMID BREAST CANCER RELAPSE IN BRAIN DN	0.807
LIEN BREAST CARCINOMA METAPLASTIC VS DUCTAL DN	0.797
CHARAFE BREAST CANCER LUMINAL VS BASAL UP	0.795
YANG BREAST CANCER ESR1 LASER UP	0.791
YANG BREAST CANCER ESR1 UP	0.788
DOANE BREAST CANCER ESR1 UP	0.784
YANG BREAST CANCER ESR1 BULK UP	0.775
SMID BREAST CANCER BASAL DN	0.774
VANTVEER BREAST CANCER ESR1 UP	0.773
CHARAFE BREAST CANCER LUMINAL VS MESENCHYMAL UP	0.768
DOANE BREAST CANCER ESR1 DN	-0.675
LANDEMAINE LUNG METASTASIS	-0.677
YANG BREAST CANCER ESR1 DN	-0.679
YANG BREAST CANCER ESR1 LASER DN	-0.682
BENPORATH ES CORE NINE	-0.689
SMID BREAST CANCER RELAPSE IN BRAIN UP	-0.699
SMID BREAST CANCER BASAL UP	-0.706
YANG BREAST CANCER ESR1 BULK DN	-0.714
VANTVEER BREAST CANCER ESR1 DN	-0.757
BENPORATH ES CORE NINE CORRELATED	-0.783

Table S7.9: Deep biological feature 9

Top correlated biological feature	Correlation
VANTVEER BREAST CANCER ESR1 DN	0.742
BENPORATH ES CORE NINE CORRELATED	0.697
SMID BREAST CANCER LUMINAL A DN	0.677
YANG BREAST CANCER ESR1 DN	0.667
YANG BREAST CANCER ESR1 BULK DN	0.667
BENPORATH ES CORE NINE	0.650
VANTVEER BREAST CANCER METASTASIS DN	0.645
SMID BREAST CANCER BASAL UP	0.625
BENPORATH PROLIFERATION	0.616
YANG BREAST CANCER ESR1 LASER DN	0.614
HALLMARK ESTROGEN RESPONSE EARLY	-0.763
SMID BREAST CANCER RELAPSE IN BONE UP	-0.767
YANG BREAST CANCER ESR1 BULK UP	-0.768
YANG BREAST CANCER ESR1 LASER UP	-0.775
SMID BREAST CANCER BASAL DN	-0.775
SMID BREAST CANCER RELAPSE IN BRAIN DN	-0.788
SMID BREAST CANCER LUMINAL B UP	-0.817
DOANE BREAST CANCER ESR1 UP	-0.828
VANTVEER BREAST CANCER ESR1 UP	-0.843
YANG BREAST CANCER ESR1 UP	-0.865

Table S7.10: Deep biological feature 10

8 Table S8 Breast cancer classification results

Sample Name	Data Set	Series	ID	PAM50	DeepCC
VDXGUYU 4002	TRANSBIG	VDXGUYU	4002	Her2	Her2
VDXGUYU 4008	TRANSBIG	VDXGUYU	4008	LumB	LumB
VDXGUYU 4011	TRANSBIG	VDXGUYU	4011	Basal	Basal
VDXGUYU 4014	TRANSBIG	VDXGUYU	4014	LumB	LumB
VDXGUYU 4022	TRANSBIG	VDXGUYU	4022	LumB	LumA
VDXGUYU 4033	TRANSBIG	VDXGUYU	4033	LumA	LumA
VDXGUYU 4034	TRANSBIG	VDXGUYU	4034	Basal	Basal
VDXGUYU 4036	TRANSBIG	VDXGUYU	4036	LumA	LumA
VDXGUYU 4040	TRANSBIG	VDXGUYU	4040	LumB	LumA
VDXGUYU 4041	TRANSBIG	VDXGUYU	4041	LumA	LumA
VDXGUYU 4043	TRANSBIG	VDXGUYU	4043	LumB	LumB
VDXGUYU 4044	TRANSBIG	VDXGUYU	4044	LumA	LumA
VDXGUYU 4045	TRANSBIG	VDXGUYU	4045	Basal	Basal
VDXGUYU 4046	TRANSBIG	VDXGUYU	4046	LumA	LumA
VDXGUYU 4049	TRANSBIG	VDXGUYU	4049	Her2	Her2
VDXGUYU 4052	TRANSBIG	VDXGUYU	4052	LumB	LumB
VDXGUYU 4054	TRANSBIG	VDXGUYU	4054	Basal	Her2
VDXGUYU 4056	TRANSBIG	VDXGUYU	4056	Her2	Her2
VDXGUYU 4060	TRANSBIG	VDXGUYU	4060	Her2	Her2
VDXGUYU 4061	TRANSBIG	VDXGUYU	4061	LumA	LumA
VDXGUYU 4062	TRANSBIG	VDXGUYU	4062	LumA	LumA
VDXGUYU 4067	TRANSBIG	VDXGUYU	4067	Basal	Basal
VDXGUYU 4072	TRANSBIG	VDXGUYU	4072	LumA	LumA
VDXGUYU 4073	TRANSBIG	VDXGUYU	4073	LumB	LumB
VDXGUYU 4074	TRANSBIG	VDXGUYU	4074	Basal	Basal
VDXGUYU 4075	TRANSBIG	VDXGUYU	4075	LumB	LumA
VDXGUYU 4076	TRANSBIG	VDXGUYU	4076	LumA	LumA
VDXGUYU 4080	TRANSBIG	VDXGUYU	4080	Normal	LumA
VDXGUYU 4081	TRANSBIG	VDXGUYU	4081	Basal	Basal
VDXGUYU 4087	TRANSBIG	VDXGUYU	4087	LumA	LumA
VDXGUYU 4088	TRANSBIG	VDXGUYU	4088	LumB	LumB
VDXGUYU 4096	TRANSBIG	VDXGUYU	4096	LumB	LumB
VDXGUYU 4097	TRANSBIG	VDXGUYU	4097	LumB	LumA
VDXGUYU 4098	TRANSBIG	VDXGUYU	4098	Her2	LumB
VDXGUYU 4099	TRANSBIG	VDXGUYU	4099	LumA	LumA
VDXGUYU 4105	TRANSBIG	VDXGUYU	4105	LumB	LumB
VDXIGRU 166154	TRANSBIG	VDXIGRU	166154	LumA	LumA
VDXIGRU 171150	TRANSBIG	VDXIGRU	171150	LumA	LumA
VDXIGRU 171260	TRANSBIG	VDXIGRU	171260	Basal	Basal
VDXIGRU 171558	TRANSBIG	VDXIGRU	171558	LumA	LumA
VDXIGRU 171620	TRANSBIG	VDXIGRU	171620	Basal	Basal
VDXIGRU 172086	TRANSBIG	VDXIGRU	172086	LumB	LumB
VDXIGRU 211980	TRANSBIG	VDXIGRU	211980	Her2	LumB
VDXIGRU 214603	TRANSBIG	VDXIGRU	214603	LumA	LumA
VDXIGRU 216806	TRANSBIG	VDXIGRU	216806	LumB	Her2
VDXIGRU 216939	TRANSBIG	VDXIGRU	216939	LumA	LumA
VDXIGRU 217320	TRANSBIG	VDXIGRU	217320	LumA	LumA
VDXIGRU 219139	TRANSBIG	VDXIGRU	219139	LumA	LumA
VDXIGRU 219468	TRANSBIG	VDXIGRU	219468	LumB	LumB
VDXIGRU 219490	TRANSBIG	VDXIGRU	219490	LumB	LumB
VDXIGRU 219587	TRANSBIG	VDXIGRU	219587	Basal	Basal

VDXIGRU 219863	TRANSBIG	VDXIGRU	219863	LumA	LumA
VDXIGRU 219873	TRANSBIG	VDXIGRU	219873	Basal	Basal
VDXIGRU 245392	TRANSBIG	VDXIGRU	245392	LumA	LumA
VDXIGRU 245596	TRANSBIG	VDXIGRU	245596	Her2	Her2
VDXIGRU 246047	TRANSBIG	VDXIGRU	246047	LumA	LumA
VDXIGRU 246166	TRANSBIG	VDXIGRU	246166	LumA	LumA
VDXIGRU 246381	TRANSBIG	VDXIGRU	246381	LumB	Her2
VDXIGRU 246560	TRANSBIG	VDXIGRU	246560	Basal	Basal
VDXIGRU 246821	TRANSBIG	VDXIGRU	246821	Basal	Her2
VDXIGRU 246919	TRANSBIG	VDXIGRU	246919	LumB	LumB
VDXIGRU 247000	TRANSBIG	VDXIGRU	247000	LumB	LumB
VDXIGRU 247243	TRANSBIG	VDXIGRU	247243	LumA	LumA
VDXIGRU 247401	TRANSBIG	VDXIGRU	247401	LumA	LumA
VDXIGRU 254031	TRANSBIG	VDXIGRU	254031	LumB	LumA
VDXIGRU 254485	TRANSBIG	VDXIGRU	254485	LumA	LumA
VDXIGRU 255160	TRANSBIG	VDXIGRU	255160	LumA	LumA
VDXIGRU 255627	TRANSBIG	VDXIGRU	255627	LumB	LumA
VDXIGRU 256209	TRANSBIG	VDXIGRU	256209	LumB	LumA
VDXIGRU 256734	TRANSBIG	VDXIGRU	256734	Basal	Basal
VDXIGRU 256940	TRANSBIG	VDXIGRU	256940	LumA	LumA
VDXIGRU 256975	TRANSBIG	VDXIGRU	256975	Basal	Her2
VDXIGRU 271448	TRANSBIG	VDXIGRU	271448	LumA	LumA
VDXIGRU 271982	TRANSBIG	VDXIGRU	271982	LumB	LumB
VDXIGRU 272219	TRANSBIG	VDXIGRU	272219	Her2	LumB
VDXIGRU 272823	TRANSBIG	VDXIGRU	272823	LumB	LumB
VDXIGRU 280328	TRANSBIG	VDXIGRU	280328	LumA	LumA
VDXIGRU 280352	TRANSBIG	VDXIGRU	280352	LumA	LumA
VDXIGRU 280775	TRANSBIG	VDXIGRU	280775	Basal	Basal
VDXIGRU 287897	TRANSBIG	VDXIGRU	287897	LumA	LumA
VDXIGRU 306818	TRANSBIG	VDXIGRU	306818	LumA	LumA
VDXIGRU 307251	TRANSBIG	VDXIGRU	307251	Basal	Basal
VDXIGRU 307327	TRANSBIG	VDXIGRU	307327	LumA	LumA
VDXIGRU 307506	TRANSBIG	VDXIGRU	307506	Basal	Her2
VDXIGRU 307829	TRANSBIG	VDXIGRU	307829	LumA	LumA
VDXIGRU 314827	TRANSBIG	VDXIGRU	314827	Basal	Basal
VDXOXFU 37	TRANSBIG	VDXOXFU	37	LumA	LumB
VDXOXFU 57	TRANSBIG	VDXOXFU	57	Normal	LumA
VDXOXFU 104	TRANSBIG	VDXOXFU	104	LumB	LumB
VDXOXFU 157	TRANSBIG	VDXOXFU	157	Her2	LumB
VDXOXFU 194	TRANSBIG	VDXOXFU	194	Basal	Basal
VDXOXFU 220	TRANSBIG	VDXOXFU	220	LumA	LumA
VDXOXFU 245	TRANSBIG	VDXOXFU	245	Her2	Her2
VDXOXFU 316	TRANSBIG	VDXOXFU	316	Basal	Basal
VDXOXFU 320	TRANSBIG	VDXOXFU	320	Basal	Her2
VDXOXFU 360	TRANSBIG	VDXOXFU	360	Basal	Her2
VDXOXFU 366	TRANSBIG	VDXOXFU	366	Her2	Her2
VDXOXFU 513	TRANSBIG	VDXOXFU	513	LumA	LumA
VDXOXFU 530	TRANSBIG	VDXOXFU	530	LumB	LumB
VDXOXFU 544	TRANSBIG	VDXOXFU	544	LumA	LumA
VDXOXFU 547	TRANSBIG	VDXOXFU	547	Basal	Basal
VDXOXFU 662	TRANSBIG	VDXOXFU	662	Her2	Her2
VDXOXFU 869	TRANSBIG	VDXOXFU	869	LumA	LumA
VDXOXFU 1006	TRANSBIG	VDXOXFU	1006	Basal	Basal
VDXOXFU 1065	TRANSBIG	VDXOXFU	1065	LumA	LumA

VDXOXFU 1183	TRANSBIG	VDXOXFU	1183	LumB	LumA
VDXOXFU 1210	TRANSBIG	VDXOXFU	1210	Normal	LumA
VDXOXFU 1328	TRANSBIG	VDXOXFU	1328	LumA	LumA
VDXOXFU 1373	TRANSBIG	VDXOXFU	1373	Basal	Basal
VDXOXFU 1415	TRANSBIG	VDXOXFU	1415	Basal	Normal
VDXKIU 121	TRANSBIG	VDXKIU	121	LumA	LumA
VDXKIU 205	TRANSBIG	VDXKIU	205	LumA	LumA
VDXKIU 386	TRANSBIG	VDXKIU	386	LumA	LumA
VDXKIU 440	TRANSBIG	VDXKIU	440	LumA	LumA
VDXKIU 1000	TRANSBIG	VDXKIU	1000	Basal	Her2
VDXKIU 1246	TRANSBIG	VDXKIU	1246	LumA	LumA
VDXKIU 1608	TRANSBIG	VDXKIU	1608	LumA	LumA
VDXKIU 1708	TRANSBIG	VDXKIU	1708	Basal	Her2
VDXKIU 2481	TRANSBIG	VDXKIU	2481	Her2	LumB
VDXKIU 2656	TRANSBIG	VDXKIU	2656	LumB	LumB
VDXKIU 2743	TRANSBIG	VDXKIU	2743	Her2	Her2
VDXKIU 2889	TRANSBIG	VDXKIU	2889	LumA	LumA
VDXKIU 2946	TRANSBIG	VDXKIU	2946	LumB	LumA
VDXKIU 3367	TRANSBIG	VDXKIU	3367	Basal	Her2
VDXKIU 101B88	TRANSBIG	VDXKIU	101B88	Basal	Basal
VDXKIU 125B43	TRANSBIG	VDXKIU	125B43	LumB	LumB
VDXKIU 136B04	TRANSBIG	VDXKIU	136B04	LumA	LumA
VDXKIU 144B49	TRANSBIG	VDXKIU	144B49	LumA	LumA
VDXKIU 15C5	TRANSBIG	VDXKIU	15C5	Her2	Her2
VDXKIU 15D6	TRANSBIG	VDXKIU	15D6	LumB	LumA
VDXKIU 15D7	TRANSBIG	VDXKIU	15D7	LumA	LumA
VDXKIU 15D9	TRANSBIG	VDXKIU	15D9	Her2	LumB
VDXKIU 15E2	TRANSBIG	VDXKIU	1500	Basal	Basal
VDXKIU 15E6	TRANSBIG	VDXKIU	15000000	Her2	Her2
VDXKIU 15E7	TRANSBIG	VDXKIU	150000000	Basal	Basal
VDXKIU 15E8	TRANSBIG	VDXKIU	1500000000	Basal	Basal
VDXKIU 15H3	TRANSBIG	VDXKIU	15H3	Basal	Basal
VDXKIU 15H4	TRANSBIG	VDXKIU	15H4	LumA	LumA
VDXKIU 16B4	TRANSBIG	VDXKIU	16B4	Basal	Basal
VDXKIU 16C8	TRANSBIG	VDXKIU	16C8	Normal	LumA
VDXKIU 16G1	TRANSBIG	VDXKIU	16G1	LumB	LumB
VDXKIU 197B95	TRANSBIG	VDXKIU	197B95	LumB	LumB
VDXKIU 199B55	TRANSBIG	VDXKIU	199B55	LumA	LumA
VDXKIU 219C42	TRANSBIG	VDXKIU	219C42	Her2	Her2
VDXKIU 220C70	TRANSBIG	VDXKIU	220C70	LumA	LumA
VDXKIU 231C80	TRANSBIG	VDXKIU	231C80	LumA	LumA
VDXKIU 247C76	TRANSBIG	VDXKIU	247C76	LumA	LumA
VDXKIU 248C91	TRANSBIG	VDXKIU	248C91	LumA	LumA
VDXKIU 266C51	TRANSBIG	VDXKIU	266C51	LumA	LumA
VDXKIU 268C87	TRANSBIG	VDXKIU	268C87	Basal	Basal
VDXKIU 279C61	TRANSBIG	VDXKIU	279C61	Basal	Basal
VDXKIU 27B8	TRANSBIG	VDXKIU	27B8	LumA	LumA
VDXKIU 27C4	TRANSBIG	VDXKIU	27C4	LumA	LumA
VDXKIU 27D5	TRANSBIG	VDXKIU	27D5	LumB	LumB
VDXKIU 282C51	TRANSBIG	VDXKIU	282C51	LumA	LumA
VDXKIU 284C63	TRANSBIG	VDXKIU	284C63	LumA	LumA
VDXKIU 28C76	TRANSBIG	VDXKIU	28C76	LumA	LumA
VDXKIU 292C66	TRANSBIG	VDXKIU	292C66	LumA	LumA
VDXKIU 309C49	TRANSBIG	VDXKIU	309C49	LumA	LumA

VDXKIU 43C47	TRANSBIG	VDXKIU	43C47	LumA	LumA
VDXKIU 87A79	TRANSBIG	VDXKIU	87A79	LumA	LumA
VDXRHU 302	TRANSBIG	VDXRHU	302	LumB	LumA
VDXRHU 317	TRANSBIG	VDXRHU	317	LumA	LumA
VDXRHU 329	TRANSBIG	VDXRHU	329	LumB	LumA
VDXRHU 410	TRANSBIG	VDXRHU	410	LumA	LumA
VDXRHU 535	TRANSBIG	VDXRHU	535	LumB	LumB
VDXRHU 1207	TRANSBIG	VDXRHU	1207	LumA	LumA
VDXRHU 1246	TRANSBIG	VDXRHU	1246	Her2	Her2
VDXRHU 1315	TRANSBIG	VDXRHU	1315	LumB	LumB
VDXRHU 1472	TRANSBIG	VDXRHU	1472	LumA	LumA
VDXRHU 1496	TRANSBIG	VDXRHU	1496	Basal	Her2
VDXRHU 1522	TRANSBIG	VDXRHU	1522	Her2	LumB
VDXRHU 1526	TRANSBIG	VDXRHU	1526	LumB	LumB
VDXRHU 1549	TRANSBIG	VDXRHU	1549	Her2	Her2
VDXRHU 1568	TRANSBIG	VDXRHU	1568	Basal	Her2
VDXRHU 1720	TRANSBIG	VDXRHU	1720	Her2	LumB
VDXRHU 1721	TRANSBIG	VDXRHU	1721	LumA	LumA
VDXRHU 1745	TRANSBIG	VDXRHU	1745	Basal	LumA
VDXRHU 1767	TRANSBIG	VDXRHU	1767	Her2	Her2
VDXRHU 1771	TRANSBIG	VDXRHU	1771	LumB	LumB
VDXRHU 1917	TRANSBIG	VDXRHU	1917	Her2	Her2
VDXRHU 1919	TRANSBIG	VDXRHU	1919	Her2	Her2
VDXRHU 1959	TRANSBIG	VDXRHU	1959	LumA	LumA
VDXRHU 1970	TRANSBIG	VDXRHU	1970	LumB	LumB
VDXRHU 2039	TRANSBIG	VDXRHU	2039	LumB	LumB
VDXRHU 2248	TRANSBIG	VDXRHU	2248	LumA	LumA
VDXRHU 2320	TRANSBIG	VDXRHU	2320	LumB	LumA
VDXRHU 2393	TRANSBIG	VDXRHU	2393	LumA	LumA
VDXRHU 2587	TRANSBIG	VDXRHU	2587	Her2	Her2
VDXRHU 3642	TRANSBIG	VDXRHU	3642	LumB	LumB
VDXRHU 4188	TRANSBIG	VDXRHU	4188	Basal	Basal
VDXRHU 5223	TRANSBIG	VDXRHU	5223	LumA	LumA
VDXRHU 5226	TRANSBIG	VDXRHU	5226	LumA	LumA
VDXRHU 5228	TRANSBIG	VDXRHU	5228	Basal	Basal
VDXRHU 5232	TRANSBIG	VDXRHU	5232	Basal	Basal
VDXRHU 5235	TRANSBIG	VDXRHU	5235	LumB	LumB
VDXRHU 5236	TRANSBIG	VDXRHU	5236	LumA	LumA
VDXRHU 5240	TRANSBIG	VDXRHU	5240	Basal	Basal
OXFU 12	UNT	OXFU	12	LumA	LumA
OXFU 1248	UNT	OXFU	1248	LumA	LumA
OXFU 126	UNT	OXFU	126	LumB	LumB
OXFU 127	UNT	OXFU	127	LumA	LumA
OXFU 1286	UNT	OXFU	1286	LumB	Normal
OXFU 138	UNT	OXFU	138	LumB	LumB
OXFU 145	UNT	OXFU	145	LumA	LumA
OXFU 16	UNT	OXFU	16	LumB	LumA
OXFU 1605	UNT	OXFU	1605	LumA	LumA
OXFU 181	UNT	OXFU	181	LumB	LumB
OXFU 217	UNT	OXFU	217	Her2	Her2
OXFU 223	UNT	OXFU	223	LumB	LumB
OXFU 23	UNT	OXFU	23	LumA	LumA
OXFU 247	UNT	OXFU	247	Her2	Her2
OXFU 254	UNT	OXFU	254	LumA	LumA

OXFU 281	UNT	OXFU	281	Basal	Basal
OXFU 337	UNT	OXFU	337	Normal	Normal
OXFU 348	UNT	OXFU	348	LumB	LumB
OXFU 359	UNT	OXFU	359	Her2	Her2
OXFU 373	UNT	OXFU	373	LumA	LumA
OXFU 382	UNT	OXFU	382	LumA	LumA
OXFU 397	UNT	OXFU	397	LumA	LumA
OXFU 419	UNT	OXFU	419	Basal	Basal
OXFU 446	UNT	OXFU	446	Normal	LumA
OXFU 449	UNT	OXFU	449	LumB	LumB
OXFU 471	UNT	OXFU	471	Normal	LumA
OXFU 476	UNT	OXFU	476	Basal	Basal
OXFU 484	UNT	OXFU	484	LumA	LumA
OXFU 485	UNT	OXFU	485	LumA	LumA
OXFU 489	UNT	OXFU	489	LumA	LumA
OXFU 491	UNT	OXFU	491	LumA	LumA
OXFU 522	UNT	OXFU	522	LumB	LumB
OXFU 53	UNT	OXFU	53	LumA	LumA
OXFU 531	UNT	OXFU	531	LumA	LumA
OXFU 533	UNT	OXFU	533	LumA	LumA
OXFU 535	UNT	OXFU	535	LumA	LumA
OXFU 543	UNT	OXFU	543	Basal	Her2
OXFU 549	UNT	OXFU	549	LumA	LumA
OXFU 557	UNT	OXFU	557	Normal	LumA
OXFU 559	UNT	OXFU	559	Her2	Her2
OXFU 573	UNT	OXFU	573	LumB	LumA
OXFU 598	UNT	OXFU	598	LumB	LumB
OXFU 608	UNT	OXFU	608	LumB	LumB
OXFU 88	UNT	OXFU	88	Her2	Her2
OXFU 885	UNT	OXFU	885	Normal	Normal
OXFU 90	UNT	OXFU	90	LumA	LumA
OXFU 93	UNT	OXFU	93	LumB	LumA
KIU 105B13	UNT	KIU	105B13	LumA	LumA
KIU 106B55	UNT	KIU	106B55	LumA	LumA
KIU 111B51	UNT	KIU	111B51	LumB	LumB
KIU 113B11	UNT	KIU	113B11	LumB	LumB
KIU 120B73	UNT	KIU	120B73	LumB	Her2
KIU 124B25	UNT	KIU	124B25	LumA	LumA
KIU 127B00	UNT	KIU	127B00	LumB	Her2
KIU 134B33	UNT	KIU	134B33	LumB	LumB
KIU 140B91	UNT	KIU	140B91	LumA	LumA
KIU 151B84	UNT	KIU	151B84	LumB	LumA
KIU 155B52	UNT	KIU	155B52	LumA	LumA
KIU 163B27	UNT	KIU	163B27	LumB	LumA
KIU 164B81	UNT	KIU	164B81	Her2	Her2
KIU 172B19	UNT	KIU	172B19	LumB	LumB
KIU 177B67	UNT	KIU	177B67	LumA	LumA
KIU 184B38	UNT	KIU	184B38	LumA	LumA
KIU 188B13	UNT	KIU	188B13	Her2	Her2
KIU 196B81	UNT	KIU	196B81	LumA	LumA
KIU 205B99	UNT	KIU	205B99	LumB	LumB
KIU 227C50	UNT	KIU	227C50	LumA	LumA
KIU 229C44	UNT	KIU	229C44	LumA	LumA
KIU 233C91	UNT	KIU	233C91	LumA	LumA

KIU 242C21	UNT	KIU	242C21	LumB	LumB
KIU 243C70	UNT	KIU	243C70	LumA	LumA
KIU 24C30	UNT	KIU	24C30	LumA	LumA
KIU 259C74	UNT	KIU	259C74	LumA	LumA
KIU 260C91	UNT	KIU	260C91	LumB	LumB
KIU 272C88	UNT	KIU	272C88	LumA	LumA
KIU 278C80	UNT	KIU	278C80	Her2	Her2
KIU 280C43	UNT	KIU	280C43	LumA	LumA
KIU 286C91	UNT	KIU	286C91	LumA	LumA
KIU 303C36	UNT	KIU	303C36	Basal	Basal
KIU 304C89	UNT	KIU	304C89	LumA	LumA
KIU 308C93	UNT	KIU	308C93	Basal	Basal
KIU 314B55	UNT	KIU	314B55	Basal	Basal
KIU 316C64	UNT	KIU	316C64	LumA	LumA
KIU 36C17	UNT	KIU	36C17	LumA	LumA
KIU 42C67	UNT	KIU	42C67	LumB	LumA
KIU 52A90	UNT	KIU	52A90	LumA	LumA
KIU 5B97	UNT	KIU	5B97	Her2	Her2
KIU 65A68	UNT	KIU	65A68	LumA	LumA
KIU 74A63	UNT	KIU	74A63	LumA	LumB
KIU 86A40	UNT	KIU	86A40	Basal	Basal
KIU 88A67	UNT	KIU	88A67	LumB	LumB
KIU 89A64	UNT	KIU	89A64	LumB	Her2
UPP 103B41	UPP	UPPT	103B41	Basal	Basal
UPP 104B91	UPP	UPPT	104B91	LumB	LumB
UPP 112B55	UPP	UPPT	112B55	LumB	LumB
UPP 114B68	UPP	UPPT	114B68	LumB	LumB
UPP 130B92	UPP	UPPT	130B92	LumA	LumA
UPP 131B79	UPP	UPPT	131B79	LumB	LumB
UPP 135B40	UPP	UPPT	135B40	LumA	LumA
UPP 138B34	UPP	UPPT	138B34	LumA	LumA
UPP 139B03	UPP	UPPT	139B03	LumB	LumB
UPP 147B19	UPP	UPPT	147B19	Her2	Her2
UPP 148B49	UPP	UPPT	148B49	Normal	LumA
UPP 14B98	UPP	UPPT	14B98	LumB	LumA
UPP 150B81	UPP	UPPT	150B81	LumB	LumA
UPP 154B42	UPP	UPPT	154B42	LumB	LumB
UPP 159B47	UPP	UPPT	159B47	LumB	LumB
UPP 15C94	UPP	UPPT	15C94	LumA	LumA
UPP 162B98	UPP	UPPT	162B98	LumB	LumB
UPP 165B72	UPP	UPPT	165B72	LumB	LumB
UPP 166B79	UPP	UPPT	166B79	LumB	LumB
UPP 169B79	UPP	UPPT	169B79	LumB	LumB
UPP 170B15	UPP	UPPT	170B15	LumA	LumA
UPP 171B77	UPP	UPPT	171B77	LumB	LumB
UPP 173B43	UPP	UPPT	173B43	LumA	LumA
UPP 17C40	UPP	UPPT	17C40	LumA	LumA
UPP 183B75	UPP	UPPT	183B75	LumB	LumB
UPP 186B22	UPP	UPPT	186B22	LumB	LumB
UPP 189B83	UPP	UPPT	189B83	Her2	Her2
UPP 191B79	UPP	UPPT	191B79	LumB	LumB
UPP 193B72	UPP	UPPT	193B72	Basal	Her2
UPP 194B60	UPP	UPPT	194B60	Her2	Her2
UPP 202B44	UPP	UPPT	202B44	Normal	LumA

UPP 203B49	UPP	UPPT	203B49	Normal	LumA
UPP 207C08	UPP	UPPT	207C08	LumA	LumA
UPP 212C21	UPP	UPPT	212C21	LumA	LumA
UPP 216C61	UPP	UPPT	216C61	LumA	LumB
UPP 225C52	UPP	UPPT	225C52	LumB	LumB
UPP 230C47	UPP	UPPT	230C47	Her2	LumB
UPP 235C20	UPP	UPPT	235C20	LumB	LumB
UPP 238C87	UPP	UPPT	238C87	Basal	Her2
UPP 239C32	UPP	UPPT	239C32	LumB	LumA
UPP 244C89	UPP	UPPT	244C89	LumB	LumB
UPP 245C22	UPP	UPPT	245C22	LumB	Her2
UPP 252C64	UPP	UPPT	252C64	Basal	Her2
UPP 254C80	UPP	UPPT	254C80	LumA	LumA
UPP 256C45	UPP	UPPT	256C45	Her2	Her2
UPP 271C71	UPP	UPPT	271C71	Her2	Her2
UPP 27C82	UPP	UPPT	27C82	LumA	LumA
UPP 288C57	UPP	UPPT	288C57	LumA	LumA
UPP 289C75	UPP	UPPT	289C75	LumA	LumA
UPP 291C17	UPP	UPPT	291C17	Her2	Her2
UPP 298C47	UPP	UPPT	298C47	LumB	LumB
UPP 301C66	UPP	UPPT	301C66	Basal	Her2
UPP 307C50	UPP	UPPT	307C50	LumB	LumB
UPP 311A27	UPP	UPPT	311A27	Her2	Her2
UPP 313A87	UPP	UPPT	313A87	LumB	LumB
UPP 35C29	UPP	UPPT	35C29	Normal	LumA
UPP 39C24	UPP	UPPT	39C24	LumA	LumA
UPP 40C57	UPP	UPPT	40C57	LumA	LumB
UPP 41C65	UPP	UPPT	41C65	LumA	LumA
UPP 46A25	UPP	UPPT	46A25	LumB	LumB
UPP 48A46	UPP	UPPT	48A46	LumA	LumA
UPP 51A98	UPP	UPPT	51A98	LumB	LumB
UPP 53A06	UPP	UPPT	53A06	LumB	LumB
UPP 55A79	UPP	UPPT	55A79	LumB	LumB
UPP 56A94	UPP	UPPT	56A94	Normal	LumA
UPP 63A62	UPP	UPPT	63A62	LumA	LumA
UPP 64A59	UPP	UPPT	64A59	LumB	LumB
UPP 67A43	UPP	UPPT	67A43	Her2	Her2
UPP 69A93	UPP	UPPT	69A93	Basal	Her2
UPP 6B85	UPP	UPPT	6B85	LumB	LumB
UPP 72A92	UPP	UPPT	72A92	LumA	LumA
UPP 76A44	UPP	UPPT	76A44	LumB	LumB
UPP 77A50	UPP	UPPT	77A50	LumA	LumB
UPP 79A35	UPP	UPPT	79A35	LumB	LumB
UPP 7B96	UPP	UPPT	7B96	LumB	LumB
UPP 82A83	UPP	UPPT	82A83	LumB	LumB
UPP 84A44	UPP	UPPT	84A44	LumB	LumB
UPP 85A03	UPP	UPPT	85A03	Her2	LumB
UPP 8B87	UPP	UPPT	8B87	LumA	LumA
UPP 96A21	UPP	UPPT	96A21	LumA	LumA
UPP 100B08	UPP	UPPU	100B08	LumA	LumA
UPP 102B06	UPP	UPPU	102B06	Her2	Her2
UPP 10B88	UPP	UPPU	10B88	LumB	LumB
UPP 110B34	UPP	UPPU	110B34	Basal	Her2
UPP 11B47	UPP	UPPU	11B47	LumA	LumA

UPP 122B81	UPP	UPPU	122B81	LumA	LumA
UPP 128B48	UPP	UPPU	128B48	LumA	LumA
UPP 137B88	UPP	UPPU	137B88	Her2	LumB
UPP 13B79	UPP	UPPU	13B79	LumA	LumA
UPP 142B05	UPP	UPPU	142B05	Her2	Her2
UPP 143B81	UPP	UPPU	143B81	Her2	Her2
UPP 145B10	UPP	UPPU	145B10	LumB	Her2
UPP 146B39	UPP	UPPU	146B39	Basal	Her2
UPP 152B99	UPP	UPPU	152B99	LumB	LumA
UPP 153B09	UPP	UPPU	153B09	Basal	Basal
UPP 156B01	UPP	UPPU	156B01	Normal	LumA
UPP 158B84	UPP	UPPU	158B84	Normal	LumA
UPP 160B16	UPP	UPPU	160B16	LumA	LumA
UPP 161B31	UPP	UPPU	161B31	LumA	LumA
UPP 168B51	UPP	UPPU	168B51	LumA	LumA
UPP 16C97	UPP	UPPU	16C97	LumA	LumA
UPP 174B41	UPP	UPPU	174B41	LumA	LumA
UPP 175B72	UPP	UPPU	175B72	LumB	LumB
UPP 176B74	UPP	UPPU	176B74	Normal	LumA
UPP 178B74	UPP	UPPU	178B74	LumA	LumA
UPP 179B28	UPP	UPPU	179B28	LumB	LumA
UPP 180B38	UPP	UPPU	180B38	Normal	LumA
UPP 181B70	UPP	UPPU	181B70	Normal	Normal
UPP 182B43	UPP	UPPU	182B43	Basal	Basal
UPP 185B44	UPP	UPPU	185B44	LumA	LumA
UPP 187B36	UPP	UPPU	187B36	Her2	Her2
UPP 18C56	UPP	UPPU	18C56	LumB	LumB
UPP 192B69	UPP	UPPU	192B69	LumA	LumA
UPP 195B75	UPP	UPPU	195B75	LumB	LumB
UPP 198B90	UPP	UPPU	198B90	LumA	LumA
UPP 19C33	UPP	UPPU	19C33	LumB	Basal
UPP 200B47	UPP	UPPU	200B47	LumA	LumA
UPP 201B68	UPP	UPPU	201B68	LumA	LumA
UPP 204B85	UPP	UPPU	204B85	LumA	LumA
UPP 206C05	UPP	UPPU	206C05	LumB	LumB
UPP 208C06	UPP	UPPU	208C06	LumB	LumB
UPP 209C10	UPP	UPPU	209C10	LumA	LumA
UPP 210C72	UPP	UPPU	210C72	LumA	LumB
UPP 211C88	UPP	UPPU	211C88	LumB	LumB
UPP 213C36	UPP	UPPU	213C36	LumB	LumB
UPP 217C79	UPP	UPPU	217C79	LumA	LumA
UPP 218C29	UPP	UPPU	218C29	LumB	LumB
UPP 21C28	UPP	UPPU	21C28	LumA	LumA
UPP 221C14	UPP	UPPU	221C14	LumB	LumA
UPP 222C26	UPP	UPPU	222C26	LumB	LumB
UPP 223C51	UPP	UPPU	223C51	LumA	LumA
UPP 224C93	UPP	UPPU	224C93	Her2	LumA
UPP 226C06	UPP	UPPU	226C06	Basal	Her2
UPP 22C62	UPP	UPPU	22C62	LumA	LumA
UPP 232C58	UPP	UPPU	232C58	Her2	Her2
UPP 234C15	UPP	UPPU	234C15	LumB	LumB
UPP 236C55	UPP	UPPU	236C55	LumA	LumA
UPP 237C56	UPP	UPPU	237C56	Her2	Her2
UPP 23C52	UPP	UPPU	23C52	LumB	LumB

UPP 240C54	UPP	UPPU	240C54	LumB	LumB
UPP 241C01	UPP	UPPU	241C01	LumB	LumB
UPP 246C75	UPP	UPPU	246C75	LumA	LumA
UPP 249C42	UPP	UPPU	249C42	Her2	LumB
UPP 250C78	UPP	UPPU	250C78	Basal	Her2
UPP 251C14	UPP	UPPU	251C14	LumA	LumA
UPP 253C20	UPP	UPPU	253C20	LumA	LumA
UPP 255C06	UPP	UPPU	255C06	LumB	LumA
UPP 257C87	UPP	UPPU	257C87	LumA	LumA
UPP 258C21	UPP	UPPU	258C21	LumA	LumA
UPP 261C94	UPP	UPPU	261C94	LumA	LumA
UPP 262C85	UPP	UPPU	262C85	LumA	LumA
UPP 263C82	UPP	UPPU	263C82	LumA	LumA
UPP 265C40	UPP	UPPU	265C40	LumB	LumB
UPP 267C04	UPP	UPPU	267C04	LumA	LumB
UPP 269C68	UPP	UPPU	269C68	Basal	Basal
UPP 26C23	UPP	UPPU	26C23	Basal	Her2
UPP 270C93	UPP	UPPU	270C93	Her2	Her2
UPP 274C81	UPP	UPPU	274C81	LumA	LumA
UPP 275C70	UPP	UPPU	275C70	LumA	LumA
UPP 277C64	UPP	UPPU	277C64	LumA	LumA
UPP 283C22	UPP	UPPU	283C22	LumB	LumB
UPP 287C67	UPP	UPPU	287C67	Basal	Basal
UPP 290C91	UPP	UPPU	290C91	LumA	LumA
UPP 294C04	UPP	UPPU	294C04	Her2	LumB
UPP 296C95	UPP	UPPU	296C95	LumA	LumA
UPP 297C26	UPP	UPPU	297C26	LumA	LumA
UPP 315C75	UPP	UPPU	315C75	LumA	LumA
UPP 316C65	UPP	UPPU	316C65	LumA	LumA
UPP 33C30	UPP	UPPU	33C30	LumA	LumA
UPP 34C80	UPP	UPPU	34C80	LumA	LumA
UPP 37C06	UPP	UPPU	37C06	Her2	Her2
UPP 42C57	UPP	UPPU	42C57	LumB	LumA
UPP 44A53	UPP	UPPU	44A53	LumA	LumA
UPP 45A96	UPP	UPPU	45A96	LumB	LumB
UPP 47A87	UPP	UPPU	47A87	Her2	LumB
UPP 49A07	UPP	UPPU	49A07	LumA	LumA
UPP 50A91	UPP	UPPU	50A91	LumA	LumB
UPP 54A09	UPP	UPPU	54A09	Basal	Her2
UPP 58A50	UPP	UPPU	58A50	Her2	Her2
UPP 60A05	UPP	UPPU	60A05	Normal	Normal
UPP 61A53	UPP	UPPU	61A53	LumB	LumB
UPP 62A02	UPP	UPPU	62A02	Basal	Basal
UPP 66A84	UPP	UPPU	66A84	Her2	Her2
UPP 70A79	UPP	UPPU	70A79	Her2	Her2
UPP 73A01	UPP	UPPU	73A01	Her2	LumB
UPP 75A01	UPP	UPPU	75A01	LumB	LumB
UPP 90A63	UPP	UPPU	90A63	LumB	LumB
UPP 94A16	UPP	UPPU	94A16	LumA	LumA
UPP 99A50	UPP	UPPU	99A50	Normal	LumA
UPP 9B52	UPP	UPPU	9B52	LumB	LumB
NKI 4	NKI	NKI	4	LumB	LumA
NKI 6	NKI	NKI	6	LumA	LumA
NKI 7	NKI	NKI	7	LumA	LumA

NKI 8	NKI	NKI	8	Basal	Basal
NKI 9	NKI	NKI	9	LumB	LumA
NKI 11	NKI	NKI	11	Her2	LumB
NKI 12	NKI	NKI	12	Basal	Basal
NKI 13	NKI	NKI	13	LumB	Her2
NKI 14	NKI	NKI	14	LumA	LumA
NKI 17	NKI	NKI	17	LumA	LumA
NKI 23	NKI	NKI	23	LumA	LumA
NKI 24	NKI	NKI	24	Basal	Basal
NKI 26	NKI	NKI	26	LumA	LumA
NKI 27	NKI	NKI	27	LumA	LumA
NKI 28	NKI	NKI	28	Normal	LumA
NKI 29	NKI	NKI	29	LumA	LumA
NKI 30	NKI	NKI	30	LumA	LumA
NKI 31	NKI	NKI	31	LumA	LumA
NKI 32	NKI	NKI	32	LumB	LumB
NKI 34	NKI	NKI	34	LumB	LumB
NKI 35	NKI	NKI	35	LumB	LumB
NKI 36	NKI	NKI	36	LumB	LumA
NKI 37	NKI	NKI	37	LumB	Normal
NKI 38	NKI	NKI	38	LumB	LumA
NKI 39	NKI	NKI	39	LumA	LumA
NKI 40	NKI	NKI	40	LumB	LumB
NKI 41	NKI	NKI	41	LumB	LumA
NKI 42	NKI	NKI	42	LumA	LumA
NKI 43	NKI	NKI	43	Her2	Her2
NKI 44	NKI	NKI	44	Basal	Basal
NKI 45	NKI	NKI	45	LumA	LumA
NKI 48	NKI	NKI	48	Basal	Basal
NKI 51	NKI	NKI	51	LumB	LumA
NKI 56	NKI	NKI	56	LumB	LumA
NKI 57	NKI	NKI	57	Basal	Basal
NKI 58	NKI	NKI	58	LumB	LumB
NKI 59	NKI	NKI	59	LumB	LumB
NKI 60	NKI	NKI	60	LumA	LumA
NKI 61	NKI	NKI	61	LumA	LumA
NKI 62	NKI	NKI	62	LumB	Her2
NKI 69	NKI	NKI	69	LumB	LumB
NKI 70	NKI	NKI	70	LumA	LumA
NKI 71	NKI	NKI	71	Basal	Basal
NKI 72	NKI	NKI	72	LumB	LumB
NKI 73	NKI	NKI	73	Normal	LumA
NKI 75	NKI	NKI	75	Basal	Basal
NKI 76	NKI	NKI	76	Her2	Her2
NKI 78	NKI	NKI	78	LumB	LumB
NKI 79	NKI	NKI	79	LumB	LumB
NKI 80	NKI	NKI	80	Basal	Basal
NKI 83	NKI	NKI	83	Basal	Basal
NKI 84	NKI	NKI	84	Basal	Basal
NKI 85	NKI	NKI	85	Basal	Basal
NKI 86	NKI	NKI	86	LumB	Her2
NKI 88	NKI	NKI	88	Basal	Basal
NKI 89	NKI	NKI	89	Basal	Basal
NKI 90	NKI	NKI	90	Basal	Basal

NKI 91	NKI	NKI	91	Basal	Basal
NKI 92	NKI	NKI	92	Basal	Normal
NKI 93	NKI	NKI	93	Basal	Her2
NKI 94	NKI	NKI	94	LumA	LumA
NKI 95	NKI	NKI	95	LumA	LumA
NKI 96	NKI	NKI	96	Basal	Basal
NKI 97	NKI	NKI	97	Basal	Basal
NKI 98	NKI	NKI	98	Basal	Basal
NKI 99	NKI	NKI	99	LumA	LumA
NKI 100	NKI	NKI	100	Basal	Basal
NKI 102	NKI	NKI	102	LumB	LumB
NKI 103	NKI	NKI	103	Basal	Basal
NKI 104	NKI	NKI	104	Basal	Basal
NKI 106	NKI	NKI	106	Basal	Basal
NKI 107	NKI	NKI	107	LumB	LumB
NKI 108	NKI	NKI	108	LumB	LumB
NKI 109	NKI	NKI	109	Her2	Her2
NKI 110	NKI	NKI	110	LumB	LumB
NKI 111	NKI	NKI	111	LumB	LumB
NKI 113	NKI	NKI	113	LumA	LumA
NKI 114	NKI	NKI	114	LumA	LumA
NKI 116	NKI	NKI	116	LumA	LumA
NKI 117	NKI	NKI	117	LumB	LumA
NKI 118	NKI	NKI	118	LumB	LumA
NKI 119	NKI	NKI	119	Basal	Basal
NKI 120	NKI	NKI	120	LumA	LumA
NKI 122	NKI	NKI	122	LumA	LumA
NKI 123	NKI	NKI	123	LumA	LumA
NKI 124	NKI	NKI	124	LumA	LumA
NKI 125	NKI	NKI2	125	LumA	LumA
NKI 126	NKI	NKI2	126	LumB	LumB
NKI 127	NKI	NKI2	127	LumB	LumB
NKI 128	NKI	NKI2	128	LumB	LumA
NKI 129	NKI	NKI2	129	LumB	LumA
NKI 130	NKI	NKI2	130	Her2	Her2
NKI 131	NKI	NKI2	131	Basal	Basal
NKI 132	NKI	NKI2	132	LumB	LumB
NKI 133	NKI	NKI	133	LumA	LumA
NKI 134	NKI	NKI2	134	LumB	LumA
NKI 135	NKI	NKI2	135	Basal	Basal
NKI 136	NKI	NKI2	136	Her2	Her2
NKI 137	NKI	NKI2	137	LumA	LumA
NKI 138	NKI	NKI	138	LumA	LumA
NKI 139	NKI	NKI	139	LumA	LumA
NKI 140	NKI	NKI2	140	LumA	LumA
NKI 141	NKI	NKI	141	Her2	Her2
NKI 142	NKI	NKI2	142	LumA	LumA
NKI 144	NKI	NKI2	144	Basal	Basal
NKI 145	NKI	NKI2	145	LumA	LumA
NKI 146	NKI	NKI2	146	LumB	LumA
NKI 147	NKI	NKI2	147	Her2	Her2
NKI 148	NKI	NKI2	148	LumA	LumA
NKI 149	NKI	NKI2	149	Her2	Her2
NKI 150	NKI	NKI2	150	LumB	LumB

NKI 151	NKI	NKI2	151	LumB	LumB
NKI 153	NKI	NKI2	153	Her2	Her2
NKI 154	NKI	NKI2	154	LumA	LumA
NKI 155	NKI	NKI2	155	LumA	LumA
NKI 156	NKI	NKI2	156	LumA	LumA
NKI 157	NKI	NKI2	157	LumA	LumA
NKI 158	NKI	NKI2	158	Her2	Her2
NKI 159	NKI	NKI2	159	LumB	LumB
NKI 160	NKI	NKI2	160	LumA	LumA
NKI 161	NKI	NKI2	161	LumA	LumA
NKI 162	NKI	NKI2	162	LumB	Her2
NKI 163	NKI	NKI2	163	LumB	LumB
NKI 164	NKI	NKI2	164	Basal	Basal
NKI 165	NKI	NKI2	165	LumB	LumB
NKI 166	NKI	NKI2	166	LumB	LumB
NKI 167	NKI	NKI2	167	LumA	LumA
NKI 169	NKI	NKI2	169	LumB	LumB
NKI 170	NKI	NKI2	170	LumA	LumA
NKI 172	NKI	NKI2	172	LumB	Her2
NKI 174	NKI	NKI2	174	LumB	Her2
NKI 175	NKI	NKI2	175	Basal	Basal
NKI 176	NKI	NKI2	176	LumA	LumA
NKI 177	NKI	NKI2	177	Basal	Basal
NKI 178	NKI	NKI2	178	LumA	LumA
NKI 179	NKI	NKI2	179	Her2	Her2
NKI 180	NKI	NKI2	180	LumB	Her2
NKI 181	NKI	NKI2	181	Her2	Her2
NKI 182	NKI	NKI2	182	LumA	LumA
NKI 183	NKI	NKI2	183	LumA	LumA
NKI 184	NKI	NKI2	184	Basal	Basal
NKI 185	NKI	NKI2	185	LumB	LumA
NKI 186	NKI	NKI2	186	Basal	Basal
NKI 187	NKI	NKI2	187	LumA	LumA
NKI 188	NKI	NKI2	188	LumA	LumA
NKI 189	NKI	NKI2	189	Her2	Her2
NKI 190	NKI	NKI2	190	LumA	LumA
NKI 191	NKI	NKI2	191	LumA	LumA
NKI 192	NKI	NKI2	192	LumB	LumB
NKI 193	NKI	NKI2	193	LumA	LumA
NKI 194	NKI	NKI2	194	LumA	LumA
NKI 195	NKI	NKI2	195	Her2	Her2
NKI 196	NKI	NKI2	196	LumA	LumA
NKI 197	NKI	NKI2	197	LumA	LumA
NKI 198	NKI	NKI2	198	LumA	LumA
NKI 199	NKI	NKI2	199	Her2	Normal
NKI 200	NKI	NKI2	200	LumA	LumA
NKI 201	NKI	NKI2	201	LumA	LumA
NKI 202	NKI	NKI2	202	Basal	Basal
NKI 203	NKI	NKI2	203	Her2	LumB
NKI 205	NKI	NKI2	205	LumB	LumA
NKI 207	NKI	NKI2	207	LumA	LumA
NKI 208	NKI	NKI2	208	LumB	LumB
NKI 209	NKI	NKI2	209	LumB	LumA
NKI 210	NKI	NKI2	210	LumB	LumB

NKI 212	NKI	NKI2	212	Basal	Basal
NKI 213	NKI	NKI2	213	LumB	LumB
NKI 214	NKI	NKI2	214	LumA	LumA
NKI 215	NKI	NKI2	215	Basal	Basal
NKI 217	NKI	NKI2	217	Her2	Her2
NKI 218	NKI	NKI2	218	LumB	LumB
NKI 219	NKI	NKI2	219	LumA	LumA
NKI 220	NKI	NKI2	220	LumA	LumA
NKI 221	NKI	NKI	221	LumA	LumA
NKI 222	NKI	NKI	222	Basal	Basal
NKI 224	NKI	NKI	224	LumA	LumA
NKI 226	NKI	NKI	226	Basal	Basal
NKI 227	NKI	NKI	227	LumB	LumB
NKI 228	NKI	NKI	228	Basal	Basal
NKI 229	NKI	NKI	229	LumA	LumB
NKI 230	NKI	NKI	230	Her2	Her2
NKI 231	NKI	NKI	231	LumA	LumA
NKI 233	NKI	NKI2	233	LumA	LumA
NKI 235	NKI	NKI2	235	LumB	LumA
NKI 236	NKI	NKI2	236	Her2	Her2
NKI 237	NKI	NKI	237	Basal	Her2
NKI 238	NKI	NKI	238	Basal	Basal
NKI 239	NKI	NKI2	239	LumA	LumA
NKI 240	NKI	NKI	240	LumB	LumB
NKI 241	NKI	NKI	241	Basal	Basal
NKI 243	NKI	NKI2	243	LumA	LumA
NKI 245	NKI	NKI2	245	Basal	Her2
NKI 246	NKI	NKI2	246	Her2	Normal
NKI 247	NKI	NKI2	247	LumB	LumB
NKI 248	NKI	NKI	248	Basal	Basal
NKI 249	NKI	NKI2	249	LumB	LumB
NKI 250	NKI	NKI	250	LumA	LumA
NKI 251	NKI	NKI	251	Her2	LumB
NKI 252	NKI	NKI	252	LumB	LumB
NKI 254	NKI	NKI	254	LumB	LumB
NKI 256	NKI	NKI2	256	LumA	LumA
NKI 257	NKI	NKI2	257	Her2	LumA
NKI 258	NKI	NKI2	258	Her2	LumB
NKI 259	NKI	NKI2	259	Her2	Normal
NKI 260	NKI	NKI2	260	LumA	LumA
NKI 261	NKI	NKI2	261	LumA	LumA
NKI 263	NKI	NKI2	263	LumB	LumB
NKI 264	NKI	NKI2	264	LumB	LumA
NKI 265	NKI	NKI2	265	Basal	Basal
NKI 266	NKI	NKI2	266	LumB	Her2
NKI 267	NKI	NKI2	267	Her2	Her2
NKI 268	NKI	NKI2	268	Basal	Basal
NKI 269	NKI	NKI2	269	Basal	Basal
NKI 270	NKI	NKI2	270	Basal	Basal
NKI 271	NKI	NKI2	271	LumA	LumA
NKI 272	NKI	NKI2	272	LumB	LumA
NKI 273	NKI	NKI2	273	LumB	LumB
NKI 274	NKI	NKI2	274	LumA	LumA
NKI 275	NKI	NKI2	275	LumB	LumA

NKI 276	NKI	NKI2	276	Basal	Her2
NKI 277	NKI	NKI2	277	LumA	LumA
NKI 278	NKI	NKI2	278	LumA	LumA
NKI 280	NKI	NKI2	280	LumA	LumA
NKI 281	NKI	NKI2	281	LumA	LumA
NKI 282	NKI	NKI2	282	LumA	LumA
NKI 283	NKI	NKI2	283	LumB	LumB
NKI 284	NKI	NKI2	284	LumB	LumB
NKI 285	NKI	NKI2	285	LumA	LumA
NKI 286	NKI	NKI2	286	Her2	Her2
NKI 287	NKI	NKI2	287	LumA	LumA
NKI 288	NKI	NKI2	288	Her2	LumB
NKI 290	NKI	NKI2	290	LumA	LumA
NKI 291	NKI	NKI2	291	LumB	LumA
NKI 292	NKI	NKI	292	LumA	LumA
NKI 293	NKI	NKI2	293	LumB	LumB
NKI 294	NKI	NKI2	294	LumB	LumB
NKI 295	NKI	NKI2	295	LumA	LumA
NKI 296	NKI	NKI2	296	Her2	LumB
NKI 297	NKI	NKI2	297	LumA	LumA
NKI 298	NKI	NKI2	298	LumA	LumA
NKI 300	NKI	NKI2	300	LumB	LumA
NKI 301	NKI	NKI2	301	LumB	LumA
NKI 302	NKI	NKI2	302	LumB	LumB
NKI 303	NKI	NKI2	303	LumA	LumB
NKI 304	NKI	NKI2	304	LumA	LumA
NKI 305	NKI	NKI2	305	LumB	LumA
NKI 306	NKI	NKI2	306	LumB	LumA
NKI 307	NKI	NKI2	307	Basal	Basal
NKI 308	NKI	NKI2	308	Her2	Her2
NKI 309	NKI	NKI2	309	LumB	LumA
NKI 310	NKI	NKI2	310	Basal	Basal
NKI 311	NKI	NKI2	311	LumB	LumB
NKI 312	NKI	NKI2	312	LumB	LumA
NKI 313	NKI	NKI2	313	LumA	LumA
NKI 314	NKI	NKI2	314	LumB	LumB
NKI 315	NKI	NKI2	315	LumA	LumA
NKI 317	NKI	NKI	317	LumB	LumB
NKI 318	NKI	NKI2	318	LumA	LumA
NKI 319	NKI	NKI2	319	LumB	Her2
NKI 320	NKI	NKI2	320	LumB	Her2
NKI 321	NKI	NKI2	321	Her2	LumA
NKI 322	NKI	NKI2	322	LumB	LumB
NKI 323	NKI	NKI2	323	LumA	LumA
NKI 324	NKI	NKI2	324	Basal	Basal
NKI 325	NKI	NKI2	325	Her2	LumA
NKI 326	NKI	NKI2	326	Basal	Basal
NKI 327	NKI	NKI2	327	LumB	LumB
NKI 328	NKI	NKI2	328	LumA	LumA
NKI 329	NKI	NKI2	329	LumB	LumB
NKI 330	NKI	NKI2	330	Basal	Basal
NKI 331	NKI	NKI2	331	LumB	LumB
NKI 332	NKI	NKI2	332	Basal	Basal
NKI 333	NKI	NKI2	333	Her2	Her2

NKI 334	NKI	NKI2	334	LumA	LumA
NKI 335	NKI	NKI2	335	Basal	Basal
NKI 336	NKI	NKI2	336	LumA	LumA
NKI 337	NKI	NKI2	337	LumB	LumB
NKI 338	NKI	NKI2	338	Basal	Basal
NKI 339	NKI	NKI2	339	LumB	LumB
NKI 340	NKI	NKI2	340	LumB	LumB
NKI 341	NKI	NKI2	341	Her2	Her2
NKI 342	NKI	NKI	342	LumB	LumA
NKI 343	NKI	NKI2	343	LumA	LumA
NKI 344	NKI	NKI2	344	Basal	Basal
NKI 345	NKI	NKI2	345	LumA	LumA
NKI 346	NKI	NKI2	346	LumA	LumA
NKI 347	NKI	NKI2	347	LumA	LumA
NKI 348	NKI	NKI2	348	LumA	LumA
NKI 349	NKI	NKI2	349	LumB	LumA
NKI 350	NKI	NKI2	350	LumB	Basal
NKI 351	NKI	NKI2	351	LumB	Her2
NKI 352	NKI	NKI2	352	LumA	LumA
NKI 353	NKI	NKI2	353	LumB	LumA
NKI 354	NKI	NKI2	354	LumB	LumA
NKI 355	NKI	NKI2	355	LumA	Normal
NKI 356	NKI	NKI2	356	LumA	LumA
NKI 357	NKI	NKI2	357	LumA	LumA
NKI 358	NKI	NKI2	358	LumA	LumA
NKI 359	NKI	NKI2	359	LumA	LumA
NKI 360	NKI	NKI2	360	LumA	LumA
NKI 361	NKI	NKI2	361	LumA	LumA
NKI 362	NKI	NKI2	362	LumB	Her2
NKI 363	NKI	NKI2	363	LumB	LumA
NKI 364	NKI	NKI2	364	Her2	Normal
NKI 365	NKI	NKI2	365	LumA	LumA
NKI 366	NKI	NKI2	366	LumA	LumA
NKI 367	NKI	NKI2	367	Her2	Her2
NKI 368	NKI	NKI2	368	LumA	LumA
NKI 369	NKI	NKI2	369	Her2	LumA
NKI 370	NKI	NKI2	370	LumA	LumA
NKI 371	NKI	NKI	371	LumB	Her2
NKI 373	NKI	NKI2	373	LumA	LumA
NKI 374	NKI	NKI2	374	LumB	LumB
NKI 375	NKI	NKI2	375	Her2	LumA
NKI 377	NKI	NKI2	377	Basal	Her2
NKI 378	NKI	NKI2	378	LumB	LumB
NKI 379	NKI	NKI	379	LumB	LumB
NKI 380	NKI	NKI	380	LumB	LumA
NKI 381	NKI	NKI2	381	LumB	LumB
NKI 383	NKI	NKI2	383	LumA	LumA
NKI 385	NKI	NKI2	385	LumB	LumB
NKI 387	NKI	NKI2	387	LumB	LumA
NKI 388	NKI	NKI2	388	LumB	LumA
NKI 389	NKI	NKI2	389	LumB	Her2
NKI 390	NKI	NKI2	390	LumA	LumA
NKI 391	NKI	NKI2	391	LumA	LumA
NKI 392	NKI	NKI2	392	LumB	Her2

NKI 393	NKI	NKI2	393	LumB	LumB
NKI 394	NKI	NKI2	394	LumA	LumA
NKI 395	NKI	NKI2	395	LumA	LumA
NKI 396	NKI	NKI2	396	Her2	LumB
NKI 397	NKI	NKI	397	LumB	LumA
NKI 398	NKI	NKI	398	Basal	Basal
NKI 401	NKI	NKI	401	LumA	LumA
NKI 402	NKI	NKI2	402	Basal	Basal
NKI 403	NKI	NKI2	403	LumA	LumA
NKI 404	NKI	NKI2	404	LumA	LumA
19893 AB01778470 17038	MAQC II	BR	BR FNA M157		LumB
19893 AB01778504 17045	MAQC II	BR	BR FNA M196		LumA
19893 AB01778510 17030	MAQC II	BR	BR FNA M176		LumB
19893 AB01779182 17047	MAQC II	BR	BR FNA M214		LumA
19893 AB01779189 17018	MAQC II	BR	BR FNA M113		LumB
19893 AB01860198 17037	MAQC II	BR	BR FNA M154		Basal
19893 AB01860313 17041	MAQC II	BR	BR FNA M165		LumB
19893 AB01913188 17049	MAQC II	BR	BR FNA M212		Her2
19893 AB01913261 17092	MAQC II	BR	BR FNA M153		Basal
19893 AB01913300 16991	MAQC II	BR	BR FNA M220		LumB
19893 AB01923090 16992	MAQC II	BR	BR FNA M228		LumA
19893 AB01943851 17042	MAQC II	BR	BR FNA M177		LumB
19893 AB01983305 17031	MAQC II	BR	BR FNA M186		Her2
19893 AB01983441 17040	MAQC II	BR	BR FNA M161		LumA

19893				
AB01983443 17022 19893	MAQC II	BR	BR FNA M159	Her2
AB01983478 17035 19893	MAQC II	BR	BR FNA M130	LumA
AB01983865 17043 19893	MAQC II	BR	BR FNA M180	Basal
AB01983888 17050 19893	MAQC II	BR	BR FNA M121	Basal
AB01983892 17029 19893	MAQC II	BR	BR FNA M155	LumB
AB01983905 17019 19893	MAQC II	BR	BR FNA M128	LumA
AB01988136 17034 19893	MAQC II	BR	BR FNA M227	LumB
AB01988400 17051 19893	MAQC II	BR	BR FNA M116	
AB01988622 17021 19893	MAQC II	BR	BR FNA M156	LumA
AB01988634 17017 19893	MAQC II	BR	BR FNA M111	Basal
AB01988641 17024 19893	MAQC II	BR	BR FNA M201	LumA
AB01988665 17044 19893	MAQC II	BR	BR FNA M188	LumB
AB01988706 17039 19893	MAQC II	BR	BR FNA M158	LumA
AB01988709 17027 19893	MAQC II	BR	BR FNA M226	LumA
AB01988711 17032 19893	MAQC II	BR	BR FNA M199	Basal
AB01988712 17033 19893	MAQC II	BR	BR FNA M215	Her2
AB01988722 17036 19893	MAQC II	BR	BR FNA M136	Basal
AB01988742 17046	MAQC II	BR	BR FNA M206	Basal

19893 AB01988743 17025	MAQC II	BR	BR FNA M211	Basal
19893 AB01988746 17026	MAQC II	BR	BR FNA M217	LumA
19893 AB01988783 16990	MAQC II	BR	BR FNA M135	LumA
19893 AB02014530 17059	MAQC II	BR	BR FNA M216	LumB
19893 AB02014587 17020	MAQC II	BR	BR FNA M139	LumA
20537 AB01913201 17055	MAQC II	BR	BR FNA M117	LumA
20537 AB01913244 17054	MAQC II	BR	BR FNA M106	LumA
20537 AB01983919 17056	MAQC II	BR	BR FNA M108	LumB
20558 AB01711719 17202	MAQC II	BR	BR FNA M123	LumB
20558 AB01712163 17203	MAQC II	BR	BR FNA M126	Basal
20558 AB01724707 17204	MAQC II	BR	BR FNA M205	Basal
20979 AB01913192 16985	MAQC II	BR	BR FNA M182	LumA
20979 AB01913684 16983	MAQC II	BR	BR FNA M133	Basal
20979 AB01943782 16982	MAQC II	BR	BR FNA M179	Her2
20979 AB01988666 16986	MAQC II	BR	BR FNA M189	LumB
20979 AB01988749 16984	MAQC II	BR	BR FNA M181	LumA
23678 AB01233000 24649	MAQC II	BR	BR FNA M230	Her2
23678 AB01233000 26147	MAQC II	BR	BR FNA M230R1	Her2

23678 AB01233040 24639	MAQC II	BR	BR FNA M236	LumB
23678 AB01233040 26137	MAQC II	BR	BR FNA M236R1	LumB
23678 AB01299744 24641	MAQC II	BR	BR FNA M258	Her2
23678 AB01299744 26139	MAQC II	BR	BR FNA M258R1	Her2
23678 AB01299779 24642	MAQC II	BR	BR FNA M246	LumA
23678 AB01299779 26140	MAQC II	BR	BR FNA M246R1	LumA
23678 AB01542140 24651	MAQC II	BR	BR FNA M237	Her2
23678 AB01542140 26149	MAQC II	BR	BR FNA M237R1	Her2
23678 AB01542151 24650	MAQC II	BR	BR FNA M239	Her2
23678 AB01542151 26148	MAQC II	BR	BR FNA M239R1	Her2
23678 AB01542166 24636	MAQC II	BR	BR FNA M264	LumB
23678 AB01542166 26134	MAQC II	BR	BR FNA M264R1	LumB
23678 AB01542220 24643	MAQC II	BR	BR FNA M238	LumB
23678 AB01542220 26141	MAQC II	BR	BR FNA M238R1	LumB
23678 AB01542230 24645	MAQC II	BR	BR FNA M280	LumA
23678 AB01542230 26143	MAQC II	BR	BR FNA M280R1	LumA
23678 AB01542241 24647	MAQC II	BR	BR FNA M245	LumB
23678 AB01542241 26145	MAQC II	BR	BR FNA M245R1	LumB

23678<br>AB01562100<br>24635	MAQC II	BR	BR FNA M247	LumA
23678<br>AB01562100<br>26133	MAQC II	BR	BR FNA M247R1	LumA
23678<br>AB01562113<br>24644	MAQC II	BR	BR FNA M266	LumB
23678<br>AB01562113<br>26142	MAQC II	BR	BR FNA M266R1	LumB
23678<br>AB01562129<br>24638	MAQC II	BR	BR FNA M235	Her2
23678<br>AB01562129<br>26136	MAQC II	BR	BR FNA M235R1	Her2
23678<br>AB01562130<br>24648	MAQC II	BR	BR FNA M231	LumA
23678<br>AB01562130<br>26146	MAQC II	BR	BR FNA M231R1	LumA
23678<br>AB01562152<br>24646	MAQC II	BR	BR FNA M257	LumA
23678<br>AB01562152<br>26144	MAQC II	BR	BR FNA M257R1	LumA
23678<br>AB01562153<br>24637	MAQC II	BR	BR FNA M256	LumA
23678<br>AB01562153<br>26135	MAQC II	BR	BR FNA M256R1	LumA
23678<br>AB01562218<br>24640	MAQC II	BR	BR FNA M270	LumB
23678<br>AB01562218<br>26138	MAQC II	BR	BR FNA M270R1	LumB
24817<br>AB02260707<br>26166	MAQC II	BR	BR FNA M259	LumB
24817<br>AB02260970<br>26172	MAQC II	BR	BR FNA M301	Basal
24817<br>AB02261485<br>26161	MAQC II	BR	BR FNA M234	LumA
24817<br>AB02261505<br>26168	MAQC II	BR	BR FNA M310	Normal

24817<br>AB02261508<br>26167	MAQC II	BR	BR FNA M251	LumA
24817<br>AB02261512<br>26160	MAQC II	BR	BR FNA M260	LumA
24817<br>AB02262603<br>26162	MAQC II	BR	BR FNA M255	LumA
24817<br>AB02262619<br>26171	MAQC II	BR	BR FNA M286	Her2
24817<br>AB02262650<br>26174	MAQC II	BR	BR FNA M287	Her2
24817<br>AB02263363<br>26173	MAQC II	BR	BR FNA M309	Her2
24817<br>AB02263375<br>26169	MAQC II	BR	BR FNA M304	LumB
24817<br>AB02263389<br>26164	MAQC II	BR	BR FNA M233	Basal
24817<br>AB02263395<br>26170	MAQC II	BR	BR FNA M297	LumA
24817<br>AB02263399<br>26158	MAQC II	BR	BR FNA M295	Basal
24817<br>AB02263400<br>26163	MAQC II	BR	BR FNA M282	LumA
24817<br>AB02263405<br>26175	MAQC II	BR	BR FNA M302	LumB
24817<br>AB02263410<br>26165	MAQC II	BR	BR FNA M261	LumA
28998<br>AB02077268<br>34965	MAQC II	BR	BR FNA M333	LumB
28998<br>AB02086473<br>34898	MAQC II	BR	BR FNA M402	Basal
28998<br>AB02086494<br>34889	MAQC II	BR	BR FNA M387	LumA
28998<br>AB02086509<br>34967	MAQC II	BR	BR FNA M386	LumB
28998<br>AB02088695<br>34964	MAQC II	BR	BR FNA M322	LumB

28998 AB02090183 34891	MAQC II	BR	BR FNA M375	LumA
28998 AB02090231 34822	MAQC II	BR	BR FNA M330	LumA
28998 AB02090665 34888	MAQC II	BR	BR FNA M316	LumA
28998 AB02090710 34885	MAQC II	BR	BR FNA M331	LumB
28998 AB02091099 34966	MAQC II	BR	BR FNA ML20	Her2
28999 AB01374958 33678	MAQC II	BR	BR FNA M371R1	Her2
28999 AB01468529 33679	MAQC II	BR	BR FNA M402R1	Basal
28999 AB01469468 31425	MAQC II	BR	BR FNA M331R1	LumB
28999 AB01469507 34970	MAQC II	BR	BR FNA M375R1	LumA
28999 AB01469529 31431	MAQC II	BR	BR FNA M322R1	LumB
28999 AB01469644 31430	MAQC II	BR	BR FNA M333R1	LumB
28999 AB01469822 33681	MAQC II	BR	BR FNA M387R1	LumA
28999 AB01469842 34742	MAQC II	BR	BR FNA M386R1	LumB
28999 AB01469868 33680	MAQC II	BR	BR FNA M316R1	Her2
28999 AB01470296 31432	MAQC II	BR	BR FNA M330R1	LumA
29099 AB02088682 34899	MAQC II	BR	BR FNA M371	Her2
29539 AB01723009 35679	MAQC II	BR	BR FNA M323	LumA
29539 AB01723028 35692	MAQC II	BR	BR FNA M442	LumB

29539				
AB01723030<br>35657<br>29539	MAQC II	BR	BR FNA<br>PERU14-16	LumB
AB01723031<br>35678<br>29539	MAQC II	BR	BR FNA M523	LumA
AB01723032<br>35694<br>29539	MAQC II	BR	BR FNA M315	LumA
AB01723039<br>35684<br>29539	MAQC II	BR	BR FNA M399	LumA
AB01723040<br>35686<br>29539	MAQC II	BR	BR FNA M524	Basal
AB01723041<br>35689<br>29539	MAQC II	BR	BR FNA M353	LumB
AB01723043<br>35685<br>29539	MAQC II	BR	BR FNA M534	LumA
AB01723044<br>35687<br>29539	MAQC II	BR	BR FNA M447	LumB
AB01723056<br>35693<br>29539	MAQC II	BR	BR FNA M356	LumA
AB01833495<br>35688<br>29539	MAQC II	BR	BR FNA M367	Her2
AB01833504<br>35681<br>29539	MAQC II	BR	BR FNA M120	Basal
AB01833515<br>35616<br>29539	MAQC II	BR	BR FNA M482	LumB
AB01833522<br>35706<br>29539	MAQC II	BR	BR FNA PERU11	Basal
AB01833526<br>35614<br>29539	MAQC II	BR	BR FNA M141	LumB
AB01833535<br>35695<br>29539	MAQC II	BR	BR FNA M107	LumA
AB01833542<br>35683<br>29539	MAQC II	BR	BR FNA M343	LumA
AB01833699<br>35605<br>29539	MAQC II	BR	BR FNA M485	Basal
AB01833716<br>35658	MAQC II	BR	BR FNA M373	Basal

29539				
AB01833728 35659 29539	MAQC II	BR	BR FNA M463	LumA
AB01833732 35677 29539	MAQC II	BR	BR FNA M357	LumA
AB01833733 35649 29539	MAQC II	BR	BR FNA PERU08	Normal
AB01833741 35650 29539	MAQC II	BR	BR FNA PERU12-14	LumB
AB01833747 35697 29539	MAQC II	BR	BR FNA M384	LumA
AB01833749 35607 29539	MAQC II	BR	BR FNA M497	LumB
AB01833754 35654 29539	MAQC II	BR	BR FNA PERU07	LumB
AB01833756 35615 29539	MAQC II	BR	BR FNA M503	Basal
AB01833758 35698 29539	MAQC II	BR	BR FNA M129	LumB
AB01833759 35699 29539	MAQC II	BR	BR FNA M421	LumA
AB01833769 35700 29539	MAQC II	BR	BR FNA M146	LumA
AB01833780 35612 29539	MAQC II	BR	BR FNA M506	LumB
AB01833820 35655 29539	MAQC II	BR	BR FNA PERU09	LumB
AB01833821 35682 29539	MAQC II	BR	BR FNA M525	LumB
AB01833829 35611 29539	MAQC II	BR	BR FNA M145	LumB
AB01833832 35608 29539	MAQC II	BR	BR FNA M469	LumA
AB01833840 35610 29539	MAQC II	BR	BR FNA M484	LumA
AB01833841 35702	MAQC II	BR	BR FNA M341	LumA

29539 AB01833876 35613	MAQC II	BR	BR FNA M434	LumB
29539 AB01833931 35690	MAQC II	BR	BR FNA M334	LumA
29539 AB01833935 35648	MAQC II	BR	BR FNA PERU01	LumA
FL398-PERU53	MAQC II	BR	BR FNA PERU53	Normal
FL412-PERU55	MAQC II	BR	BR FNA PERU55	Normal
FL454-713	MAQC II	BR	BR FNA M713	Normal
U133A FL112 US120 10 13 05	MAQC II	BR	BR FNA US120	Normal
U133A FL136 US123 11 14 05	MAQC II	BR	BR FNA US123	Normal
U133A FL137 US134 11 14 05	MAQC II	BR	BR FNA US134	LumB
U133A FL15 03 17 05	MAQC II	BR	BR FNA US031	Normal
U133A FL151 US129 12 08 05	MAQC II	BR	BR FNA US129	Normal
U133A FL161 US125 01 10 06	MAQC II	BR	BR FNA US125	Normal
U133A FL175 US147 01 13 06 2	MAQC II	BR	BR FNA US147	
U133A FL32-US2 05 19 05	MAQC II	BR	BR FNA US002	
U133A FL46-314 07 08 05	MAQC II	BR	BR FNA M314	
U133A FL78 US92 09 01 05	MAQC II	BR	BR FNA US092	LumA
U133A FL80 US97 09 01 05	MAQC II	BR	BR FNA US097	Normal
U133A ROM233 06 04 04	MAQC II	BR	BR FNA M233R1	Basal
U133A ROM286 06 04 04	MAQC II	BR	BR FNA M286R1	Her2
U133A ROM302 06 04 04	MAQC II	BR	BR FNA M302R1	Her2
FL824-195	MAQC II	BR	BR FNA M195	LumA
FL825-232	MAQC II	BR	BR FNA M232	LumA
FL644-263	MAQC II	BR	BR FNA M263	LumA
FL747-281	MAQC II	BR	BR FNA M281	LumA
FL826-283	MAQC II	BR	BR FNA M283	LumA
FL573-294	MAQC II	BR	BR FNA M294	Her2
FL645-319	MAQC II	BR	BR FNA M319	LumA
28998 AB02090243 34890	MAQC II	BR	BR FNA M332	Basal
FL494-339	MAQC II	BR	BR FNA M339	Basal
FL489-340	MAQC II	BR	BR FNA M340	LumA
FL490-345	MAQC II	BR	BR FNA M345	Basal

FL495-355	MAQC II	BR	BR FNA M355	LumA
FL745-360	MAQC II	BR	BR FNA M360	Her2
FL746-363	MAQC II	BR	BR FNA M363	LumA
U133A 80 FL 080 FL256-365 04 18 06	MAQC II	BR	BR FNA M365	LumA
U133A 80 FL 090 FL266-382 04 27 06	MAQC II	BR	BR FNA M382	Her2
U133A FL58-385 07 27 05	MAQC II	BR	BR FNA M385	LumA
FL556-396	MAQC II	BR	BR FNA M396	LumA
FL498-411	MAQC II	BR	BR FNA M411	Her2
FL574-417	MAQC II	BR	BR FNA M417	LumA
U133A 80 FL 082 FL258-423 04 18 06	MAQC II	BR	BR FNA M423	Her2
FL749-425	MAQC II	BR	BR FNA M425	Her2
FL499-430	MAQC II	BR	BR FNA M430	Basal
FL557-431	MAQC II	BR	BR FNA M431	LumA
FL575-433	MAQC II	BR	BR FNA M433	LumA
U133A 80-FL-095 FL271-443 05 11 06	MAQC II	BR	BR FNA M443	Her2
FL750-486	MAQC II	BR	BR FNA M486	LumA
FL686-502	MAQC II	BR	BR FNA M502	Normal
FL646-507	MAQC II	BR	BR FNA M507	Basal
FL491-513	MAQC II	BR	BR FNA M513	Basal
FL500-531	MAQC II	BR	BR FNA M531	LumA
FL501-545	MAQC II	BR	BR FNA M545	Normal
FL796-549	MAQC II	BR	BR FNA M549	LumA
FL457-556	MAQC II	BR	BR FNA M556	LumA
FL597-557	MAQC II	BR	BR FNA M557	Normal
FL752-558	MAQC II	BR	BR FNA M558	Basal
FL598-559	MAQC II	BR	BR FNA M559	LumB
FL599-564	MAQC II	BR	BR FNA M564	Basal
FL600-566	MAQC II	BR	BR FNA M566	LumA
U133A 80-FL-206-FL370-571 08 25 06	MAQC II	BR	BR FNA M571	Her2
U133A 80-FL-110 FL286-576 05 22 06	MAQC II	BR	BR FNA M576	LumA
FL502-578	MAQC II	BR	BR FNA M578	LumB
FL503-583	MAQC II	BR	BR FNA M583	Normal
FL557-599	MAQC II	BR	BR FNA M599	LumA
FL647-607-2	MAQC II	BR	BR FNA M607	LumA
FL602-610	MAQC II	BR	BR FNA M610	LumA
FL690-612	MAQC II	BR	BR FNA M612	Basal
U133A 80-FL-131-FL299-617 06 09 06	MAQC II	BR	BR FNA M617	Basal

FL678-619	MAQC II	BR	BR FNA M619	Basal
FL755-626	MAQC II	BR	BR FNA M626	Basal
FL786 642	MAQC II	BR	BR FNA M642	LumA
FL797-643	MAQC II	BR	BR FNA M643	LumA
FL688-647	MAQC II	BR	BR FNA M647	Normal
FL604-651	MAQC II	BR	BR FNA M651	LumA
FL605-652	MAQC II	BR	BR FNA M652	LumB
FL756-657	MAQC II	BR	BR FNA M657	Basal
FL606-658	MAQC II	BR	BR FNA M658	Basal
FL827-659	MAQC II	BR	BR FNA M659	Basal
FL679-661	MAQC II	BR	BR FNA M661	Basal
FL577-662	MAQC II	BR	BR FNA M662	LumA
FL780-665	MAQC II	BR	BR FNA M665	Basal
FL559-666	MAQC II	BR	BR FNA M666	LumA
FL680-668	MAQC II	BR	BR FNA M668	LumA
FL578-669	MAQC II	BR	BR FNA M669	LumA
FL781-670	MAQC II	BR	BR FNA M670	LumA
FL560-671	MAQC II	BR	BR FNA M671	LumB
FL689-673	MAQC II	BR	BR FNA M673	Normal
FL579-675	MAQC II	BR	BR FNA M675	LumB
FL580-679	MAQC II	BR	BR FNA M679	Basal
FL783-681	MAQC II	BR	BR FNA M681	Basal
FL761-682	MAQC II	BR	BR FNA M682	LumA
FL581-683	MAQC II	BR	BR FNA M683	LumB
FL787-685	MAQC II	BR	BR FNA M685	LumA
FL561-690	MAQC II	BR	BR FNA M690	LumA
FL582-692	MAQC II	BR	BR FNA M692	Normal
FL562-693	MAQC II	BR	BR FNA M693	LumA
FL648-695	MAQC II	BR	BR FNA M695	LumB
FL607-696	MAQC II	BR	BR FNA M696	Basal
FL608-709	MAQC II	BR	BR FNA M709	Basal
FL682-710	MAQC II	BR	BR FNA M710	LumB
FL788-714	MAQC II	BR	BR FNA M714	LumA
FL609-717	MAQC II	BR	BR FNA M717	LumA
FL610-718	MAQC II	BR	BR FNA M718	Basal
FL564-723	MAQC II	BR	BR FNA M723	LumA
FL683-725	MAQC II	BR	BR FNA M725	LumB
FL611-728	MAQC II	BR	BR FNA M728	LumB
FL612-732	MAQC II	BR	BR FNA M732	Basal
FL789-734	MAQC II	BR	BR FNA M734	LumA
FL614-744	MAQC II	BR	BR FNA M744	LumB
FL685-746	MAQC II	BR	BR FNA M746	LumA
FL701-753	MAQC II	BR	BR FNA M753	LumA
FL681-765	MAQC II	BR	BR FNA M765	LumA
FL670-767	MAQC II	BR	BR FNA M767	LumA
FL658-774	MAQC II	BR	BR FNA M774	LumA
FL660-781	MAQC II	BR	BR FNA M781	LumA
FL703-782	MAQC II	BR	BR FNA M782	LumA
FL704-784	MAQC II	BR	BR FNA M784	LumB
FL799-795	MAQC II	BR	BR FNA M795	LumA
FL1141-801(2)	MAQC II	BR	BR FNA M801	LumB
FL802-806	MAQC II	BR	BR FNA M806	LumB

Table S8: Breast cancer classification results

9 Table S9 Breast cancer data sets clinical relevance

	-	+	Fisher's Exact Test
Estrogen receptor			
Luminal subtype	21	155	7.31×10^{-42}
Non-luminal subtype	93	9	
Progesterone receptor			
Luminal subtype	69	107	3.99×10^{-15}
Non-luminal subtype	88	14	
HER2 status			
Her2 subtype	8	25	5.12×10^{-13}
Non-Her2 subtype	211	34	

Table S9: GSE20194 (MAQC II BR) clinical relevance
